# Marine cosmetics and the blue bioeconomy: From sourcing to success stories

**DOI:** 10.1016/j.isci.2024.111339

**Published:** 2024-11-06

**Authors:** Ana Rotter, Despoina Varamogianni-Mamatsi, Alenka Zvonar Pobirk, Mirjam Gosenca Matjaž, Mercedes Cueto, Ana R. Díaz-Marrero, Rósa Jónsdóttir, Kolbrún Sveinsdóttir, Teresa S. Catalá, Giovanna Romano, Bahar Aslanbay Guler, Eylem Atak, Maja Berden Zrimec, Daniel Bosch, Irem Deniz, Susana P. Gaudêncio, Ernesta Grigalionyte-Bembič, Katja Klun, Luen Zidar, Anna Coll Rius, Špela Baebler, Lada Lukić Bilela, Baruch Rinkevich, Manolis Mandalakis

**Affiliations:** 1 Marine Biology Station Piran, National Institute of Biology, Fornače 41, 6330 Piran, Slovenia; 2 Institute of Marine Biology, Biotechnology and Aquaculture, Hellenic Centre for Marine Research, 71500 Heraklion, Greece; 3 University of Ljubljana, Faculty of Pharmacy, Aškerčeva cesta 7, 1000 Ljubljana, Slovenia; 4 Instituto de Productos Naturales y Agrobiología (IPNA-CSIC), 38206 La Laguna, Tenerife, Spain; 5 Matis ohf., Icelandic Food and Biotech R&D, Vinlandsleid 12, 113 Reykjavík, Iceland; 6 Faculty of Food Science and Nutrition, University of Iceland, Reykjavik, Iceland; 7 Global Society Institute, Wälderhaus, am Inselpark 19, 21109 Hamburg, Germany; 8 Organization for Science, Education and Global Society GmbH, am Inselpark 19, 21109 Hamburg, Germany; 9 Stazione Zoologica Anton Dohrn - Ecosustainable Marine Biotechnology Department, via Acton 55, 80133 Naples, Italy; 10 Faculty of Engineering Department of Bioengineering, Ege University, Izmir 35100, Turkey; 11 AlgEn, Brniciceva 29, 1231 Ljubljana, Slovenia; 12 Faculty of Engineering Department of Bioengineering, Manisa Celal Bayar University, Manisa 45119, Turkey; 13 UCIBIO-Applied Molecular Biosciences Unit, Department of Chemistry, Blue Biotechnology and Biomedicine Lab, NOVA School of Science and Technology, NOVA University of Lisbon, 2819-516 Caparica, Portugal; 14 Associate Laboratory i4HB – Institute for Health and Bioeconomy, NOVA School of Science and Technology, NOVA University Lisbon, 2819-516 Caparica, Portugal; 15 Department of Biotechnology and Systems Biology, National Institute of Biology, Večna pot 121, 1000 Ljubljana, Slovenia; 16 Department of Biology, Faculty of Science, University of Sarajevo, Zmaja od Bosne 33-35, 71 000 Sarajevo, Bosnia and Herzegovina; 17 Israel Oceanographic and Limnological Research, National Institute of Oceanography, Tel Shikmona, Haifa 3102201, Israel

**Keywords:** Earth sciences, Aquatic science, Biotechnology, Marine biotechnology, Materials science

## Abstract

As the global population continues to grow, so does the demand for longer, healthier lives and environmentally responsible choices. Consumers are increasingly drawn to naturally sourced products with proven health and wellbeing benefits. The marine environment presents a promising yet underexplored resource for the cosmetics industry, offering bioactive compounds with the potential for safe and biocompatible ingredients. This manuscript provides a comprehensive overview of the potential of marine organisms for cosmetics production, highlighting marine-derived compounds and their applications in skin/hair/oral-care products, cosmeceuticals and more. It also lays down critical safety considerations and addresses the methodologies for sourcing marine compounds, including harvesting, the biorefinery concept, use of systems biology for enhanced product development, and the relevant regulatory landscape. The review is enriched by three case studies: design of macroalgal skincare products in Iceland, establishment of a microalgal cosmetics spin-off in Italy, and the utilization of marine proteins for cosmeceutical applications.

## Graphical abstract

**Figure:**
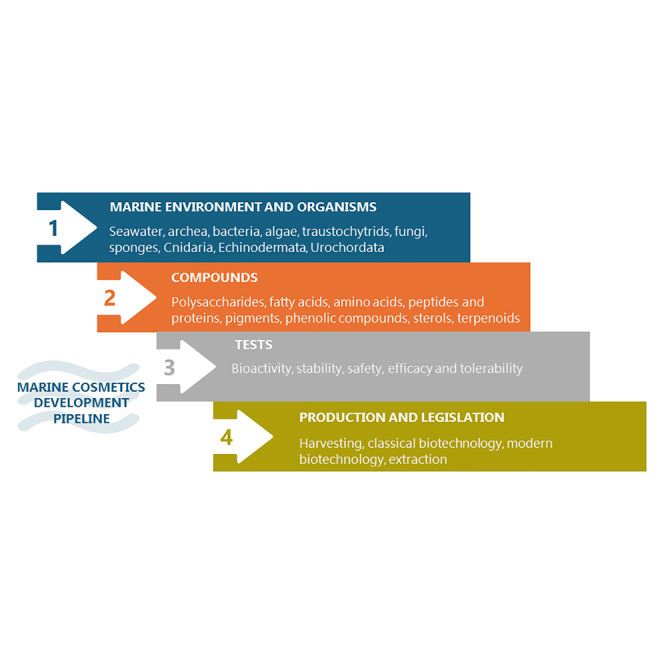

## Introduction

Health and wellbeing are pivotal challenges in modern societies. The cosmetics sector plays a central role in enhancing wellbeing by improving physical appearance with products designed for cleansing, soothing, restoring, reinforcing, and protecting the skin.^1^ At the same time, the increasing societal interest in environmental protection and green marketing trends have shifted consumer demands and behavior.^2^ This has led to an increased demand for cosmetics formulated with naturally sourced ingredients, which are considered greener and safer alternatives to conventional ones.^3^ However, natural substances are often multi-constituent mixtures, leading to two main problems in personal care products: the numerous constituents may interact unpredictably resulting in their chemical modification to harmful products and the fact that potentially toxic constituents are not disclosed in ingredient lists.^3^ This complexity can be especially problematic for consumers who opt to purchase from smaller producers, as these may lack the financial incentives for thorough safety assessments. Nevertheless, as the cosmetics market expands and global competition intensifies, scientific research and product safety are becoming indispensable development steps that producers will not be able to skip.^2^ For these reasons, it is important to take a holistic approach when considering naturally sourced ingredients, acknowledging that mere biomass cultivation or compound extraction are not enough for creating environmentally friendly products. Bringing safe, sustainable, and consumer-acceptable cosmetic products on the market requires extensive collaboration across multiple scientific disciplines, including ecology, biotechnology, toxicology, economics, regulatory science as well as social and environmental sustainability.

In this work, we introduce seven categories of cosmetics and personal care products (i.e., for skin care, sun care, hair care, oral care, decorative cosmetics, perfumes, and body care^4^) and we explore the broad potential of marine organisms/biomass in the cosmetics industry, providing a comprehensive overview rather than focusing on specific taxa. We delve into the main chemical compounds derived from marine organisms and highlight their extensive benefits for cosmetic applications. We also introduce the emerging sector of cosmeceuticals with relevant examples. This is followed by an overview of bioactivity, stability, safety, and efficacy assays that are essential across the entire cosmetics value chain and the development of cosmetic formulations. Key aspects of production and packaging processes are also outlined. Then, we provide insights into relevant legislation, sustainable bioeconomy and an overview on the bottlenecks/obstacles, thus offering essential guidance for both established and future experts in the field. Finally, three selected case studies pertaining to marine-derived cosmetics are presented: macroalgal skincare products design in Iceland, creation of a microalgal cosmetics spin-off in Italy, and the use of marine proteins for cosmeceuticals.

## Skin structure and care, including sun care, body care and perfumes

Skin is the outermost and the largest organ of the human body. It is divided into three distinct layers, each containing different types of cells and serving different functions. The top layer, the epidermis, is a keratinized stratified epithelium, which provides a watertight barrier from the external environment and prevents excessive water loss from the body. Keratinocytes make up about ∼90% of total epidermal cells and corneocytes (i.e., dead, anucleated keratinocytes) dominate the outermost layer of epidermis forming *stratum corneum*, which is a vital component of the skin’s barrier function. A smaller percentage of epidermal cells consists of melanocytes (∼8%), which produce melanin to shield against ultraviolet (UV) radiation, as well as Langerhans cells (∼3–5%) that are involved in the skin’s immune response.^5^^,^^6^^,^^7^ Dermis, the second layer of skin, is relatively thick and essential for the skin’s biomechanical properties. Its main cells are metabolically active fibroblasts that synthesize extracellular matrix (ECM) components, such as collagen and elastin fibers, providing tensile strengths and elasticity. Additional ECM elements include the consituents of the amorphous “ground substance”, namely proteoglycans (e.g., hyaluronic acid, HA) that surround connective tissue fibers, and contribute to skin viscoelasticity and hydration. Various immune cells are also present in the dermis.^8^^,^^9^^,^^10^^,^^11^^,^^12^ The deepest layer of the skin, the hypodermis (also called subcutaneous layer), consists of fat-storing cells (adipocytes) and connective tissue. The subcutaneous fat not only functions as a thermal insulation and as a protective cushion to inner organs, but it also serves as energy reserve.^5^

The skin is the first defensive line of the human body against a multitude of external factors, such as microbial pathogens and environmental stressors, including UV radiation, pollution, cigarette smoke, toxic chemicals, and metal ions. The latter can accelerate biological aging by promoting the formation of free radicals and other reactive oxygen species (ROS).^13^ ROS can damage cellular tissues and initiate complex molecular pathways including the activation of enzymes implicated in the degradation of essential ECM constituents (i.e., collagen, elastin and HA), such as metalloproteinase, collagenase, serine-protease elastase and mucopolysaccharase hyaluronidase.^11^^,^^14^ This may consequently lead to aesthetically displeasing changes in the skin, manifested as wrinkles, dryness, sallowness, deep furrows, severe atrophy, laxity, leathery appearance, telangiectasias, rough texture and variations in thickness.^15^^,^^16^ ROS can also accelerate skin pigmentation by activating the secretion of keratinocyte-derived factors, which in turn stimulate melanogenesis in melanocytes.^17^ Overall, the process of skin aging can be classified into two main types: (1) extrinsic aging caused by environmental factors like the exposure to UV radiation (photoaging) and (2) intrinsic aging, which results from natural processes in the skin by the passage of time, primarily characterized by gradual loss of elasticity.^18^^,^^19^

The market for skin and sun care products is growing, especially for multi-functional products, such as moisturizers with sun protection and products featuring natural ingredients that reduce the use of synthetic chemicals.^20^^,^^21^ Face care products still have the largest market share (over 50%), followed by body care and natural skin care products, while sun protection and baby/child products have lower revenues.^22^

## Hair care

Hair is composed of three concentric layers, namely the outer cuticle, the cortex and the inner medulla (not always present).^23^ Structurally, it is a heterodimer consisting of two α-helices of keratin, types Ia and IIa.^24^^,^^25^ With aging, progressive hair thinning occurs as a consequence of various processes including the loss of collagen type XVII.^23^ Beside providing UV protection and thermal insulation of the scalp, hair also carries significant aesthetic, cultural, sexual and beauty value.^24^ As a key element of body image, customers use hair products not only for cleansing and protection, but also for styling and altering its appearance. The hair care market is expanding, particularly in the natural products sector, driven by innovations, such as vegan hair colors and keratin products, and strategic partnerships.^26^^,^^27^

## Oral care

The oral cavity, consisting of the teeth, tongue, palate, gingiva, mucosa and lips, provides ideal conditions for microbial growth due to its relatively constant temperature and moisture levels.^28^ Oral care cosmetics are among the most technologically advanced, utilizing natural plant-based ingredients in toothpastes and mouthwashes to prevent plaque formation^28^ and incorporating nanomaterials such as nano-hydroxyapatite in products for the treatment of dental hypersensitivity and enamel remineralization.^29^ The oral care market is growing, driven by the increased awareness on the importance of oral hygiene and the growing demand for natural products.^30^

## Decorative cosmetics

This category focuses primarily on the production of pigments, which are essential for improving consumers appeal.^31^ In the cosmetics industry, pigments are predominantly produced by chemical synthesis due to their lower cost and higher stability compared to natural coloring agents.^32^ However, consumer demand for natural over synthetic colorants is driving the global natural colorants market to grow faster than the overall color market.^33^ This trend underscores the need to search for naturally sourced, stable and sustainable pigments. Although traditionally obtained from plants, the competition for plant resources as food has prompted interest in alternative natural sources, such as side streams from food production^34^^,^^35^ or marine organisms.

## The need for the development of natural skincare cosmetics

Although skin has its endogenous antioxidant defense system, comprised mainly of protective enzymes and low molecular weight antioxidants (e.g., vitamins and carotenoids),^36^ there is an increasing demand for cosmetics capable of bolstering skin protection against both internal and external harmful agents while enhancing its beauty and attractiveness.^16^ Indeed, self-image, physical appearance, health and wellbeing have always been top societal priorities.^37^ The cosmetics industry has become one of the fastest-growing sectors over the past decade,^38^ fueled by consumers seeking products that not only improve skin appearance by making it look younger and healthier, but also have a low environmental impact and adhere to cruelty-free standards (i.e., no animal testing).^39^ Consequently, there is an ongoing interest in discovering novel substances or biological extracts with antioxidant properties and inhibitory activities against skin aging-related enzymes that can be used for developing more effective antiaging dermatological products, treating skin disorders or for promoting a healthier overall physical appearance.

Currently, the cosmetics market is flooded with numerous synthetic skincare products, but many have been linked to a variety of adverse effects, such as allergic and irritant contact dermatitis, phototoxic and photoallergic reactions.^40^ Moreover, the influence of social media and effective scientific outreach have raised public awareness of the risks associated with synthetic chemicals.^41^ Therefore, there is a pressing need to discover new, safe and effective skincare ingredients.^42^ Research trends in antiaging skin care are increasingly shifting toward active ingredients of natural origin, such as plants and herbs from terrestrial ecosystems, known for their long-standing use in traditional medicine.^43^^,^^44^ Both traditional knowledge and scientific research highlight the beneficial biological effects of these natural extracts in cosmetics, including their anti-inflammatory, antioxidant, antimicrobial and antimelanogenesis properties.^45^ Given their abundance in nature, safety and relative low cost, these ingredients are attracting significant interest in the cosmetics industry.^46^

Marine organisms are an increasingly captivating reservoir of active ingredients for cosmetics, offering benefits to skin cleansing, moisturizing, antiaging, skin-firming, antipollution, anti-acne and sunscreen products.^47^^,^^48^^,^^49^ Beauty products featuring marine ingredients, such as extracts from seaweeds or other marine organisms, have gained increased popularity and present lucrative business opportunities. With the ocean being an untapped bioresource that is not competing with terrestrial resources for agri-food production, the search for and use of novel marine-derived substances and their screening for cosmetics-related bioactivities will continue to expand. However, this field will remain a subject of active research due to the need to optimize the production and purification processes for active ingredients, conduct necessary cost analyses, examine scalability of the processes and validate the feasibility, effectiveness and safety of marine-based cosmetic formulations.^50^

## Marine environment and organisms with high potential for cosmetics applications

Marine organisms have evolved biochemical and physiological mechanisms that enable them to produce bioactive compounds essential for their reproduction, communication, defense against predation, infection, and competition.^41^ Recognizing this potential, the cosmetics industry is turning its attention to marine sources for new ingredients, a trend that has gained significant momentum in recent years. Marine-based ingredients are highly valued for promoting healthy skin and providing antioxidant, anti-wrinkle, antiaging, and anti-acne benefits.^51^ As a result, the future of beauty care is expected to revolve around innovative products utilizing marine organisms.^52^ The following subsections explore the potential of seawater and various groups of marine organisms in the cosmetics industry.

### Seawater

The intrinsic properties of seawater are linked to those of water itself, the living microorganisms it contains, and the minerals and other substances dissolved in it. Seawater has a recognized role in the treatment of eczemas, dermatoses, psoriasis, nasopharyngeal inflammations, conjunctivitis, vaginitis, and other infections.^53^ In addition, it is has been used in skin care as moisturizer and to enhance skin firmness.^53^ Seawater also triggers the excretion of toxic residues and contributes to the oxygenation of tissues.^54^ Given that very few cosmetics are anhydrous (only powders, lipsticks and nail polishes), seawater can be used to replace freshwater in cosmetic formulations, provided it meets sufficient microbiological and chemical standards.^55^

### Archaea and bacteria

A great variety of molecules from marine bacteria, including carotenoids and polyphenols, have attracted interest for their cosmetic applications. Their antioxidant, anti-melanogenic and antiaging properties are increasingly being explored for inclusion in various cosmetic and pharmaceutical products.^56^^,^^57^ In the last decade, increasing attention has been directed toward molecules derived from marine microorganisms,^55^^,^^58^ especially those found in extreme environments, such as polar regions,^59^ the deep sea,^60^ and extreme halophilic habitats.^61^^,^^62^

Halophilic archaea, thriving under conditions of increased heat, UV light and salt, produce functional compounds like carotenoids and retinoids, which are widely utilized in the cosmetics industry.^63^^,^^64^ One of the most exploited archaea is *Halobacterium salinarum*, a model organism for halophilic archaea that grows in near-saturation salt conditions. This microorganism is a rich source of antioxidant and antiaging compounds including retinal (a precursor of retinoic acid), which has been shown to have anti-photoaging effects with far fewer side effects compared to other retinoids (RETINATUREL, RETINATUREL PURE, HALORUBIN OLEO by ADEKA,^65^ HALOCARE products by HALOTEC Applied Biotechnologies; Table 1).^66^

**Table 1:** Examples of archaea and bacteria and their products with (potential) application in cosmetics

Potential application	Taxa	Finding site	Compound	References
**ARCHAEA**				

Antioxidant; antiaging	*Halobacterium salinarum*	Halophilic *marine environment*	Carotenoids: bacterioruberin, bisanhydrobacterioruberin, trisanhydrobacterioruberin; RETINATUREL™; RETINATUREL™ PURE; HALORUBIN™ OLEO by ADEKA;	Mandelli et al.^67^ Halocare^66^ Chemical Products^65^
Antioxidant; antiaging	*Haloferax mediterranei*	Halophilic *marine environment*	Carotenoids	Hechler et al.^68^

**BACTERIA**				

Moisturizing	*Polaribacter* sp. SM_1127_	Polar environment	Extracellular polysaccharide (EPS)	Sun et al.^59^
	*Phyllobacterium* sp. 921F	Polar environment	EPS	Li et al.^69^
Moisturizing; Hidratation	*Pseudoalteromonas* sp.	Polar environment (Antarctic Prydz Bay sediments)	EPS RefirMAR (BIOALVO)	Li et al.^70^ Martins et al.^71^
Moisturizing; Soothing and reducing irritation of sensitive skin	*Alteromonas macleodii* ssp. *fijiensis biovar deepsan*	Deep sea hydrothermal vent	EPS HYD657 (Abyssine®) ABYSSINE™ PF by Lucas Meyer Cosmetics (IFF)	Cambon-Bonavita et al.^72^ Le Costaouëc et al.^73^
Moisturizing	*Vibrio cyclitrophicus*	Sediments	Eicosapentaenoic acid (EPA)	Abd Elrazak et al.^74^
Antiaging	*Vibrio diabolicus*	Deep-sea hydrothermal vent	Glycosaminoglycan extracellular polysaccharide (HE 800)	Courtois et al.^75^ Esposito et al.^76^
Moisturizing	*Mesoflavibacter zeaxanthinifaciens* TD-ZX30^T^	Seawater; Pacific coastline of Japan	Zeaxanthin	Asker et al.^77^
Antiaging; Pharmaceutical (treatment of age-related macular degeneration)	*Zeaxanthinibacter enoshimensis*	Seawater; Pacific coastline of Japan		Asker et al.^78^
	*Muricauda lutaonensis* CC-HSB-11^T^	Coastal hot spring of Green Island, Taiwan		Hameed et al.^79^
	*Siansivirga zeaxanthinifaciens* CC-SAMT-1^T^	Coastal seawater of Taiwan		Hameed et al.^80^
	*Aquibacter zeaxanthinifaciens* CC-AMZ-304^T^	Coastal seawater of Taiwan		Hameed et al.^81^
	*Gramella oceani* CC-AMSZ-T^T^	Marine coastal sediment, Taiwan		Hameed et al.^82^
	*Thermus filiformis*	Hot springs	Theromozeaxanthins; thermobiszeaxanthins	Mandelli et al.^67^
Antiaging	Paracoccus sp. strain *N*-8110 (=*Agrobacterium aurantiacum)*	Marine	Astaxanthin	Yokoyama et al.^83^
	*Paracoccus haeundaensis* BC74171^T^	Marine		Lee et al.^84^

Actinomycetes have been a point of interest since the chemistry of their pigments was first reported in the early 1960s.^85^^,^^86^ While plants and insects have traditionally served as the primary sources of natural pigments, microorganisms have garnered significant interest due to their bioactivities and production advantages. Notable pigments isolated from actinobacteria include prodigiosins and melanins.^86^^,^^87^^,^^88^ Beyond pigments, actinomycetes present a wide range of other bioactive compounds suitable for diverse cosmetic formulations. These compounds offer numerous benefits, including antibacterial and antifungal properties, and pigmentation enhancement.^87^^,^^88^^,^^89^

### Algae

Algae, a diverse group of aquatic organisms found in both freshwater and marine environments, have been used for centuries in various applications, including energy, food and medicine. Due to their remarkably rich bioactive composition, algae and their compounds posess several properties that make them useful in the cosmetics industry.^90^^,^^91^^,^^92^^,^^93^ Their potent natural molecules arise from the algal ability to adapt to adverse environmental conditions, such as extreme temperature, light, pressure, salinity, microbial and viral attacks.^92^^,^^94^^,^^95^

Macroalgae extracts are primarily used as active cosmetic ingredients due to their moisturizing, antiaging, photoprotective and skin whitening properties, but they can serve as excipients in the development of formulations and as additives that improve stabilization, preservation and/or the organoleptic properties of the final formulation.^49^^,^^96^ Algae-derived ingredients can purge the skin of toxins, alleviate inflammations, and exhibit bacteriostatic activity, which is particularly beneficial for acne-prone skin.^97^ Furthermore, algae-based products have been shown to possess hydrating, skin whitening and anti-wrinkle properties especially after exposure to UV radiation or cold and dry conditions, while they are effective in maintaning the skin barrier function.^98^^,^^99^^,^^100^^,^^101^^,^^102^

In Europe, around 24% of produced microalgal biomass is utilized in cosmetics.^103^ Other main uses include food supplements/nutraceuticals (24%) and feed (19%).^103^ Notably, only a few species, such as *Chlorella*, *Spirulina*, *Dunaliella*, and *Haematococcus,* have become important in industrial biotechnology, primarily due to their adaptability to large-scale production systems and their high content of bioactive compounds.

Algae contain a variety of bioactive compounds, including fatty acids, polyphenols, bromophenols, phlorotannins, terpenoids, polysaccharides, alcohols, vitamins, and vitamin precursors, most notably ascorbic acid, riboflavin, and α- β- and γ-tocopherol.^91^^,^^104^ Rich also in minerals and amino acids, algal extracts are commonly used in moisturizers, toners, and other skincare products to nourish and hydrate the skin. Microalgae/cyanobacteria from genera *Spirulina/Arthrospira*, *Chlorella*, *Haematococcus*, *Dunaliella*, *Odontella*, *Botryococcus*, *Phaeodactylum*, and *Porphyridium*, as well as macroalgae from genera *Fucus*, *Ulva*, *Laminaria*, *Gracilaria*, *Undaria*, *Sargassum*, and *Padina*, are extensively studied and utilized in cosmeceuticals due to their wide array of liposoluble vitamins, minerals, amino acids, polysaccharides, lipids, phenolic compounds, pigments, and other bioactive compounds.^91^^,^^105^^,^^106^ An extract of *Phaeodactylum tricornutum* is currently used as the base for production of specialized cosmetic ingredients with antiaging, revitalizing and anti-pollution properties, i.e., Depollutine and Megassane.^107^ Additionally, *Ecklonia maxima,* a less explored brown alga, exhibits intriguing properties for cosmetic applications including antioxidant, anti-melanogenesis, and photo-shielding effects.^108^

### Traustochytrids

Thraustochytrids, a scarcely studied eukaryotic group of single-celled protists within the class Labyrinthulomycetes (kingdom Chromista), encompass more than 12 genera and a wide range of known (>100) and undescribed strains/species^109^ with their detection often contingent upon the efficacy of the isolation protocols employed.^110^^,^^111^ Found worldwide in all estuarine and marine habitats, both on and within marine organisms, as well as in decaying organisms, traustochytrids have increasingly attracted biotechnological attention. They are considered potentially significant as reservoirs of valuable bioactive compounds, including docosahexaenoic acid (DHA, rich in omega-3 polyunsaturated fatty acids, PUFA), antioxidants like squalene, various enzymes and pigments^58^^,^^112^ which serve as well-known substitutes for synthetic antioxidants and pigments.^113^ In recent years, there has been a growing development of functional cosmetic products incorporating thraustochytrid oil, noted for its hydrating, emulsion-stabilizing, gelling and antioxidant characteristics. This surge was propelled after the oils from the genera *Schizochytrium* and *Ulkenia* were designated as “Generally Recognized as Safe” (GRAS) for human consumption by both the American Food and Drug Administration and the European Commission.^114^

### Fungi

Several marine-derived fungi produce secondary metabolites with cosmeceutical potential. For example, species such as *Phaeotheca triangularis, Trimmatostroma salinum, Hortaea werneckii, Aureobasidium pullulans*, and *Cryptococcus liquefaciens* are known to produce micosporine-like amino acids, MAAs.^115^ Additionally, the benzodiazepine alkaloids, circumdatins I, C, and G, isolated from the culture of the marine sponge-associated fungus *Exophiala* sp. (Family: Herpotrichiellaceae) displayed more potent UVA protecting activity than the positive control oxybenzone, which is currently used in sunscreen formulations.^116^ Myrothenone A and 6-*n*-pentyl-α-pyrone, isolated from the culture of the algicolous fungus *Myrothecium* sp., which was obtained from the marine green alga *Ulva compressa*, exhibited strong anti-tyrosinase activity.^117^ Similarly, cylindromicin, a secondary metabolite from the *Tolypocladium* sp. strain SCSIO 40433 isolated from Arctic glacial sediments, also showed tyrosinase inhibitor activity.^118^ Fungal extracts of *Eupenicillium crustaceum* are used in Eyedeline and Brighlette products by Lipotec for their ability to promote elastine/collagen synthesis and reduce hyperpigmentation respectively.^119^

### Sponges

Marine sponges are an exceptionally rich source of natural products with diverse chemical structures and bioactive properties. Several of these are produced by the microorganisms associated with the sponges, which are important for both sponge survival and metabolite production.^120^^,^^121^^,^^122^ These include antiaging enzymes and pigments of special interest in the cosmeceutical industry.^123^ Extracts from bacteria associated with *Scopalina hapalia* have shown potential in inhibiting elastase and tyrosinase, two enzymes involved in skin aging, and are specifically targeted for developing cosmetic products aiming at antiaging and anti-melanogenic effects. Additionally, some extracts have demonstrated considerable activation of catalase and Sirtuin 1, key targets in the discovery of antioxidants and antiaging agents.^124^ Consequently, more sponge-associated microorganisms are being studied for their potential applications in the cosmetics and cosmeceuticals sectors,^125^ with various pigments being added to the growing list of sponge-derived compounds used in the cosmetics industry.^123^ Furthermore, it has also been discovered that *Acremonium,* a genus of fungi in the family Hypocreaceae found in sponges, produces hydroquinone derivates with higher antioxidant activity and improved properties compared to their synthetic hydroquinone counterparts.^58^ In general, the sponge mesohyl is inhabited by microbes, and many natural products isolated from marine sponges, such as antibiotics, antifungal, and antipredator or antifouling compounds, have been attributed to microbial origin.^126^^,^^127^

Marine sponges also release enzymes that can be used as skin-whitening agents in several cosmetic formulations.^51^ It is also worth noting the photoprotective effects of sponge-derived alkaloids, such as topsentin isolated from *Spongosorites genitrix* that can protect human keratinocytes against UV-induced damage, indicating significant potential for inclusion in cosmetic formulations.^128^ Additionally, chitin, its derivative chitosan, and collagen from sponges are highly promising in the cosmetics industry, similarly to those derived from other marine organisms.^129^ Marine sponges also hold the potential as a natural source of marine-derived cosmeceuticals for acne prevention^130^ and other skin-related cosmetic issues.^131^

Marine sponges are also a great source of biocompatible materials for biomedicine, attracting great interest not only for their potential pharmaceutical uses but also for their biomaterials, which include chitin/chitosan, ceramic, biosilica, and collagen.^132^ Siliceous sponges (Demospongiae and Hexactinellida) are unique in their ability to enzymatically polymerize silica, forming massive siliceous skeletal elements (spicules) through this distinctive process at ambient temperature and pressure.^133^ The biomedical applications of silicatein/biosilica, particularly in treating bone/tooth defects as well as in dental care, are aimed at creating protective biosilica layers on teeth (reducing the risk of caries/cavities caused by bacteria) or promoting bone tissue regeneration through the biosilica-stimulated formation of hydroxyapatite by mineralizing cells.^134^

### Cnidaria

Cnidarians are a diverse phylum of marine invertebrates with about 11,000 species, including organisms such as jellyfish, hydroids, sea anemones and corals. They are intensively studied to identify promising bioactive compounds, including those that can be valuable to the cosmetics industry. An unexpected new source of cosmetic-related ingredients within this group are cnidarian venoms, which contain ion channel modulators with great potential for antiwrinkle treatments and the healing of sensitive skins.^135^ Other relevant compounds found in the cnidarian venoms are capable of inhibiting collagenases and proteases, offering skin firming effects, and acting as tyrosinase inhibitors, commonly used for skin tanning and whitening.^136^^,^^137^ One example is an analgesic peptide from *Heteractis crispa* (a sea anemone) that potently blocks the vanilloid receptor of the transient receptor potential channel family (a voltage gated sodium channel), inducing analgesia upon skin application, and making it a good candidate for soothing sensitive skin.^138^ Similarly, lysozymes found in jellyfish can be also suitable for treating various epidermal infections as they pose no toxic effects on humans.^139^

In addition to cnidarian toxins, there is growing interest in the sustainable harvesting of collagen (usually administered as injectable fillers or topical creams to slow down or reduce the signs of skin aging), primarily from jellyfish. Jellyfish collagen exhibits low immunogenicity and is biocompatible with mammalian tissues.^140^ In addition, many scyphomedusa species are rich in collagen that is very similar to human collagen.^141^ Jellyfish collagen and its hydrolysates have also been reported to provide protection against the impacts of UV radiation, especially by maintaining the activity of skin antioxidant enzymes.^142^ Moreover, *Aurelia aurita* jellyfish was identified as a unique source of marine collagen, noted for its high biocompatibility and denaturation temperatures.^143^ However, studies are still needed to fully elucidate the collagen contents in the Scyphozoa.^144^

Mucin, an ubiquitous cnidarian glycoprotein, has the potential to be used in cosmetic products for maintaining skin hydration and protecting the outermost skin layers,^135^ due to its ability to bind large amounts of water. Moreover, type A gelatin produced from jellyfish can serve as an emulsion stabilizer and it can be included in a wide range of cosmetic products, such as body lotions, face creams, hair sprays, sunscreens and shampoos.^145^ Other studies have shown that jellyfish collagen extract can promote the expression of beneficial enzymes in human skin keratinocytes like hyaluronan synthase-3 and aquaporin-3, which are involved in HA synthesis and water/glycerol transportation through the skin layers.^146^ Following the trend in the cosmetics industry to include antioxidants as active ingredients in its products, antioxidants from various jellyfish species have revealed interesting potential applications.^147^

Furthermore, a recent study^148^ demonstrated that exposing *Cassiopea andromeda* to narrow-band UVB radiation increases both its overall antioxidant activity and peridinin content, as a result of the enhanced photosynthetic activity of its endosymbiotic dinoflagellates, thus elevating the valorization potential of this jellyfish in the cosmetics industry.

Corals have long been utilized by the cosmetics industry. Their powder is used as a sustainable material in numerous cosmetic products due to its physical, chemical, and textural characteristics as well as its mineral content.^51^ Chemically, it primarily consists of calcium carbonate, but may also contain up to 74 other minerals. It is used in products intended for topical applications to provide minerals for the skin, protect against UV radiation and serve as an antioxidant, antiaging, antiacne and skin softening agent, while also being used for the preparation of lipsticks and deodorants.^51^ Although only a few coral-derived secondary metabolites have found use as cosmeceuticals, the diterpene glycosides pseudopterosins A–D, isolated from the Caribbean gorgonian coral *Pseudopterogorgia elisabethae*, are among the most notable marine natural products in the cosmetic industry.^149^ These compounds, commercialized as Resilience by Estée Lauder^41^^,^^149^ exhibit a variety of biological activities ranging from anti-inflammatory and analgesic,^150^^,^^151^^,^^152^ to antibacterial,^153^ antiacne,^154^ and wound healing ones.^155^^,^^156^ Additionally, the cembrene diterpenoids found in soft corals offer various biological properties relevant for the cosmetics sector. For example, Chen et al.^157^ highlighted that sinulariolides from *Sinularia flexibilis* can inhibit keratinocyte over-proliferation and sebum secretion, the latter of which is a key target for shampoo and anti-acne products.

Many cnidarians cointain MAAs, which are major UV-absorbing secondary metabolites. MMAs of corals are increasingly considered for use in sunscreening products,^158^ as they offer a natural alternative to environmentally harmful chemical-based sunscreens. These MMAs are not only environmentally friendly, but they also possess antioxidant, anti-inflammatory and antiaging properties.^159^

### Echinodermata

Sea cucumber extracts are rich in bioactive compounds such as saponins, chondroitin sulfate, collagen, vitamins A, B1, B2, B3, minerals (calcium, magnesium, iron, zinc, selenium, germanium, strontium, copper, and manganese), amino acids, phenols, triterpene glycosides, carotenoids, bioactive peptides, fatty acids, and gelatin. Found in toothpaste, ointments, body lotions, and facial skin cleansers,^160^ sea cucumber ingredients provide several health benefits, including wound healing, neuroprotective, antitumor, anticoagulant, antimicrobial, and antioxidant effects.^161^^,^^162^ Their extracts are often used to treat skin issues, wrinkles and sunburns.^163^ Moreover, sea cucumber viscera extracts have been shown to promote the expression of various enzymes (e.g., TRP-1, TRP-2, MITF, ERK) that are important in skin whitening and antiaging treatments.^164^ The vitamins and minerals from sea cucumber extracts are readily absorbed and provide a moisturizing effect, while stimulating the renewal of damaged skin cells.^165^ Notably, sea cucumbers contain considerable amounts of sulfated polysaccharides, which have great potential for the development of cosmeceuticals. Fucosylated chondroitin sulfates, a unique type of these polysaccharides isolated from the body walls of several sea cucumber species, are structurally disctinct from those found in other invertebrates, vertebrates, and algae.^166^^,^^167^^,^^168^ Similar to other marine invertebrates, sea cucumbers are a valuable source of collagen. Their body walls contain type I collagen that exhibits superior moisture-retention/absorption capacity, higher yields compared to glycerol or collagens from other animals and is very rich in hydrophilic groups, making it highly suitable for the cosmetics industry.^169^ Further studies have confirmed that aqueous extracts from sea cucumbers hold greater cosmetic potential compared to organic extracts. This is attributed to their rich content of fatty acids and antioxidants, the latter of which play a crucial role in regulating ROS production at wound sites.^41^

### Urochordata

Various solitary and compound tunicates contain bioactive compounds with the potential to be used in the pharmaceutical^170^ and cosmetics industry, especially for the development of wrinkle-care and antiaging products, as well as for the treatment of inflammation-related disorders.^171^ Scientific interest in tunicates for cosmetic uses centers on their mantle (the tunic matrix), which provides antiaging properties^172^ and a diverse range of alkaloids and peptides.^173^ The tunic matrix is composed of well-organized cellulosic microfibrils and the cellulose film derived from it does not cause toxic or immune responses,^171^ making it a safe ingredient for cosmetic products.^174^

## Compounds from marine origin for cosmetics applications

There are numerous compounds from marine organisms with proven benefits for use in the cosmetics sector (Table 2). This section presents several bioactive substances of marine origin, describing their beneficial effects on skin health and their source organisms.

**Table 2:** A non-exhaustive list of marine bioactive compounds used in cosmetics

Family of compounds	Compounds	Function	Organism	Reference
Polysaccharides	Deepsane (Abyssine®)	Skin soothing	Bacteria *(Alteromonas macleodii)*	Pereira^54^; Martins et al.^71^; Cambon-Bonavita et al.^72^
	Alguard®	Photo damaging, antiaging	Rhodophyceae (red algae): *Porphyridium* sp.	Martins et al.^71^
	Alguronic Acid®	Antiaging	Microalgae	Martins et al.^71^
	HE 800	Collagen stimulant	Bacteria *(Vibrio diabolicus)*	Corinaldesi et al.^58^; Courtois et al.^75^
	Fucoidan	Antibacterial, anticellulite, skin regeneration antioxidant	Phaeophyceae (brown algae) Sea cucumber	Pomin^167^; Fujimura et al.^175^; O’Leary et al.^176^; Sezer et al.^177^; Yu et al.^178^; Chen et al.^179^
	Laminarin	Anti-inflammatory, antioxidant, antiviral, anticellulite	Phaeophyceae (*Laminaria* spp.)	Stengel et al.^180^; Pereira et al.^181^; Fabrowska et al.^182^
	Alginate	Emulsifier, gelling agent	Phaeophyceae	Malinowska^183^
	Agarans	Antioxidant, anti-inflammatory, gelling agent	Rhodophyceae	Chen et al.^184^
	Ulvans	Antiaging, gelling agent, hydration	Chlorophyta (green algae): *Ulvales*	Fournière et al.^185^; Guidara et al.^186^
	Carrageenan, carrageenan oligosaccharides	Emulsifiers, stabilizers, thickeners, gelling agents, antioxidant	Rhodophyceae: genera *Eucheuma, Gigartina, Chondrus, Hypnea*	Pereira^53^; Campo et al.^187^; Shafie et al.^188^; Carrageenan (Explained + Products)^189^; Carrageenan in Toothpaste: What You Need to Know - Crest^190^; George^191^
	Chitin, chitosan, and derivatives	Antibacterial, anti-pigmentation agent, moisturizing	Exoskeleton of crustaceans, cnidarian, poriferan (Hexactinellida), foraminifera, marine gastropods	Bissett et al.^192^; Bissett et al.^193^; Kikuchi and Matahira^194^; INCIDecoder Acetyl Glucosamine (Explained + Products)^195^
Fatty acids	Saturated Fatty Acids	Stimulation of collagen production, anti-inflamatory, emollient	Chlorophyta: *Cladophora glomerata*	Bonnet^196^; Bialek et al.^197^; Rabasco Alvarez and González Rodríguez^198^; Zielińska and Nowak^199^; Ziboh et al.^200^
	Polyunsaturated Fatty Acids (PUFA)	Anti-inflammatory, antiallergic, antioxidant, emollient	Filamentous cyanobacteria and diatoms, Traustochytrids	Stengel et al.^180^; Malinowska et al.^183^; Dawczynski et al.^201^; Peinado et al.^202^; Venkateshwarlu et al.^203^; Fabrowska et al.^204^; Messyasz et al.^205^; Burja et al.^206^; Xie and Wang^207^; Gupta et al.^208^
Amino Acids	Arginine, glycine, alanine, valine, leucine, proline, serine, histidine, tyrosine	Moisturizing	Bacteria and algae (macro and micro)	Guillerme et al.^49^; Diaz et al.^209^; Kalasariya et al.^210^
Mycosporin-like amino acids (MAAs)	Helioguard 365®, shinorine, Helionori®, ASPAR’AGE™	Photoprotection, antiaging, antiwrinkle	Cyanobacteria: *Anabaena variabilis*, *Nostoc commune*; microalgae, red macroalgae (*Asparagopsis armata*, *Porphyra umbilicalis*), Echinodermata (sea cucumbers)	Geraldes and Pinto^211^; Helioguard^212^; Cotas et al.^213^; ASPAR’AGETM^214^
Protein	Collagen	Antiaging, antioxidant, antiwrinkle	Porifera (sponges), Cnidaria (jellyfish), Echinodermata (sea cucumbers), fish skin	Guillerme et al.^49^; Lee et al.^215^; Aguirre-Cruz et al.^216^
	Silicatein	Biosilica-mediated regeneration of tooth and bone defects	Siliceous sponges	Müller et al.^134^
	SeaCode®	Antiaging enhancing synthesis of essential dermal proteins	Bacteria (*Pseudoalteromonas* sp.)	Martins et al.^71^
	CPD-photolyases	Antiaging	Cyanobacteria (*Synechococcus leopoliensis*)	Ramírez et al.^217^; Yarosh et al.^218^; Plankton Extract^219^
	Phycobiliproteins	Antiaging, colorants, anti-inflammatory, antioxidant	Rhodophyceae	Dini^220^; Resende et al.^221^
Peptides	–	Antiaging, antioxidant, anti-inflammatory, stimulate collagen synthesis	Fish, starfish, Cyanobacteria: *Arthrospira platensis* (Spirulina) and microalgae – Chlorophyta: *Chlorella vulgaris*, *Dunaliella salina*	Han et al.^222^; Xia et al.^223^
	Dermochlorella®	Skin firmer and toner	Microalgae (*Chlorella* sp.)	Cunha and Pintado^224^
Pigments	Carotenoids: beta carotene, fucoxanthin, astaxanthin, lutein, zeaxanthin	Antioxidant, anti-inflammatory, photoprotective, regulate skin pigmentation	Bacteria; Bacillariophyceae (diatoms) genera: *Phaeodactylum*, Prymnesiophyceae (Coccolithophyceae): *Isochrysis* spp., Chlorophyta: *Chlorella* sp.*, Haematococcus pluvialis*, *Scenedesmus* sp.*, Tetraselmis* sp.; Phaeophyceae: *Laminaria digitata, L. japonica, Postelsia palmaeformis*, *Undaria* sp.*, Fucus* sp. Traustochytrids: *Thraustochytriidae, Ulkenia* sp., *Aurantiochytrium* sp.); Fungi (*Phaffia rhodozyma*)	Corinaldesi et al.^58^; Molino et al.^95^; Araújo et al.^103^; Spolaore et al.^105^; Dharmaraj et al.^225^; Dharmaraj et al.^226^; Mohammadzadeh Honarvar et al.^227^; Zhang et al.^228^; Galasso et al.^229^; Aasen et al.^230^; Khaw et al.^231^; Schüler et al.^232^; Peng et al.^233^; Heo and Jeon^234^; Shimoda et al.^235^
	Chlorophyll, phycocyanin and phycoerythrin (PE)	Antioxidant, anti-inflammatory, photoprotective	Cyanobacteria; Chlorophyceae and Rhodophyceae (chlorophyll-a and –b), Phaeophyceae (chlorophyll-c)	Hsieh-Lo et al.^236^; Li et al.^237^
	Scytonemin	UV absortion	Cyanobacteria, crustaceans	Derikvand et al.^238^; Proteau et al.^239^
	Prodigiosins	Anticancer, antibacterial, antifungal, and immunomodulation	Actinobacteria (*Streptomyces* sp.) and other bacteria	Perry^86^
	Phycobiliproteins, such as R-phycoerythrin, allophycocyanin (APC)	Antiaging, colorants, anti-inflammatory, antioxidant	Cyanobacteria (*Phormidium* sp.), Rhodophyceae	Chen et al.^240^
Melanins	Allomelanin, neuromelanin, eumelanin, pheomelanin, pyomelanin	Antioxidant, anti-inflamatory	Cephalopoda/squid ink, bacteria (*Halomonas venusta, Pseudomonas stutzeri*, *Providencia rettgeri*, *Streptomyces* sp.), Fungi (*Aspergillus nidulans*)	Kurian and Bhat^241^; Poulose et al.^242^; Shanuja et al.^243^; Kiriyachan Kurian and Ganapathy Bhat^244^
	Creanatural	Photoprotective, antioxidant	Squid ink	Creanatural®^245^
Phenolic compounds	Phlorotannins, kaempferol, quercetin, rutin	Antiallergic, anti-adipogenic, antimicrobial, antioxidant, anti-inflammatory, UV protection	Rhodophyceae (*Gracilaria dendroides*)	Heo et al.^39^; Cotas et al.^213^; Xu et al.^246^
Sterols	Fucosterol	Antioxidant, anti-inflammatory, and skin barrier-enhancing properties, reduce redness, antiaging, improve hydration	Diatoms (*Thalassiosira pseudonana, Chaetoceros muelleri*), Phaeophyceae (*Sargassum fusiforme, S. horneri, Fucus vesiculosus*)	Hannan et al.^247^
	Clerosterol	Anti-inflammatory, antioxidant	Chlorophyta (*Codium fragile*)	Lee et al.^248^
Terpenoids	Squalene/hydrogenated squalene	Emollient, antioxidant, improves skin barrier function, hydration	Microalgae (*Botryococcus braunii*), Thraustochytrids (*Aurantiochytrium acetophilum* sp.), fungi (*Penicillium brasilianum*), sea cucumbers	Kaya et al.^249^; Stoyneva-Gärtner et al.^250^
	Meroterpenoids brasilianoids A–F	Protection against UVB-induced cell damage	Fungi (*Penicillium brasilianum*)	Zhang et al.^251^; Rawlings and Harding^252^; Eckhart and Tschachler^253^
	Saponins	Hyperpigmentation and rosacea	Sea cucumbers	Correia-da-Silva et al.^254^
	Pseudopterosins A–D (Resilience®)	Anti-inflammatory, analgesic, antibacterial, antiacne, wound healing	Corals (*Pseudopterogorgia elisabethae*)	Alves et al.^41^

### Carbohydrates

Carbohydrates are a very complex and heterogeneous group of metabolites. They appear free, as polysaccharides or adhered to proteins and lipids. Marine carbohydrates, produced by photosynthetic marine organisms, are vital organic compounds serving as energy transporters and structural components.^255^ Algal carbohydrates include fucoidans, glucans, alginates, agar, porphyrin, galactans, ulvans, and carrageenans. Many have been proven to exert skin-protective effects, such as anti-wrinkling, whitening, moisturizing, UV light protective, antioxidant, and anti-inflammatory activities. Moreover, their physicochemical properties, such as the ability to form hydrogels, extend their utilization as emulsifiers, stabilizers, and viscosity-controlling ingredients in cosmeceuticals.^256^

#### Polysaccharides

Polysaccharides play an important role in cosmetics as moisturizers, emulsifiers, wound healing agents, and thickening agents,^93^ while presenting antioxidant, antimicrobial, anti-inflammatory, anticancer, and other bioactivities.^257^ They are produced by macroalgae and all microorganisms, including microalgae, proteobacteria, cyanobacteria, and archaea, but bacterial polysaccharides are some of the most used substances with an antiaging action.^49^ Polysaccharides derived from *Pseudoalteromonas* spp., *Pseudoalteromonas antarctica*, and *Halomonas eurihalina* that proliferate in Antarctic waters have been incorporated in antiaging products.^49^ With regards to algae, fucoidans, laminarins, alginates, agarans, and carrageenans are some of the most common polysaccharides.^175^ Interestingly, the sulfated polysaccharides of several seaweeds, particularly fucoidans and galactans, have been reported to be effective against *Escherichia coli* and *Staphylococcus aureus*.^258^

##### Exopolysaccharides (deepsane, HE 800, Alguard and alguronic acid)

HE 800, an exopolysaccharide analogous to HA, is produced by the deep-sea bacterium *Vibrio diabolicus* and it has the ability to stimulate collagen structuring.^58^^,^^75^ The EPS of marine bacteria have powerful moisturizing potential,^57^^,^^259^ and there has been much interest for the EPS from *Polaribacter* sp. SM1127 isolated from Arctic kelp,^59^ as well as from *Pseudoalteromonas* sp.^70^ and *Phyllobacterium* sp. 921F.^69^ Additionally, *Alteromonas macleodii* subsp. *fijiensis* biovar deepsane produces EPS HYD65, also known as “deepsane”^72^ which is used in Abyssine cosmetics (ABYSSINE PF by Lucas Meyer Cosmetics (IFF)).^71^^,^^73^ This strain was isolated from the deep-sea polychaete worm *Alvinella pompejana*,^69^ collected from a hydrothermal vent in the East Pacific at a depth of 2600 m. Alguronic acid has demonstrated major antiaging propreties, while Alguard is derived from red microalgae *Porphyridium* sp. and protects the cells in the intertidal environment. Studies showed that Alguard acts against photo damaging, aging and skin micro-abrasion.^71^

##### Fucoidans, laminarans, alginates, agarans, and ulvans

Fucoidans, commercially exploited by Takara-Bio in Japan, are known to stimulate the production of the Heparin Growth Factor, which stimulates the development of various cells and tissues. These sulfated polysaccharides accelerate fibroblast and epithelial cell growth, while also increasing TGF-β1 secretion that promotes wound healing and modulates growth factor-dependent pathways involved in tissue repairs.^176^ Moreover, fucoidan/chitosan hydrogels effectively contract the size and heal dermal burns.^177^ Topical application of fucoidan has also been shown to exert antiaging activity by increasing skin moisture and cell elasticity.^175^ Fucoidan derived from the sea cucumbers *Thelenota ananas, Apostichopus japonicus*, *Isostichopus badionotus* and *Ludwigothurea grisea* presents antioxidant properties, as well as other bioactivities, and they can be valuable in cosmetic products as antiaging agents to prevent wrinkle formation and skin photoaging.^167^^,^^178^^,^^179^

Laminarans or laminarins are β-1,3-D-glucans primarily derived from the brown seaweed *Laminaria*. These biologically active polysaccharides have antioxidant, anticoagulant, anti-inflammatory, antiviral and antitumoral activities,^180^ and anticellulite properties.^181^^,^^182^ Similarly, alginates are the main polysaccharides found in brown seaweeds (Phaeophyceae).^260^ Due to their chelating properties, alginates are widely used in cosmetics as gelling agents, thickeners, protective colloids, and emulsion stabilizers.^183^

**Figure 1:**
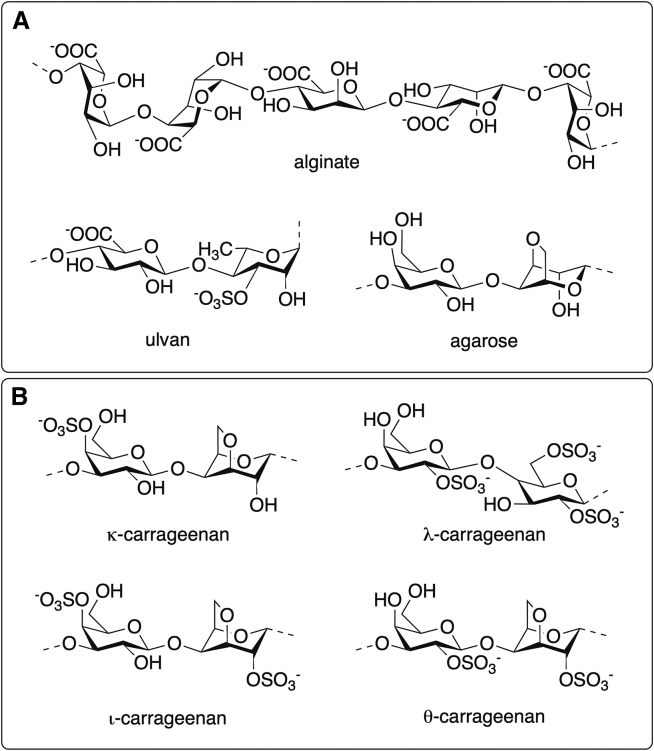
Structures of polysaccharides alginate, ulvan, agarose and carrageenans (A) Structures of the main repeating saccharides of alginic acid, ulvan, agarose and (B) the carrageenans family.

In the realm of red algae, agarans refer to a group of galactans that encompass, among others, agar and agarose.^261^ Besides being employed in the cosmetic industry as gelling agents, agarans have been described to have antioxidant and anti-inflammatory properties.^184^ On the other hand, ulvans are a group of sulfated xylorhamnoglycuronans (polyholosides) found in green seaweeds.^262^ They have the potential to be used as gelling and antiaging agents,^185^ in creams^186^ and as replacement for plastic microbeads in rinse-off cosmetics^263^ (Figure 1A).

##### Carrageenans and carrageenan oligosaccharides

Carrageenans (Figure 1B) are a family of high molecular weight sulphated polysaccharides obtained from some red seaweed species, such as *Eucheuma*, *Gigartina*, *Chondrus*, and *Hypnea*. Structurally, they are composed of alternate units of D-Gal and 3,6-anhydro-galactose (3,6-AG) connected by α-1,3 and β-1,4-glycosidic linkages. Depending on their solubility in potassium chloride and the number and position of sulfate substitutions, as well as the location of the 3,6-anhydro bridge in α-1,4-linked galactose residues, carrageenans are classified into various types, such as λ, κ, ι, ε, and μ; all containing 15 to 40% sulfate groups.^264^^,^^265^ Carrageenans present low solubility, high viscosity and they cannot be assimilated by humans, but they are used as emulsifiers, stabilizers, thickeners, gelling agents in toothpaste preparations, sunscreens, facial creams, antiaging creams, and soaps due to their thickening and water-binding properties.^187^^,^^188^^,^^189^^,^^190^^,^^191^ In addition, carrageenans are incorporated in cough medicines, lotions, shaving creams, shampoos, hair conditioners, and deodorants.^53^

Carrageenan oligosaccharides (CO) are degradation products of carrageenans, exhibiting higher solubility and bioavailability than their polysaccharide counterparts. CO can be obtained from the hydrolysis of natural carrageenans through chemical, physical, or enzymatic processes. The resulting CO have various structures and degrees of polymerization.^266^ The bioactivities of CO are strongly linked to their molecular properties, including the degree of polymerization, molecular size, type and ratio of constituent monosaccharides, as well as the anomeric configuration and position of glycosidic linkages. To the best of our knowledge there are no CO-containing products in the cosmetics market yet, but there is an increasing interest in them due to their antioxidant activity and their well-suited physical properties, coupled with their lower toxicity compared to carrageenans.^267^

##### Chitin, chitosan and its derivatives, and *N*-acetyl-D-glucosamine

Chitin is the second most abundant natural polysaccharide on Earth, after cellulose. In the ocean, chitin is the most prevalent renewable polymer and an important source of carbon and nitrogen for marine organisms. Marine chitin can be obtained from the exoskeleton of crustaceans, such as crabs and shrimps, as well as from foraminifera, porifera, cnidaria, mollusks, and marine gastropods, such as seashells and cone snails.^255^^,^^268^^,^^269^

**Figure 2:**
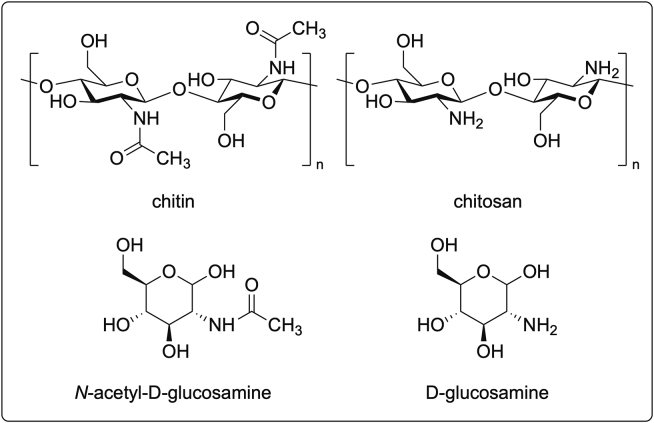
Structures of polysaccharides chitin, chitosan, N-acetyl-D-glucosamine and glucosamine Structures of chitin, chitosan, N-acetyl-D-glucosamine (NAG) and glucosamine.

Structurally, chitin is a linear homopolysaccharide of high molecular weight that is formed by repeated units of *N*-acetyl-D-glucosamine (NAG) linked through β-(1–4)-NAG bonds. Its deacetylation under alkaline conditions forms chitosan. It should be stressed that D-glucosamine and NAG monomers can also be obtained from chitin isolated from crustaceans^270^^,^^271^^,^^272^ (Figure 2). In the cosmetics industry, chitin, chitosan, and their derivatives are used in hair, skin, and oral care products considering the need to address the acceptability of this resource by vegan consumers.^221^

D-glucosamine and NAG are found in the skin (Figure 2), serving as precursors for the biosynthesis of HA and acting as natural moisturizing factors. Furthermore, double-blind clinical trials have proven that oral NAG supplements are a promising treatment against wrinkles, while its topical application improves hyperpigmentation.^192^^,^^193^^,^^194^ As a result, glucosamines are currently incorporated as ingredients in several creams and serums.^195^

Chitin is insoluble in aqueous media which restricts its application and hinders separation from other biomaterials. Chitosan is soluble in acidic aqueous systems, but its poor solubility in water and common organic solvents has restricted its wider use. However, the reactive amino groups in chitosan backbone (Figure 2) enable the chemical conjugation with various biological molecules and hence improve its utilization.^271^

Chitosan and its derivatives are good candidates for skincare due to their positive charge and their high molecular weight, which promote their adhesion to the skin. Chitosan can function as a skin moisturizer, while both chitin and chitosan can be used on various body parts, such as skin, hair, gums, and teeth, and they can already be found in creams, packaging material, lotions, nail enamel, nail lacquers, foundation, eye shadow, lipstick, cleansing materials, and bath agents.^270^ According to the CosIng database, chitin has abrasive and bulking functions, while chitosan is involved in film forming and hair fixing.^273^

With regard to cosmetic applications, chitosan has been incorporated in toothpaste and mouthwashes to reduce *Streptococcus mutans* colonies in early childhood caries.^274^ Another use of chitosan in dentistry is related to the prevention of dental abrasion. Chitodent, a chitosan-based ingredient of toothpaste, is already on market.^271^ Chitosan and its derivatives have been included in a large variety of hair products, such as shampoos, rinses, permanent wave agents, hair colorants, styling lotions, hair sprays, and hair tonics.^270^ Moreover, chitosan is a good polymer matrix and has been included into different formulations such as gels as well as micro and nanoparticles for the delivery of active ingredients.^275^

Recently, chitosan has been found to reduce basal and α-MSH-stimulated melanogenesis in B16F10 melanoma cells. This effect is probably mediated through the suppression of melanogenic-related proteins and tyrosinase activity. Considering that chitosan exhibits no cytotoxicity on melanocytes or keratinocytes, it represents a promising anti-pigmentation agent.^276^

### Fatty acids

The importance of fatty acids has been shown in soft tissue repair and skin nourishment through the stimulation of collagen production, as well as their anti-inflammatory and wound-healing properties, hence they are amenable to be used in creams, emulsions, cosmetic masks, lipsticks, bath fluids, nail polishes, etc.^196^^,^^197^^,^^198^^,^^199^^,^^200^ Moreover, fatty acids play a role in preventing excessive skin dryness and are, therefore, useful in skin and hair treatments.

Emollients are the softening and smoothing substances that shield the skin from water loss. Fatty acids and other lipophilic compounds can function in this capacity as well.^183^ Fatty acid esters are common ingredients in cosmetic formulations as natural emollients and emulsifiers.^277^ Although many fatty acid esters currently used in cosmetics are obtained from higher plants, some bacteria can also produce them. For example, ethyl oleate, which is widely used in many cosmetic products as emollient and perfume, was also obtained from the actinomycete *Nocardiopsis dassonvillei*, which is a symbiont of the marine sponge *Dendrilla nigra*. This compound also displayed anti-inflammatory activity.^278^ Therefore, ethyl oleate could be a potential multifunctional cosmeceutical for skincare products produced in a sustainable manner.^225^^,^^226^

The presence of omega-3 and omega-6 PUFA is responsible for skin nutrition and for maintaining skin health. In addition, they have anti-inflammatory, antiallergic and antioxidant activities.^180^^,^^183^^,^^201^ Interestingly however, PUFA derived from algae are thought to be odoriferous compounds with a fishy, rancid or cucumber scent.^279^^,^^280^ For instance, the aroma of cucumber, generated by *Synura* is linked to 2,6-nonadienal.^281^ Numerous odorant PUFA derivatives can be produced by filamentous cyanobacteria (*Calothrix*, *Plectonema*, *Phormidium* spp., and *Rivularia* spp.) and diatoms (*Asterionella formosa*, *Achnanthes minutissima*, *Amphora pediculus*, *Cymbella minuta*, and *Gomphonema angustum*).^202^^,^^203^ *Cladophora glomerata*, a filamentous green alga found both in marine and freshwater environments,^204^ contains saturated fatty acids (palmitic acid C16:0) and unsaturated fatty acids C16:1 (*n*-7) and C18:1 (*n*-3)^205^ that also act as emollients. Another promising source of PUFA are traustochytrids. The production of PUFA, such as DHA, EPA, and docosapentaenoic acid (DPA), has been explored due to their high production per unit of biomass.^206^^,^^207^ In particular, species belonging to genera *Schizochytrium*, *Aurantiochytrium*, and *Ulkenia* from the Thraustochytriaceae family, are efficient producers of DHA.^208^

### Amino acids, peptides and proteins

Amino acids are natural moisturizers that prevent water loss in the skin.^282^ Algae (macro and micro) and bacteria are good sources of proteins and amino acids, e.g., glycine, alanine, valine, leucine, proline, arginine, serine, histidine, tyrosine, and some MAAs.^49^^,^^209^^,^^210^

Fish-derived proteins and peptides have been investigated for their capacity to protect the skin against UV radiation.^283^^,^^284^ Marine fish proteins mainly consist of collagen, which has been widely utilized in cosmetics for its moisturizing properties.^49^ Sponge-derived, fish skin and jellyfish (*Rhopilema esculentum*) collagen and collagen hydrolysate have been shown to also have wound healing capacity and can effectively protect against the harmful effects of UV radiation, particularly on the antioxidant system. They prevent photoaging by stimulating collagen formation as well as increasing the water content of the *stratum corneum* and defending against the degradation of skin lipids.^142^^,^^222^^,^^285^^,^^286^ Sea cucumbers have also been reported to have high amounts of collagen and mucopolysaccharides that are relatively safe when compared with other sources of animal collagen.^287^^,^^288^ The total protein of the body wall of sea cucumbers contains approximately 70% of insoluble collagen fibers, which can be converted into gelatin after hydrolysis.

Beneficial effects of marine-derived ingredients in skin features can also be observed following their oral intake. This was shown when testing (hydrolyzed) collagen on repairing skin damage, improving skin elasticity and in sun-exposed areas as well as skin hydration, sebum secretion and skin pH.^289^^,^^290^^,^^291^^,^^292^ Marine collagen hydrolysates can also be used in topical applications due to their lower molecular weight.^215^^,^^216^ Besides being used as a source of collagen, marine-derived cosmetics can be used to promote collagen synthesis. For example, the mixture of extracellular glycoproteins and other glucidic exopolymers produced by *Pseudoalteromonas* sp. is commercialized by Lipotec under the name of SeaCode due its ability to enhance the *in vitro* synthesis of collagen type I dermal proteins.^71^

Numerous proteins can have antimicrobial properties.^293^^,^^294^ *Gracilaria dendroides* was the most effective marine alga tested against a battery of bacteria, i.e., *E*. *coli*, *Pseudomonas aeruginosa*, *S. aureus*, and *Enterococcus faecalis*.

Phycobiliproteins, extracted from red algae, have antioxidant, antiaging and anti-inflammatory, activities. They are frequently used in cosmetics (e.g., makeup, skin care, etc.).^220^ R-phycoerythrin is employed primarily in the field of immunodiagnostics,^295^ though it can also be used as a colorant in cosmetic formulations.^221^ Microalgae are known to produce bioactive peptides with various beneficial properties that make them interesting for cosmetics. Besides the glycoproteins mentioned above, certain peptides derived from microalgae or higher taxa, such as starfish, can also stimulate collagen synthesis, which is crucial for maintaining skin elasticity and reducing the appearance of wrinkles.^222^^,^^223^ They can also have antioxidant and anti-inflammatory activity.^296^ Overall, several products are being developed using innovative bioactive peptides, but currently only a few are commercialized from marine sources.^50^

#### Mycosporine-like amino acids (MAAs)

**Figure 3:**
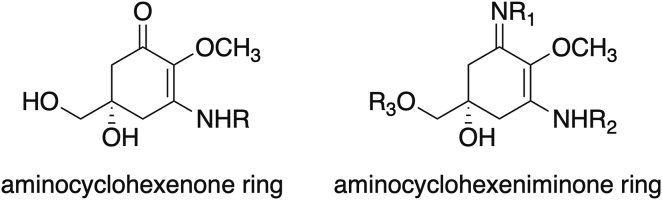
Core structures of cyclohexenone and cyclohexeniminone mycosporine-like amino acids (MAAs) with cosmetic properties as photoprotector, antiaging, and antiwrinkle agents

MAAs are colorless water-soluble, low-molecular-weight compounds that contain either an aminocyclohexenone or an aminocyclohexeniminone ring (Figure 3). Aminocyclohexenone derivatives contain cyclohexenone conjugated with an amino acid, such as mycosporine-glycine or mycosporine-taurine, among others. Instead, aminocyclohexeniminone derivatives possess a cyclohexeniminone conjugated with a glycine or a methylamine attached to the third carbon atom, and an amino acid, amino alcohol, or enaminone chromophore to the first carbon atom (Figure 3). Glycosidic bonds or sulfate esters may also be present within the imine group. Under environmental conditions, MAAs are highly stable molecules.^211^

MAAs absorb UV light and play a role as photoprotectors.^297^^,^^298^ They have a role in antioxidant, antiaging, and anti-inflammatory activities by ROS scavenging potential.^299^ MAAs absorb UV energy which is then dissipated as heat. Depending on the type of ring and substituents, the maximum MAA absorption range lies between 268 nm and 362 nm. In addition, MAAs present high molar extinction coefficients (ε = 28,100 to 50,000 M^−1^ cm^−1^). These two characteristics make them the strongest known natural UVA-absorbing metabolites, and they are also effective against UVB.^297^^,^^300^ MAAs are widely distributed in marine organisms, such as fish, shellfish, crustaceans, corals, sea cucumbers, marine heterotrophic bacteria, fungi and algae.^301^^,^^302^^,^^303^^,^^304^^,^^305^ Moreover, due to their UV absorption capabilities they are regarded as a natural sunscreen. MAAs are not found in higher plants or higher vertebrates, for which the protection against UV radiation is provided by flavonoids and melanin, respectively.^306^

MAAs have been studied, not only as natural alternatives to synthetic sunscreens, but also as antiaging, anti-inflammatory, and antioxidant products.^158^^,^^307^^,^^308^ The epidermal tissue of sea cucumbers contains various amounts of MAAs, such as porphyra-334.^309^ Sunscreen formulations containing liposomes of porphyra-334 obtained from sea cucumbers were found to reduce skin lipid oxidation and skin aging parameters, such as decreased elasticity, wrinkle depth, and roughness.^310^

Currently, there is a large number of patents in international databases describing the production, isolation, and cosmetic application of MAAs, but currently few of them are available on the market.^211^ Some examples are: Helioguard 365, which contains porphyra-334 peptides and shinorine from the red seaweed *Porphyra umbilicalis*, and Helionori (Mibelle AG Biochemistry), marketed by Gelyma.^212^ Finally, Seppic commercializes ASPAR’AGE, an *Asparagopsis armata* MAAs-containing extract that is included in lotions with antiaging properties.^213^^,^^214^

#### DNA repair enzymes: Photolyases

UV irradiation causes DNA alterations by producing photodamage and photoaging.^311^ DNA damage in cells occurs by the formation of cyclobutane pyrimidine dimer (CPD) and pyrimidine 6-4 pyrimidone (6-4PP) photoproducts. Based on the class of photoproducts they repair, there are two different kinds of photolyases: CPD photolyase and (6–4) photolyase. These enzymes utilize energy from blue light to repair damaged DNA by catalyzing a reaction that transfers electrons leading to a reduction of the photoproducts.^312^^,^^313^ Although humans are not capable of producing endogenous photolyases, exogenously manufactured CPD-photolyase preparations have proven effectiveness in reducing the number of CPDs identified after UV irradiation.^314^

The marine cyanobacterium *Synechococcus leopoliensis* (previously known as *Anacystis nidulans*) is the main source of CPD photolyase. A protein extract of *S. leopoliensis* containing CPD photolyase is encapsulated in the liposomes of commercial lotions or creams.^217^ CPD photolyases used in cosmetic products are usually listed on the label as Photosomes or Plankton Extract. There are numerous consumer products that contain CPD photolyases, mainly sunscreens, but other cosmetic preparations contain them as well.^218^^,^^219^

### Pigments and pigment-protein complexes

**Figure 4:**
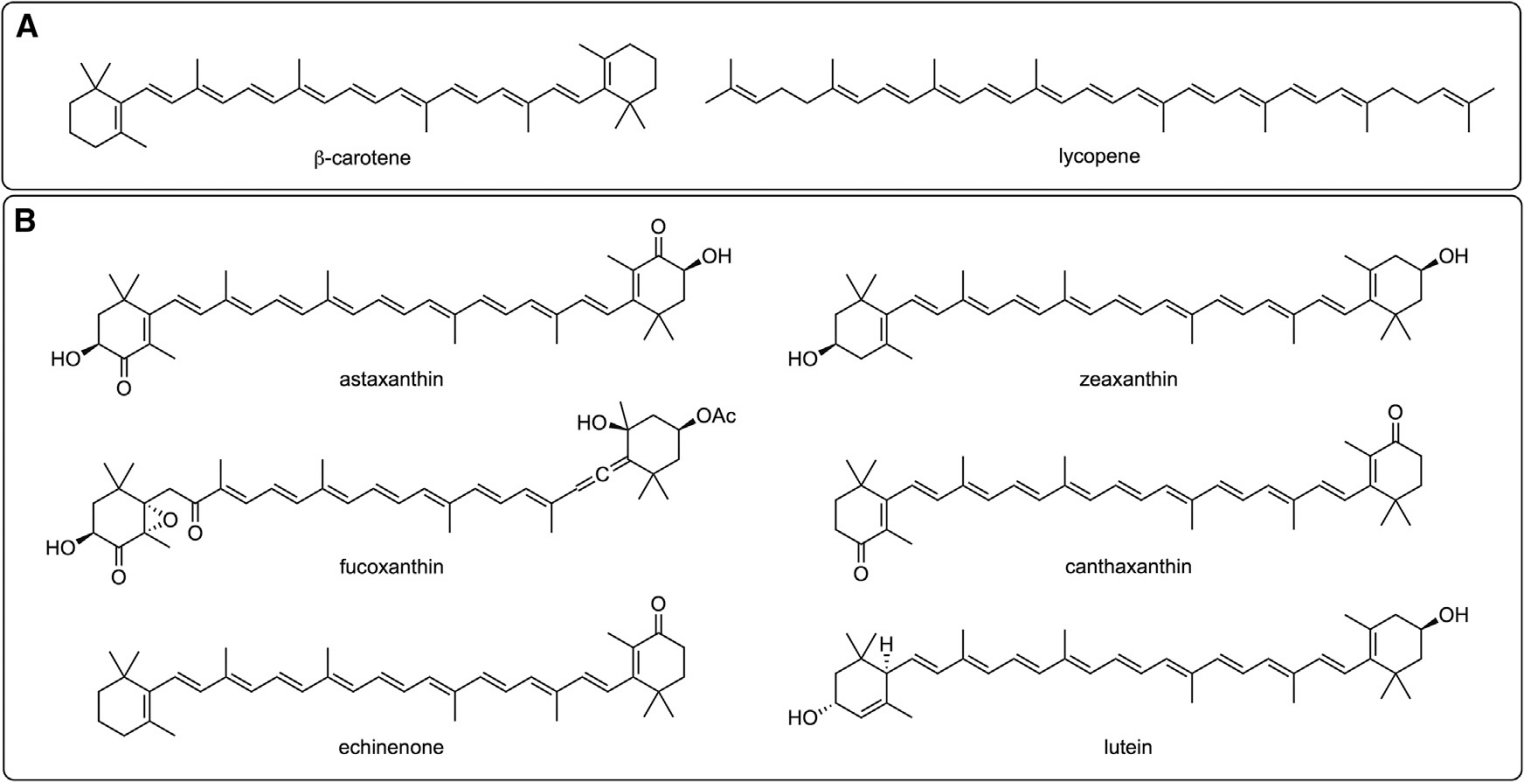
Structures of selected carotenes and xanthophylls (A) Carotenes; and (B) Xanthophylls.

A wide variety of pigments are found in photosynthetic organisms; they accomplish two main roles: collect light from photosynthesis and protect cells from damaging UV rays.^315^ Algal pigments (Figure 4) can be green (chlorophylls), brown (carotenoids - including carotenes and xanthophylls, e.g., beta carotene, astaxanthin, canthaxanthin, lutein, lycopene, and fucoxanthin), or red (phycobilins, e.g., phycocyanin and phycoerythrin).^41^^,^^316^^,^^317^

Carotenoids have been reported to contain antioxidant, anti-inflammatory, and photoprotective properties.^58^^,^^227^^,^^228^^,^^229^ Due to their anti-microbial properties, carotenoids have interesting potential for their use as preservatives by delaying microbial contamination in cosmetics that cause product deterioration.^58^ Many marine sponge-derived actinomycetes, mostly of genus *Streptomyces*, have been investigated as renewable sources of carotenoids for biotechnological products, such as food- and cosmetic-grade natural pigments.^225^^,^^226^ Thraustochytrids, such as *Thraustochytriidae* sp., *Ulkenia* sp., and *Aurantiochytrium* sp., also produce carotenoids. These include beta carotene, astaxanthin, canthaxanthin, zeaxanthin, phoenicoxanthin, and echinenone, which could be used as photoprotective and antioxidant ingredients in different cosmetic formulations.^230^ Cyanobacteria are an excellent source of carotenoids, the second largest family of cosmetic-useful colors, while algae are the primary supplier of cosmetic pigments.^55^ Astaxanthin and fucoxanthin exhibit antioxidant, anti-inflammatory, and photoprotective properties.^95^^,^^103^^,^^105^^,^^231^^,^^232^^,^^233^^,^^234^ An interesting approach for astaxanthin application was shown through its concurrent oral and topical administration, where significant visual improvements in the appearance of skin wrinkles, elasticity, age spots and increased cutaneous hydration were observed.^318^ Marine bacteria belonging to the genus *Paracoccus* such as *Paracoccus* sp. strain N81106, *Agrobacterium aurantiacum*^83^ and *Paracoccus haeundaensis* BC74171T^84^ are promising producers of astaxanthin. The microalga *Haematococcus pluvialis* (Chlorophyceae) and the fungus *Phaffia rhodozyma*, a Xanthophyllomyces, are other natural astaxanthin sources of promising potential.^319^^,^^320^^,^^321^

The tyrosinase inhibitory properties of fucoxanthin aid to diminish and regulate skin pigmentation. Fucoxanthin also has anti-inflammatory properties and contributes to slowing down the aging process of the skin by promoting collagen synthesis.^235^ Other benefits of fucoxanthin include antitumor, antioxidant, antiobesity, and antiangiogenic activities.^233^^,^^322^^,^^323^ *Isochrysis* spp., *Postelsia* *palmaeformis*, *Laminaria digitata, Laminaria japonica*, and other species of brown algae are among those utilized in cosmetics.

Haloarchaea produce C_50_ carotenoids such as bacterioruberin that are of exceptional biotechnological interest. *Haloferax mediterranei* has long been known as a promising candidate for carotenoid production due to its fast growth and ability to utilize different carbon sources.^68^ Its potential as a cell factory for the production of C_50_ has recently been explored.^324^ Carotenoids with high antioxidant capacity, i.e., bacterioruberin, bisanhydrobacterioruberin, trisanhydrobacterioruberin, and their derivates, have been identified in other halophilic archaea, such as *Halococcus morrhuae* and *Halobacterium salinarum*.^67^

In addition to applications in cosmetics, zeaxanthin has significant potential for use in pharmaceuticals as it prevents age-related macular degeneration.^325^ A number of marine bacterial isolates belonging to the bacterioplankton Flavobacteriaceae family (phylum Bacteriodetes) are already known to synthesize xanthophyll carotenoids, such as astaxanthin and zeaxanthin (Table 1). Thus, *Mesoflavibacter zeaxanthinifaciens*,^78^ *Zeaxanthinibacter enoshimensis*,^78^ *Muricauda lutaonensis*,^79^ *Siansivirga zeaxanthinifaciens*,^80^ and *Aquibacter zeaxanthinifaciens*^81^^,^^82^ have been well characterized for zeaxanthin biosynthesis potential. Carotenoids all-trans-zeaxanthin, zeaxanthin monoglucoside, thermozeaxanthins and thermobiszeaxanthins have been isolated from the theromophilic bacterium *Thermus filiformis* (first isolated in 1987 from a hot spring in New Zealand).^67^ Two rare monocyclic carotenoids, (3R, 2′S)-myxol and (3R)-saproxanthin, were found in a new bacterium species from the Flavobacteriaceae family isolated in Okinawa, Japan. Compared to zeaxanthin and beta carotene, saproxanthin and myxol showed stronger antioxidant activity.^67^^,^^326^

Phycocyanin is a blue pigment-protein complex found primarily in cyanobacteria, such as *Arthrospira platensis* (*Spirulina*) and *Synechococcus* sp., but also in the red alga *Galdieria sulphuraria*.^327^ Phycoerythrin is a red protein-pigment complex found primarily in red algae (Rhodophyta) and cyanobacteria. Both have strong antioxidant properties, anti-inflammatory effects and might offer photoprotection to the skin.^236^^,^^237^

**Figure 5:**
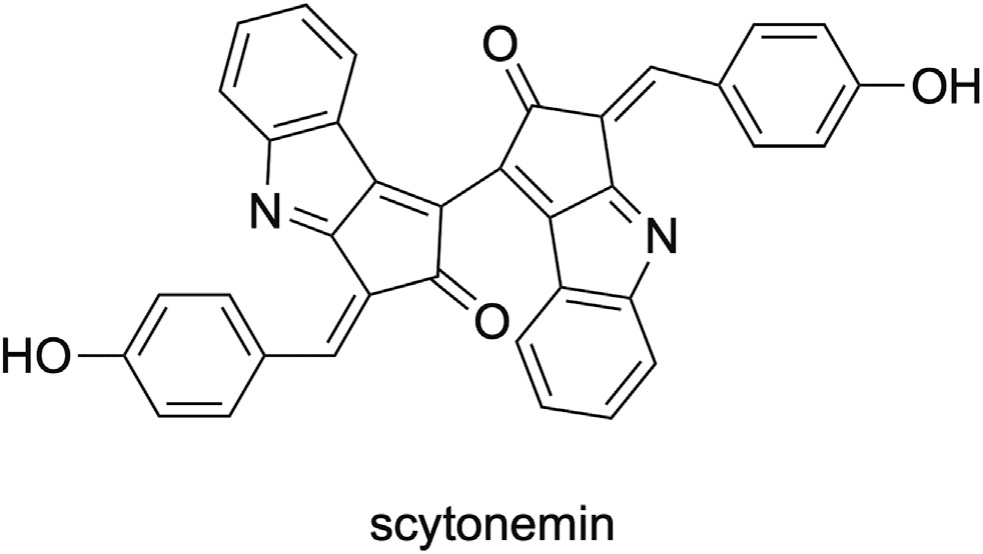
Structure of the pigment scytonemin used for their properties to absorb UV radiation

The cyanobacterial sunscreen pigment scytonemin (Figure 5) absorbs UVA/UVB radiation more efficiently than a commercial formulation.^238^ Scytonemin is produced by several cyanobacteria, such as a *Nostoc* sp. and a *Calothrix* sp. living in crustacean, and *Chlorogloeopsis* sp.^239^

#### Melanin

**Figure 6:**
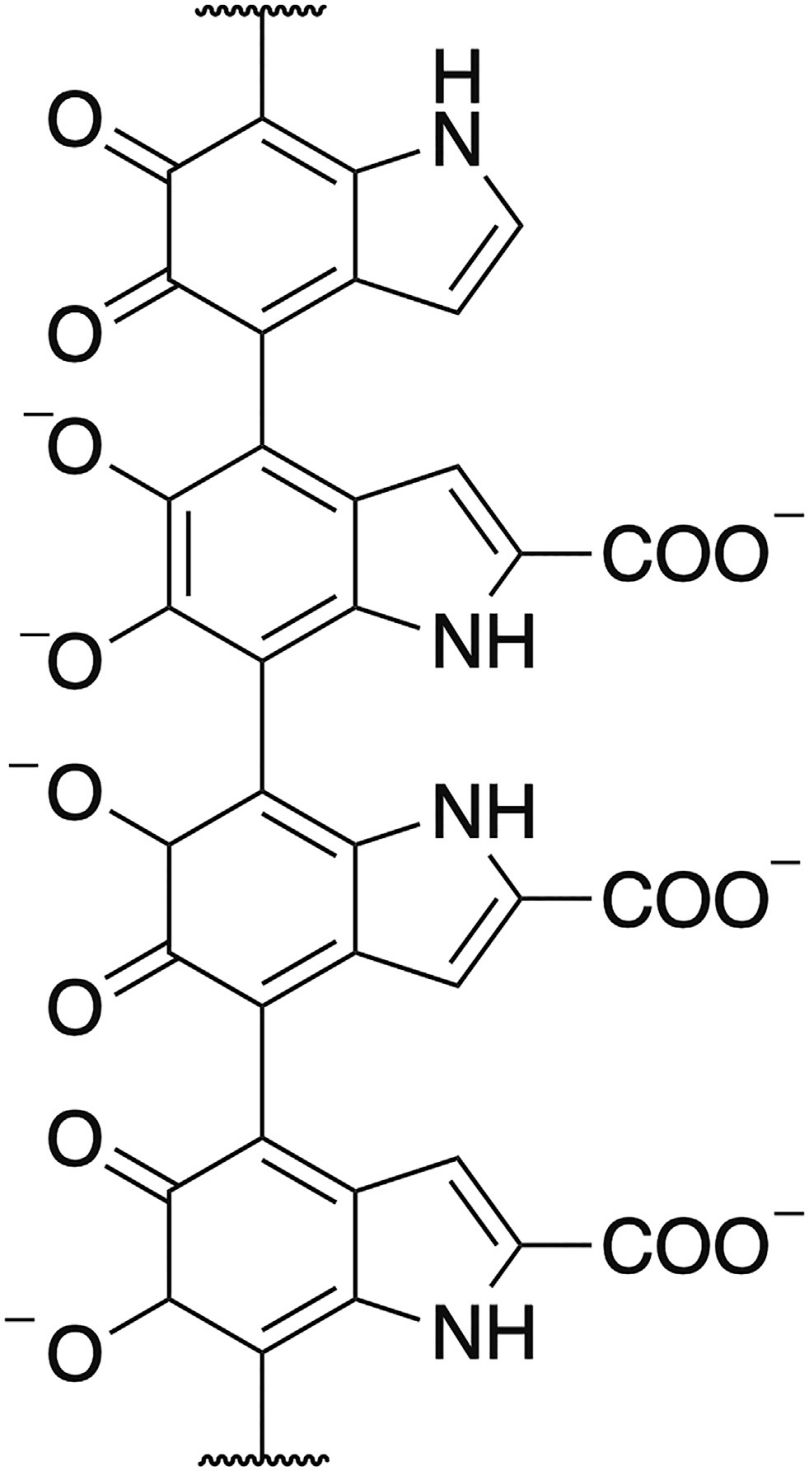
Basic structural unit of melanin. Melanins are used in cosmetics as antioxidant, anti-inflammatory and photoprotective agents

Melanin is the term for a complex group of pigments produced by organisms throughout all domains of life. Its principal function is the protection from burn injuries caused by solar radiation, pigmentation, radical scavenging and as a defense mechanism against predators. Natural melanin is produced by a process called melanogenesis, which starts with the oxidation of tyrosine to L-DOPA mediated by tyrosinase. Although their overall structures are not known, most melanins appear to be a mixture of indole-based polymers, but also contain variable amounts of other pre-indolic products. The basic structural unit of melanin is usually represented by covalently linked indoles (Figure 6).^137^^,^^328^ There are five basic types of melanin: eumelanin, pheomelanin, neuromelanin, allomelanin, and pyomelanin. Of these, eumelanin and pheomelanin are the most abundant in nature.^329^^,^^330^^,^^331^

Currently, marine microorganisms are considered a sustainable source of melanins with potential applications in cosmetic industry.^332^ Due to its photoprotective and antioxidant properties, melanin from squid ink is commercialized as a cosmetic ingredient, Creanatural Sepia Melanin^245^ and used for skin care, hair care, and sun care applications. In recent years, a number of publications have shown the potential of marine microbial melanin in this respect, for example: (i) Melanin produced by *Pseudomonas stutzeri* increases the sun protection factor of commercial sunscreens.^241^ (ii) Melanin produced by the bacterium *Halomonas venusta*, isolated from the sponge *Callyspongia* sp., was formulated with a seaweed concentrate in a cosmetic cream to improve its antioxidant and wound-healing properties.^242^ (iii) Melanin precursors extracted from the marine fungus *Aspergillus nidulans* conferred protection against UVB irradiation and a significant reduction in ROS generated by exposure to direct sunlight.^243^ (iv) Melanin produced by a strain of *Providencia rettgeri* showed anti-inflammatory and SPF enhancement properties.^244^

### Phenolic compounds

**Figure 7:**
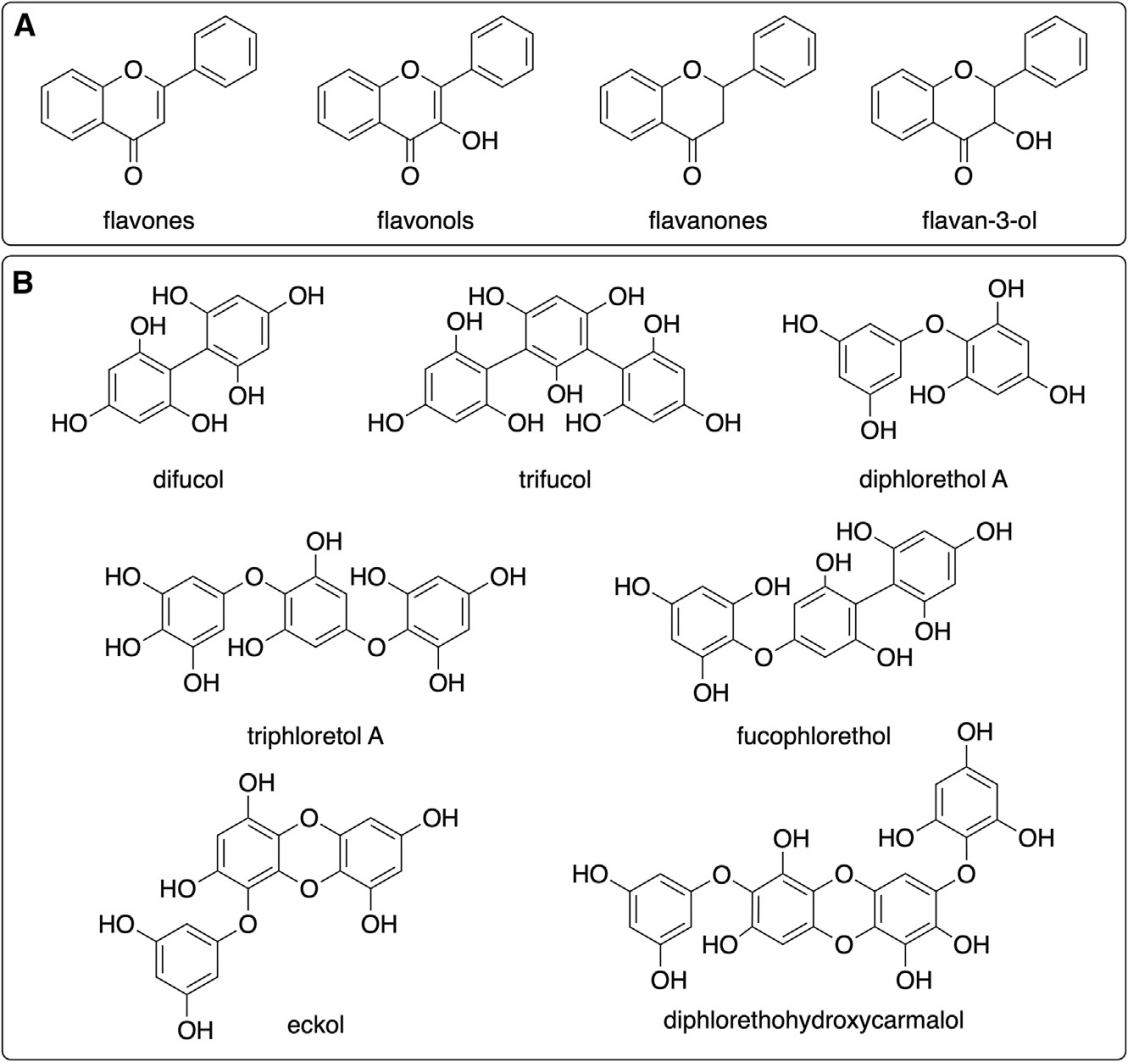
Structure of selected phenolic compounds (A) Main classes of flavonoids found in algae. (B) Examples of different structural classes of phlorotannins.

Phenolic compounds are secondary metabolites of great importance in skin cosmetics.^53^ They can be divided into simple phenolic compounds and polyphenols, which include flavonoids, phenolic terpenoids, and bromophenols^213^ (Figure 7). Several brown (*Ecklonia cava*, *Eisenia arborea*, *Ecklonia stolonifera*, and *Eisenia bicyclis*) and red (*Schizymenia dubyi*, *Wilsonosiphonia howei*, *Rhodomela confervoides*, *Laurencia pacifica*, and *Laurencia rigida*) algae have been shown to contain high levels of bioactive phenolic compounds.^333^^,^^334^^,^^335^ In addition, the polyphenolic content shown by marine dissolved organic matter (DOM) makes it a potential source of compounds for cosmeceutical applications.^336^^,^^337^

Polyphenols have been linked to skin soothing, age spot elimination, antiaging, and UV protection.^39^ They have also been demonstrated to have antimicrobial properties, e.g., rutin, quercetin, and kaempferol.^55^ The red seaweed *Gracilaria dendroides* has shown the highest concentration of these three flavonoids which were then associated to the inhibition of *E*. *coli*, *P*. *aeruginosa*, *S*. *aureus*, and *Enterococcus faecalis*.^338^ Antimicrobial activity against *S*. *aureus*, *Staphylococcus epidermidis* and *Trichophyton rubrum* was also proved in DOM.^337^ Marine brown seaweeds are the only organisms on Earth that produce phlorotannins.^213^ These are polyphenols with considerable biological activity and play a vital role in the production of bioactive substances.^335^ Phlorotannins are useful in cosmetics for a variety of reasons, including its ability to inhibit matrix metalloproteinase (MMP), act against the bacterium causing acne, *Propionibacterium acne*, act as an antioxidant, and to reduce inflammation and allergies.^213^^,^^339^^,^^340^ Phlorotannins from brown algae exhibited anti-inflammatory effects on mouse ear edema and are considered potent inhibitors of proinflammatory cytokines, such as nitric oxide synthase (iNOS), cyclooxygenase-2 (COX-2), tumor necrosis factor alpha (TNF-α), and interleukin-1 beta (IL-1β) and 6 (IL-6).^341^ Some brown macroalgae such as *Turbinaria ornata* and *Padina boergesenii*, can be a source of polyphenols with antioxidant and high tyrosinase-inhibiting activities, demonstrating their potential for antiaging, UV shielding and skin whitening formulations.^342^^,^^343^

Phenol extracts from seaweed are already present on market: as natural UV screening (extracted from *Porphyra umbilicalis*, produced by AETHIC in UK), or an antiaging agent (ECKLEXT BG, produced by NOF Group, product from of *Ecklonia kurome*, harvested in Japan).^213^

### Sterols

**Figure 8:**
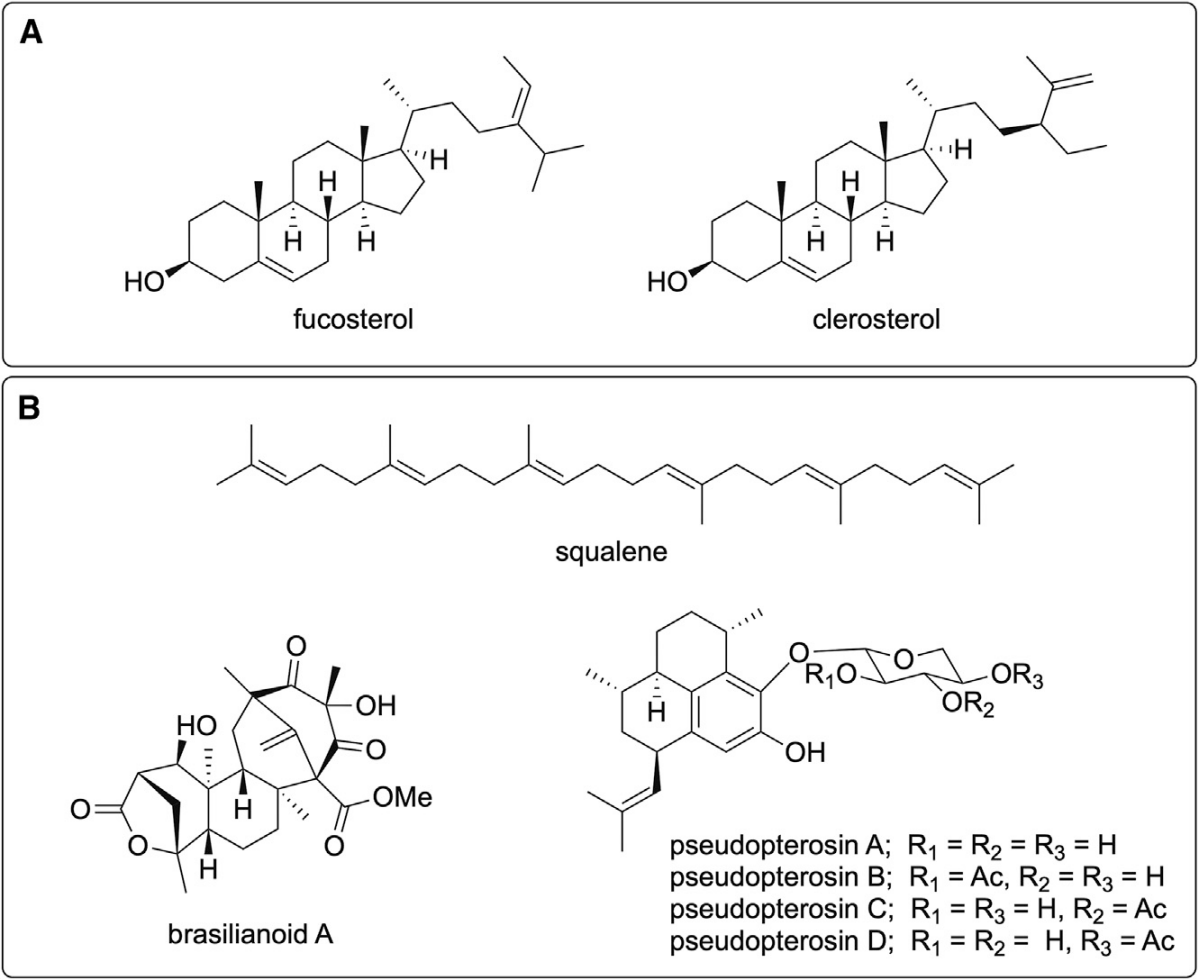
Structure of selected steroids and terpenoids Structure of selected steroids (A) and terpenoids (B) with cosmetical properties.

Marine sterols are naturally present in plants, animals, and fungi. Among marine organisms, algae contain phytosterols with significant pharmacological benefits.^247^ Nonetheless, the use of marine sterols in cosmetics is scarce, with fucosterol as the main representative example (Figure 8A). Fucosterol is the major phytosterol obtained from brown algae which has demonstrated to possess anti-inflammatory and antioxidant properties.^344^^,^^345^^,^^346^ It is a promising natural antiaging agent to protect against skin photodamage.^345^ The cytoprotective effects of fucosterol also suggest its use for the treatment of dermal injuries induced by hypoxia^346^ or to reduce the inflammatory responses induced by air pollutants, suggesting its application for formulating skincare products in rejuvenating cosmeceuticals.^347^

Another example is clerosterol, a double bond isomer of fucosterol isolated from the green alga *Codium fragile*, which has showed anti-inflammatory and antioxidant properties, suggesting an effective therapeutic potential against UVB-induced inflammatory and oxidative skin damages.^248^

### Terpenoids

Scientific attention has increasingly been focusing on squalene, an active terpenoid found in both animals and plants (Figure 8B). Squalene is a natural triterpene which is an intermediate in sterol biosynthesis and has antioxidant potential.^348^ In humans, squalene constitutes approximately 12% of sebum and offers various beneficial effects, including anti-inflammatory, detoxifying, moisturizing, and antioxidant activities. As both squalene and sebum decline with age, there is a growing cosmetic demand for squalene supplementation. Squalene was originally obtained from shark liver oil; however, it can also be sourced from microorganisms. Squalene plays an important role in topical skin lubrication and cellular structure and protection; thus, it is used in cosmetics to keep skin moisturized. Moisturizing creams containing squalene are non-toxic, non-irritating, and non-sensitizing, while providing antistatic properties.^249^ Both squalene and hydrogenated squalene are used in cosmetics. Squalene can also be added to moisturizers as an emollient that is quickly absorbed through the skin.^250^ Thraustochytrids are some of the major squalene-producing organisms.^349^^,^^350^ For example, the HS-399 strain of *Aurantiochytrium acetophilum* was isolated from a mangrove swamp in Biscayne Bay, Florida, United States, as a producer of squalene and lipids.^351^

A highly oxygenated diterpene, gagunin D, isolated from the marine sponge *Phorbas* sp., was found to exhibit antimelanogenic activity by suppressing tyrosinase expression, increasing its rate of degradation, inhibiting tyrosinase enzymatic activity and downregulating the expression of proteins associated with melanosome transfer.^352^ Due to its multi-functional properties, gagunin D and its analogs can be considered potential candidates for skin-whitening cosmeceuticals.^352^

Meroterpenoids (named brasilianoids A–F) from marine sponge-associated fungus, *Penicillium brasilianum*, have also shown potential for cosmetic applications.^251^ One of these brasilianoids significantly stimulated the expression of filaggrin, an essential natural moisturizing factor that maintains the ability to regulate the skin’s moisture barrier,^252^ and of caspase-14, which is responsible for controlling transepidermal water loss (TEWL) and for sensitivity to UVB damage.^253^ Thus, this compound is the first example of a natural product that can be used to promote protection against UVB-induced cell damage, suggesting its potential as cosmeceutical for skincare and for the treatment of dermatological diseases.^251^

Sea cucumbers produce saponins, which are usually triterpene glycosides of the holostane type.^254^ Some saponins can decrease dandruff and alleviate psoriasis when applied topically, in addition to ameliorating hyperpigmentation and rosacea, strengthening blood vessels, and improving water penetration.

The tricyclic diterpene glycosides pseudopterosins A–D, are the most notable marine natural products in the cosmetic industry.^41^ They are described in more detail in the cosmeceuticals section.

### Other compounds

**Figure 9:**
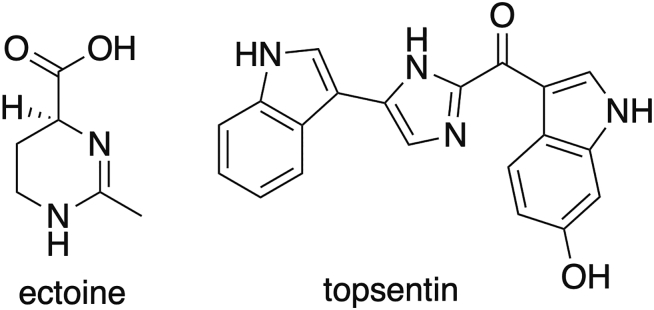
Other metabolites with cosmetical properties

A bacteria-derived compound is ectoine or 1,4,5,6-tetrahydro-2-methyl-4-pyrimidinecarboxylic acid (Figure 9), which is an osmo-protectant produced in response to osmotic stress.^353^ Ectoine was first isolated from *Ectothiorhodospira halochloris* and then later from other halophilic bacteria, such as α- and γ-proteobacteria, and, under high salt concentrations, by some Actinobacteridae.^354^ This compound improves the hydration of the cell surface by increasing intermolecular spacing and boosting the mobility of lipid head groups.^354^ Furthermore, it is well tolerated by humans.^355^^,^^356^^,^^357^ Thus, ectoine is an effective long-term moisturizer that prevents dehydration of the epidermis.^353^^,^^358^ It also reduces skin inflammation and it has been evaluated for the treatment of moderate atopic dermatitis.^355^

An alkaloid isolated from the marine sponge *Spongosorites genitrix*, topsentin, was shown to protect human keratinocytes against UV-induced damage, thus underlining its potential in cosmetic formulations.^128^ Moreover, bioactive indole derivatives isolated from the marine sponges *Rhopaloeides odorabile* and *Hyrtios* sp. showed antioxidant capacity similar to that of Trolox (water-soluble analog of vitamin E).^359^ Host microorganisms are also of potential interest. An example is an *N*-acyl dehydrotyrosine derivative, isolated from *Thalassotalea* sp. bacterial strain PP2-459 living in crustaceans, which can act as a superior tyrosinase inhibitor compared to the commercial products kojic acid and arbutin.^360^

## Cosmeceuticals

Traditionally, cosmetics were viewed simply as products for cleansing, enhancing appearance, or altering looks without affecting the body’s structure or functions. However, there has been a recent emergence of hybrid products that combine cosmetics with medicinal properties through the incorporation of bioactive substances into creams, lotions, and ointments. Notably, there is a growing interest among cosmetics suppliers to integrate extracts from marine organisms together with marine minerals into cosmeceutical products that provide multiple benefits such as antioxidants, UV protection, MMP inhibitory activities, antiaging properties, essential vitamins and minerals. The term cosmeceutical derives from the combination of 'cosmetic' and 'pharmaceutical' to describe cosmetic products containing biologically active ingredients that have either medicinal or drug-like effects.^361^^,^^362^ They are formulated to enhance beneficial physiological effects at the cellular level, in addition to improving skin appearance and having highly effective and stable properties for therapeutic use with low toxicity. As a result of this trend, a new market niche for cosmeceuticals has emerged and continues to grow in popularity.^361^^,^^362^

Cosmeceuticals deriving from marine organisms have drug-like benefits and contain active ingredients such as vitamins, terpenes, saponins, squalenes, carotenoids, flavonoids, phlorotannins, phenolic compounds, mycosporines and MAAs, polysaccharides (e.g., chitin, chitosan, fucoidans, carrageenans and alginate), parabens, fatty acids, peptides, enzymes, and minerals. These have a broad range of bifunctional properties such as antibacterial (e.g., against *Propionibacterium acne*, which causes the common skin disease acne vulgaris^363^), antioxidant, anti-inflammatory,^364^ antiallergic, MMP inhibition, hyaluronidase inhibition, tyrosinase inhibition, photoprotective, whitening, tissue regeneration, wrinkle reduction, and hair growth promotion properties, as well as enhanced physicochemical properties, such as viscosity increasing, thickening, texturizing, emulsifying, gelling or moistening for prolonged and controlled drug release and to develop cohesive matrices. Several marine organisms, including corals, micro- and macroalgae, bacteria, fungi, fish, crustaceans and sea cucumbers, have potential to be used as cosmeceuticals.^47^^,^^92^^,^^365^^,^^366^^,^^367^^,^^368^^,^^369^^,^^370^ In addition, antibacterial, antifungal and antioxidant activities have been recently found in marine DOM^293^^,^^337^ which could be harnessed for products targeting antiaging, skin infections or as preservatives in cosmeceutical formulations. Although further research and technological advancements are needed to unlock the full potential of DOM and to enhance the commercial viability of DOM-based products, the lack of cytotoxicity and its abundance makes it a promising bioresource.^337^

A number of commercially successful examples are already on the market. One of these are the anti-inflammatory and analgesic diterpene-glicosydes pseudopterosins (Figure 8B), discovered in 1986 from the marine sea-whip gorgonian octocoral *P. elisabethae*,^149^^,^^151^^,^^371^^,^^372^^,^^373^^,^^374^ which have the ability to reverse allergic responses in the skin and were successfully marketed as a skin cream named Resilience by Estée Lauder, after acquiring the pseudopterosins patents.^375^^,^^376^ Estée Lauder was able to attain a profitable commercial supply by harvesting branches of these corals. Nevertheless, the long-term harvesting of wild species is considered unsustainable. Consequently, efforts have been made to find symbionts of *P*. *elisabethae* that produce pseudopterosins, and the symbiotic dinoflagellate *Symbiodinium* sp. has been shown to be responsible for the production of these diterpenes.^377^ Work has also been done on the chemical synthesis of these compounds which, due to their multiple-steps, were not economical.^378^ Alternatively, pseudopterosins-type bioactives can be synthesized from their biotechnologically produced precursor 1R-epoxy-elisabetha-5,14-diene.^378^

## Bioassays for cosmetics screening

When screening for novel activities from marine biomass, the goal is to detect and potentially quantify the biotechnological potential.^379^ The bioactivity assays that are most relevant for the cosmetics industry are described below.

### Antioxidant capacity screening

In biological terms, skin antioxidants are compounds able of either interrupting or inhibiting oxidation progression, which occurs under the impact of ROS. Antioxidant entities can act against ROS or their by-products through different mechanisms, including chain-breaking activity, chelation of transition metals and singlet-oxygen scavenging.^16^ In general, antioxidants are classified as lipid-soluble (hydrophobic) or water-soluble (hydrophilic) substances.^380^ In cosmetic care formulations, the most widely used natural hydrophilic antioxidants include compounds from the class of polyphenols (flavonoids, phenolic acids, lignans and stilbenes) and vitamins (e.g., vitamin C). Hydrophobic antioxidants may also contain vitamins (e.g., vitamin E and quercetin), as well as carotenoids (e.g., lycopene, lutein). However, a number of antioxidants act amphiphilically, such as astaxanthin commonly found in marine organisms.^369^^,^^381^^,^^382^^,^^383^ Antioxidant activity by being prophylactic against photoaging and pertinent skin pathologies has been a fundamental property routinely investigated in natural products. A comprehensive overview of the enzyme-free antioxidant assays, including DPPH, ABTS/TEAC, CUPRAC, Folin-Ciocalteu, and ORAC, is provided by Sabotič et al.^379^

### Antiaging capacity screening

#### Anti-elastase assay

Elastin is an insoluble, fibrous protein of the ECM known for its unique elastic recoil properties, and is vital for maintaining skin elasticity and resilience to tissues and organs.^42^^,^^384^ Together with collagen, it forms a fibrous network under the epidermis.^385^ The enzyme elastase, which belongs to the chymotrypsin family of proteases, is the only enzyme capable of degrading elastin.^386^ Elastase is also able to hydrolyze nearly all structural proteins of the connective tissues, such as collagen and fibronectin along with other ECM proteins.^386^^,^^387^ Since elastic fibers are easily decomposed by the elastase secretion caused by UV exposure or ROS, inhibition of elastase activity has been considered a useful approach to protect the skin from premature aging.^9^

#### Anti-collagenase assay

Collagen is the most abundant protein in the ECM of dermal connective tissue where it accounts for 80% of the skin’s dry weight.^15^^,^^388^ It is responsible for maintaining the elasticity, flexibility and strength of the skin.^10^ MMPs are responsible for the degradation or synthesis inhibition of collagenous ECM in connective tissues. The MMPs form a family of structurally and functionally related zinc-containing endopeptidases that are not constitutively expressed in the skin but are induced temporarily in response to exogenous signals, such as UV radiation. Several types of skin cells are capable of producing MMPs, including keratinocytes, fibroblasts, macrophages, endothelial cells, mast cells, eosinophils, and neutrophils. MMPs can act alone or in combination, exhibiting various substrate specificities. Typically, collagen cleavage is initiated by MMP-1 and it is further degraded by other MMPs, such as MMP-3 and MMP-9. Having the ability to cleave type I, III, VII, VIII and X collagen, MMP-1 (fibroblast collagenase) is mainly responsible for collagen breakdown in skin.^388^^,^^389^ The inhibition of the specific enzyme is deemed to delay collagen degradation and subsequently the wrinkling process.^42^

#### Anti-hyaluronidase assay

HA is a high molecular weight glycosaminoglycan made up of repeating units of disaccharides, D-glucuronic acid and NAG.^390^ It plays a vital role in retaining the moisture of the skin, its structure and elasticity, while facilitating the exchange of nutrients and waste products, rapid tissue proliferation, regeneration and repair. This compound also contributes to the structural maintenance of ECM.^10^^,^^42^ Hyaluronidases are the enzymes catalyzing HA degradation, which increases the permeability of connective tissue and decreases viscoelasticity of bodily fluids,^391^ thus potentially facilitating the spread of pathogenic microorganisms and their toxins through the connective tissue.^392^ Therefore, hyaluronidase inhibition is regarded as a key approach in maintaining high HA levels and skin moisture retention, along with treating other diseases related to HA depletion.

#### Anti-tyrosinase assay

Melanin is a dark pigment produced by up to 10% of skin cells in the innermost layer of the epidermis.^393^ It plays a major role in our phenotypic appearance as it determines skin color and pigmentation.^394^ Melanin biosynthesis, or melanogenesis, is a well-known physiological response of the human skin upon exposure to UV light and other stimuli, initiated by the enzyme tyrosinase.^395^^,^^396^ The role of melanin is to protect the skin against UV light damage by absorbing UV sunlight and removing ROS.^397^ However, tyrosinase overactivity leads to the accumulation of melanin in parts of the skin, resulting in more pigmented patches, which is an aesthetic problem particularly prevalent in middle-aged and elderly individuals.^394^^,^^397^

Tyrosinase inhibition has been characterized as an important strategy for blocking melanogenesis.^398^ Kojic acid, arbutin, glycolic acid and azelaic acid are some well-studied tyrosinase inhibitors currently used in cosmetics. Besides the treatment of some dermatological disorders associated with melanin hyperpigmentation, tyrosinase inhibitors have found an important role in the cosmetic industry for their skin-whitening (lightening) effect and depigmentation after sunburn.^393^

#### Oxidative damage estimation by using human fibroblasts

Fibroblasts play a key role in wrinkle formation because they produce basic structural skin substances (i.e., collagen, elastin and HA),^12^ which are responsible for elasticity and hydration. The oxidative stress induced in skin by UVA exposure and ROS generation activates complex cellular processes in fibroblasts, which contribute to dermal aging characterized by wrinkles and loss of elasticity.^399^ Thus, human fibroblasts constitute a vital cellular setting and a real-life model for simulating oxidative damage and assessing the protective role of natural extracts/compounds.

### Stability testing

Stability testing is at the core of cosmetic product development, as it aims to ensure that developed products meet not only the intended physico-chemical quality attributes, closely linked to products functionality and aesthetics when stored under appropriate conditions, but also microbiological quality standards inextricably linked to the safe use of cosmetics. Generally, accelerated tests are performed to predict long-term stability of the product and its shelf life. Tested samples are then re-evaluated for active ingredient content, organoleptic properties, pH, viscosity, phase separation, conductivity, water activity and other relevant features.^400^ Troubleshooting most of the stability concerns of cosmetic formulations typically starts at raw material classes that comprise the skin care and makeup ingredient list. The stability testing of marine cosmetics could be more challenging since their derived excipients have been used only recently and therefore have been tested less than, for example, more commonly used thickeners (e.g., cellulose ethers). For example, the addition of a non-uniform thickener can cause an erratic low viscosity dial reading or formula splitting. On the other hand, applicability of e.g., algae-derived carrageenans and alginate is already well known not only in cosmetics, but also in the food and pharmaceutical industries. With regard to microbiological stability, the main aim of testing is to ensure that the product reaching the market is microbiologically safe and remains safe throughout the product shelf life under normal or reasonably foreseeable usage conditions. Appropriate microbiological quality requires adequate quality of raw materials and compliance with good manufacturing practice (GMP) during manufacturing and packaging (i.e., primary preservation strategy), including the incorporation of preservatives or a self-preservation approach (i.e., secondary preservation strategy). By following the regulatory requirements, final products are subjected to microbiological control with data regarding quantitative levels of microorganisms and the efficacy of antimicrobial preservation included in the Cosmetic product safety report. The natural origin of marine resources is an appreciated feature enabling the formulation of bio-based and safe products. Concomitantly, as marine-derived raw materials could support microbial growth, they require a strict microbiological control and the same attention has to be paid to marine-based final products. In addition, compounds of marine origin are also being studied for their antimicrobial activity. Currently however, they show higher potential to be incorporated as excipients supporting microbiological quality of the product, rather than to be used as main preservatives.^49^^,^^401^^,^^402^

### Safety

A proven efficacy assessment and a comprehensive toxicological assessment have to be determined for the use of cosmetic products and their intended use model.^403^^,^^404^ Based on the product type, a number of safety and toxicological tests are required, such as irritation, corrosion, penetration or sensitization etc., to confirm that they are safe for application. According to the Regulation (EC) No 1223/2009 on cosmetic products, testing on animals is prohibited not only for finished cosmetic products but also for ingredients used in cosmetics products (a non-exhaustive list of these ingredients is provided in Table S1). Since 2013, the ban on animal testing has been valid also for substances considered carcinogenic, mutagenic, or toxic for reproduction. To comply with these requirements, a list of validated cell-based *in vitro* models for predicting the safety and toxicity of cosmetic ingredients was proposed by the European Center for the Validation of Alternative Methods (ECVAM).^405^ Several *in silico* methods have also been developed.^406^

These safety requirements for testing cosmetics products are listed in the “Notes of Guidance for the Testing of Cosmetic Ingredients and their Safety Evaluation”, and include the following parameters: (a) acute toxicity; (b) corrosivity and irritation; (c) skin sensitization; (d) dermal/percutaneous absorption; (e) repeated dose toxicity; (f) reproductive toxicity; (g) mutagenicity/genotoxicity; (h) carcinogenicity; (i) toxicokinetics studies; (j) photo-induced toxicity, and (k) human data (described in the subsequent paragraph).^407^^,^^408^

There are currently four *in vitro/in chemico* OECD Test Guidelines (TGs) available for skin irritation and corrosion testing, in addition to three *in vitro/in chemico* TGs available for evaluating skin sensitization and seven for serious eye damage/irritation testing.^409^^,^^410^

Skin irritation and corrosion testings are performed in human skin equivalent models (e.g., SkinEthic, EpiDerm SCT, SkinEthic RHE), while excised human skin is still used as the gold standard for the evaluation of dermal absorption.^410^^,^^411^^,^^412^ To overcome one of the main drawbacks of skin equivalents, i.e., their lack of a vascular system, next-generation *in vitro* skin models are being developed as 3D skin models integrating immune components or skin-on-a-chip (based on organ-on-a-chip technologies) that can reflect more closely the skin architecture and cell composition.^413^^,^^414^ Complementarily, 3D skin models are also applicable for evaluating the activity of marine extracts *in vitro.*^415^ This is done in addition to TEWL measurement, a sensitive indicator of not only skin irritation (and hence widely used in analyses of irritancy potential), but also of the protective properties of topical products.^403^^,^^416^

For mutagenicity/genotoxicity testing, five *in vitro* OECD TGs are accessible, while for the carcinogenicity, photo-induced toxicity and ADME/TK testing, one *in vitro* OECD TG is available for each parameter.^409^^,^^410^ In all cases, a thorough review of all available data on the tested substance should first be performed to make the optimal testing strategy. Generally, for mutagenicity/genotoxicity testing, the bacterial gene mutation (Ames) test is preferable. When it is not applicable (e.g., for nanomaterials), the mammalian gene mutation test can also be performed to obtain information on mutagenicity at the gene level. In addition, information on the chromosome breakage and/or rearrangements (clastogenicity), as well as numerical chromosome aberrations (aneuploidy), are commonly obtained by the *in vitro* micronucleus test.^410^

Carcinogens are divided into two groups, i.e., genotoxic agents, which disrupt the integrity of the genome by interacting with DNA, and nongenotoxic carcinogens, which exhibit carcinogenic effects through other mechanisms.^417^^,^^418^ For the carcinogenicity assays validated by ECVAM, the Bhas 42 cell line and Syrian hamster embryo cell lines are used. Carcinogenicity estimates can be made for nongenotoxic agents by *in vitro* cell transformation assays, although it is believed that an *in vitro* method alone cannot provide sufficient information.^419^

As a part of photo-induced toxicity testing, photoirritation and photosensitisation can be assessed *in vitro* by a validated 3T3 Neutral Red Uptake Phototoxicity Test. The cytotoxicity of a cosmetic ingredient is compared in the presence and in the absence of exposure to a non-cytotoxic dose of UV/VIS radiation. In the case of a positive cytotoxicity outocome, a reconstructed human epidermis phototoxicity test is performed.^420^^,^^421^

Toxicokinetics is the entire process of absorption, distribution in the body, metabolism, and excretion (ADME) of a toxic substance from the body.^422^ Modeling toxicity behavior *in vitro* is of great importance to establish the potential degree and type of toxicity of a compound in the organism.^423^ On the other hand, no *in vitro/in chemico* OECD TGs are available for acute systemic toxicity and repeated dose toxicity testing. Acute toxicity is the most common conventional test to obtain dose-response information. It is usually performed by administering one or several doses of the compound over a 24-h period and observing general toxic effects.^407^^,^^424^ While data on acute toxicity are not mandatory for assessing the safety of cosmetic ingredients for consumer use according to SCCS/1647/22 Corrigendum 2,^410^ the lack of alternative repeated dose toxicity tests presents a bottleneck for the introduction of new compounds on the EU market. Along these lines, no *in vitro/in chemico* OECD TGs are currently available for reproducive/development toxicity testing. To address these challenges, the cosmetic industry is extensivity working on developing Next Generation Risk Assessment strategies. Recently, there have also been approaches to couple these tests with software for prediction of effects on human health and related toxicities.^417^

For an interested reader, current EU regulatory requirements for the human health assessment of chemicals under Cosmetic Products Regulation and Regulation (EC) No 1907/2006 concerning the Registration, Evaluation, Authorisation and Restriction of Chemicals^425^ are well summarized by F. Pistollato et al. to identify the main challenges in current regulatory testing practice as well as presented in the »Production « chapter and in Document S1.^409^ In addition, a special concern must be given to the possible toxicity of marine-derived excipients linked to the major threat of heavy metal, chemical, and plastic contamination in the marine environment. While extracting marine-derived ingreedients, a thorough purification procedure must therefore be followed.^426^

### Efficacy and tolerability

According to legislation, consumers should be protected from misleading claims concerning efficacy and other characteristics of cosmetic products. Evaluation of their final efficacy to substantiate advertising claims represents a further improvement from *in vitro* (bench) and *in vivo* (animal) assessments, involving testing on subjects within clinical studies. Following a product application regime as proposed within the study protocol, testing involves instrumental assessment of skin features, where obtained data represent a valuable tool for the evaluation of skin features already at a sub-visible level. Additionally, due to complexity of final skin performance and with awareness that differences, even though measurable, do not necessarily reflect meaningful effects, expert grade and/or self-assessments can be performed for product tolerability and acceptance.

Cosmetic claims about the ability of (marine) cosmetics to regenerate the skin barrier, reduce inflammation and skin irritations or help to heal wounds are typically assessed by measuring TEWL and skin color (primary erythema), in addition to skin pH and hydration. TEWL is a good indicator of the integrity of the skin barrier function, capable of detecting even subvisible skin barrier changes in acclimatized volunteers under suitable measuring conditions. According to the definition, it equals to a passive loss of water that passes through intact epidermal layer of the skin by diffusion processes and is assessed by measuring the water vapor flux density just above the skin surface by suitable measuring devices, like open or closed chamber devices. Healthy skin barrier corresponds to TEWL values below 14 g/m^2^/h, while values above 20 g/m^2^/h indicate an impaired skin barrier.^416^^,^^427^ Skin pH is another parameter for indicating skin barrier impairment before it is visible by eye. It is measured as the pH value of an aqueous solution on the skin surface by a flat glass pH electrode.^428^ In healthy skin its surface pH is on average around 5 or below, while increased pH values are observed in skin with an impaired barrier or irritated skin.^429^ Both TEWL and skin pH measurements prior and after application of (marine) cosmetic products are also used to support product claims. Beneficial effects of marine cosmetics on irritated or inflamed skin can further be supported by the decrease in skin redness, measured as skin erythema index or a∗ (redness) and L∗ (darkness) values by (narrow band) reflectance spectroscopy or skin colorimetry, respectively.^430^^,^^431^ Erythema and skin darkening are also increased by solar radiation, as skin color predominantly depends on pigments, i.e., hemoglobin (red) and melanin (brown-black eumelanin or reddish yellow pheomelanin). Therefore, skin color measurements are also applicable for evaluating skin protection against UV radiation. While the minimal erythema dose is defined as the UV dose that produces perceptible erythema or erythema with defined boundaries on an individual’s skin, persistent pigment darkening is a widely used *in vivo* method for measurement of UVA protection factor.^432^^,^^433^^,^^434^ Moreover, skin whitening effects of algae extracts used to improve the uneven skin tone and hyperpigmentation of (photo)aging skin can also be supported by measuring the skin color, in particularly L∗ value and the individual typology angle, with higher values of both that are typical for lighter skin color. Oher clinical signs of photoaging include rough skin and dryness, wrinkles, deep furrows, loss of skin elasticity, telangiectasias, solar elastosis, in most severe cases also precancerous lesions and skin cancer.^435^ The antiaging effects of (marine) cosmetics are commonly based on the increased hydration and improved biomechanical properties of human skin through enhancing the biosynthesis of collagen and elastin, with elasticity and viscoelasticity strongly influencing its protective function.^221^ Regarding moisturizing effects, Corneometer CM 825 is recognized as the gold standard for accurately assessing the hydration level of the upper parts of *stratum corneum* by measuring the electrical capacity as the alternating voltage of *stratum corneum*.^323^^,^^436^^,^^437^ On the other hand, skin replica technique is used for the evaluation of skin wrinkle parameters^438^ in addition to other novel non-contact techniques as, for example, optical 3D *in vivo* skin imaging.^439^ To monitor and measure biomechanical properties of the skin more in detail, Cutometer or DermaLab Combo as commercially available non-invasive (suction) skin elasticity meters have been frequently utilized.^440^^,^^441^ Such devices are suitable to support not only antiaging, but also anticellulite claims applicable to marine cosmetics.^442^ To support the latter, ultrasound imaging confirming a decline in the thickness of the thigh subcutaneous adipose tissue, and microcirculation measurement showing blood flow improvement in the affected areas, for example by Laser Doppler Imaging, are also used.^442^^,^^443^ Finally, anti-acne skin products can also be obtained from marine resources. The beneficial regulative effect of marine-derived components on the activity of sebaceous glands and sebum-binding ability can be confirmed by measuring sebum excretion by sebumetry. Briefly, on physical contact with the sebum present at skin surface, it is collected into a porous tape, with transparency depending on the amount of collected lipids. The translucency of the tape is then measured photometrically (Sebumeter) or visually (Sebutape).^444^

Parallel to instrumental evaluation, an important attribute is the assessment of the product tolerability by monitoring any potential adverse effects on the site of application by dermatologists to test potential adverse effects or reactions (e.g., irritation, sensitization).^98^^,^^445^ However, in contrast to numerous *in vitro* studies on skin bioactive properties of marine-derived ingredients, only a small number of human clinal studies has been performed so far.^446^ In these studies, subjects were chosen based on specific skin features and age/gender to evaluate cosmeceutical formulations with incorporated various macroalgae extracts. Overall, they reported skin moisturizing, anti-melanogenic and anticellulite (slimming) effects based on instrumental evaluation of the usual set of skin parameters such as skin hydration and barrier function, biomechanical properties, skin erythema or brightness, measurements of hyperpigmentation, and skin thickness, chosen depending on the desired benefit of tested macroalgae extracts. Product effectiveness can also be evaluated based on an expert opinion, e.g., dermatologically observed improvement of skin dryness following marine-based oil treatment^447^ or in the appearance of cellulite for formulations with incorporated marine cosmetic ingredients.^442^ Alternatively, questionnaires are used to obtain self-assessments upon the subjects’ satisfaction and subjective effectiveness of the tested product. The scientific discipline of sensory analysis, referred to as hedonic affective and effective testing, can thus provide valuable data including the consumer degree of acceptance, depending importantly (also) on the cosmetic products’ sensory benefits as perceived and evaluated by human senses.^448^ In the field of natural products like marine cosmetics, this discipline requires increased efforts because marine-derived ingredients could be linked with undesirable sensory characteristics.^449^ On the other hand, subjective efficacy is a crucial aspect for a product that is usually tested with subjects. These have often reported improved skin features for various skincare products with incorporated marine-derived compounds.^98^^,^^450^ In-depth self-evaluation sensory analyses of features related both to the product, such as appearance, fragrance, texture, spreadability and rapidity of absorption, as well as features connected to product efficacy, such as skin smoothness, firmness, softness and tone, along with reduction of circumference and reshaping and refining of the silhouette should thus be performed for products that incorporate marine ingredients.^443^

## Production

**Figure 10:**
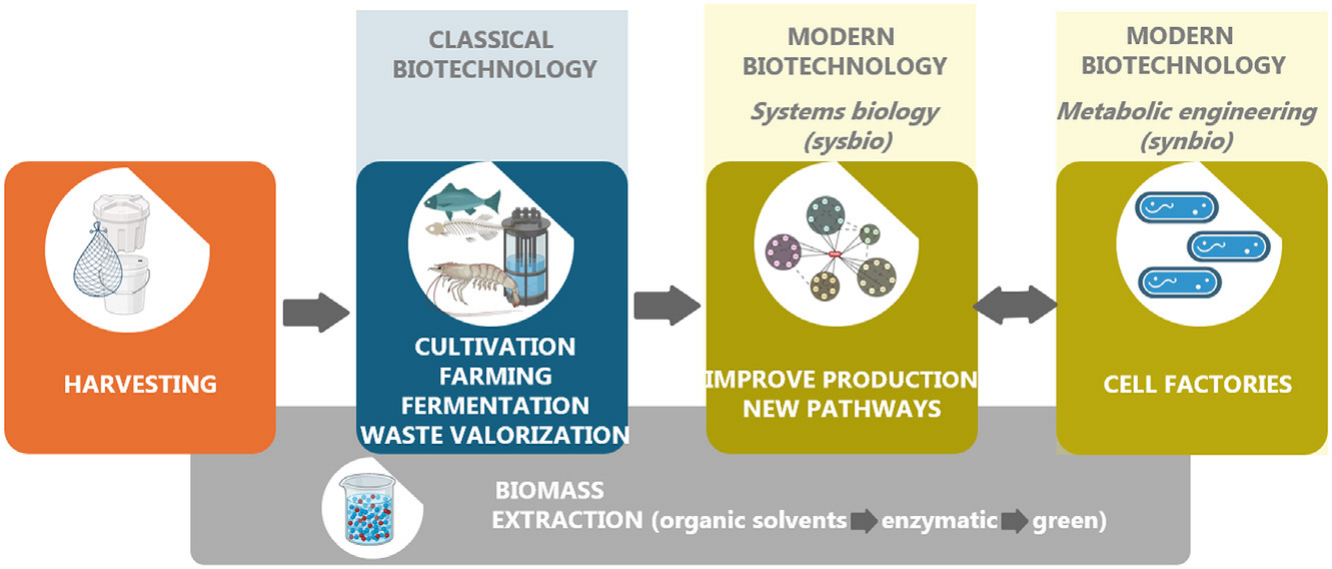
Approaches for sourcing biomass, bioproduction development and extraction of bioactive compounds Gray arrows show the chronological development of techniques: larger arrows represent the development of bioproduction; smaller arrows (in gray square) show the development of extraction methods. See text for more details. Some parts of the figure were created with BioRender.com.

The traditional production of nature-based compounds relies on harvesting organisms and biomass from their natural environments. However, using marine organisms for cosmetics might not be amenable in cases when exploiting natural resources would endanger the natural populations or ecosystems, or when the amounts of isolated compounds are very low and depend on environmental factors, making yields unstable. As sustainable sourcing and supply are bottlenecks in all nature-based applications,^121^ classical biotechnology approaches (Figure 10) have for years developed approaches for cultivation, fermentation and farming. Aquaculture of organisms is used to improve availability, ingredient supply, quality control, efficacy, traceability and security.^369^^,^^451^^,^^452^^,^^453^ This also includes methods for isolating/culturing invertebrate microbial symbionts^125^ which are often the main source of target bioactive compounds. More recently, valorization of waste/side-streams, such as beach wrack and fisheries by-products and discards has been considered as well.^454^

The controlled cultivation of microorganisms for their utilization in various industrial sectors is carried out in bioreactors. The bioreactor design depends on the cultivation mode (batch, fed batch, continuous), requirements of the organism (oxygen requirements, temperature, pressure, carbon source, pH, etc.), light irradiation (in the case of photobioreactors), contamination risk and salinity.^455^^,^^456^ In the last 20 years or so, along with the application of advanced immobilization and purification technologies, an increasing number of large-scale cultivations of marine bacteria has been reported.^457^

To improve yield and optimize the discovery processes of bioactive compounds, modern biotechnology has started using systems and synthetic biology approaches (Figure 10). Indeed, understanding the biosynthesis of active ingredients and its dependence on environmental factors is crucial for establishing stable production processes. Systems biology approaches, combining different – omics (transcriptomics, metabolomics, proteomics), bioinformatics and modeling can provide efficient growth practices and markers that can guide the production. Successful advances have been made in the last 20 years to reconstruct and analyze biological network models, however, marine systems biology is still in its infancy.^458^^,^^459^^,^^460^

The bioproduction of safe and effective active ingredients is not always possible. When microorganisms are difficult or expensive to grow or they are toxic to humans, cell factory engineering is required. Cell factories, with enhanced production of native compounds or heterologous production, can be assembled using metabolic engineering and synthetic biology approaches (Figure 10). Metabolic engineering was developed in the early 1990s^461^^,^^462^ and aims at pathway design, construction and optimization to produce different products, like fuels and pharmaceuticals using tools provided by synthetic biology.^463^

Marine microorganisms can be used as heterologous hosts. Photosynthetic cyanobacterium *A*. *platensis* (commonly known as Spirulina) has been established as an efficient bioproduction platform for different proteins.^464^ A multitude of developed synthetic biology tools for microbial metabolic engineering^465^ can increase the number of applications. GRAS microorganisms are the preferred bioproduction chassis. As an example, *Corynebacterium glutamicum*, a well-known producer of amino acid-based substances,^466^ has been used for the bioproduction of HA to avoid the potential pathogenicity caused by *Streptococcus* sp., the natural producer of this polysaccharide.^467^ Similarly, bioproduction of pseudopterosin-type precursor was established in *E*. *coli*.^378^ After the heterologous production is established, it might not be commercially applicable due to metabolic bottlenecks or signaling perturbations.^468^ Systems biology approaches have been shown to efficiently identify and correct the systemic causes of non-optimal production in plant and yeast bioproduction systems.^468^^,^^469^

The prerequisite for establishing heterologous bioproduction of active ingredients is to know all the enzymatic steps involved in their biosynthesis.^378^ These might not be known for some complex chemical structures. As marine organisms are largely undiscovered, this is a critical bottleneck in marine biotechnology processes. Nevertheless, marine organisms might provide a rich source of active ingredients that can be included in the newly generated heterologous pathways.^470^^,^^471^ Bioprospecting methods, based on sequence motif recognition, have contributed to the establishment of *de novo* indole production in *C*. *glutamicum*, offering an alternative to conventional production.^472^ Similar approaches are also expected for marine organisms in the future. In addition, marine organisms often exhibit low concentration of bioactive compounds and poor batch consistency (due to, for example, seasonal and geographical variability of production^473^), making large-scale production expensive. Harvesting can be sidestepped by establishing cultivation systems, though these come with technical challenges of their own. Finally, some bioactive compounds are unstable limiting their usability.^71^

After obtaining the desired quantities of biomass (either through valorization of side streams, culturing or cultivation), downstream processing is next. This step includes organism/cell harvesting and dewatering, followed by extraction methods.^426^

As the harvesting step can contribute up to 30% of the total production cost, efficient methods for maximizing biomass recovery, while minimizing energy and operational costs are being developed.^474^ Physical, chemical, biological, and electrical-based harvesting can be used independently or combined for maximum biomass recovery from the cultivation medium.^474^ Flocculation, centrifugation, filtration, sedimentation, magnetic separation and immobilization are all used for harvesting biomass.^475^^,^^476^^,^^477^^,^^478^ Each of these has their own advantages and disadvantages (in terms of energy demand, recovery, fouling and cost).^479^

The development of improved extraction methods to enhance the yield of bioactive compounds is a crucial step for the sustainable economic return and cost effectiveness of material supply, and the sustainable management of ecosystems. For example, although chlorophylls have shown promising antioxidant potential, their current extraction should be cautiously optimized to prevent the use of organic solvents.^426^ Green approaches using non-toxic, eco-friendly, and bio-based solvents should be developed and optimized to address the safety and low environmental impact of these ingredients.^480^ Alternatively, enzyme-assisted extractions for enriched extract release and recovery have also been developed.^481^^,^^482^ Recently, the focus has shifted to the use of green chemistry. This includes the development of novel extraction methods, such as ultrasonic-assisted, microwave-assisted, ionic liquids, subcritical water extraction, the use of deep eutectic solvents and pressurized solvent extraction.^483^^,^^484^^,^^485^^,^^486^^,^^487^^,^^488^^,^^489^^,^^490^^,^^491^ Their choice should be carefully planned as they differ in the extraction efficiency, price, energy consumption and the operating temperature which might lead to degradation of thermolabile compounds.

## Packaging

Cosmetics depend significantly on their packaging to protect their stability and properties. Since cosmetics are high-value goods, packaging has attracted a great deal of innovation and sophistication, offering a barrier against water, gases, UV light, and even external microbial contaminants.^492^

Petrochemical-derived polymers, also known as plastics, are the material of choice for packaging due to their many advantages: low weight, durability, malleability, strength, and transparency. Thus, both the rigid and flexible packaging used for bottles, pots, caps, tubes, and pills are mainly produced from high- and low-density polyethylene, polypropylene, and polyethylene terephthalate. Their practicality comes with several shortcomings, among them poor reusability, poor recyclability, and poor biodegradability. The accumulation of plastic waste and the risk of toxic chemicals leaking into the environment are a matter of pressing concern. Hence, rising public awareness has spurred policymakers and manufacturers to promote research into bio-based materials that can be recycled and composted.^50^ It is in this area where marine-sourced materials and compounds are becoming increasingly relevant.^493^ They can be produced sustainably and contribute to the recycling of otherwise wasted products. The valorization of new marine value chains can also incentivize local economies creating new job opportunities. Some of the biopolymers under consideration are polysaccharides from seaweeds, such as cellulose (it is highly hydrophilic, therefore not a good candidate for packaging, but has nonetheless been used in multilayers^492^), alginates (used as a biodegradable membrane with antimicrobial properties^494^), agar (can be mixed well with plasticizers to form elastic and soft gels^494^), and carrageenans (in mixtures with plasticizers such as polyols and blended with antimicrobial agents to achieve the controlled release of active compounds^495^), as well as chitin and chitosan from crustacean shells which are applied as a coating to bioplastics to protect perishable cosmetic products.^496^ In addition, the bioplastic precursors polylactic acid (PLA) and polyhydroxyalkanoates (PHA) can also be extracted from marine microorganisms.^497^ Regarding proteins, collagen and its derivative gelatine can be extracted from fishing waste/side streams (e.g., fish and jellyfish) and used in films and coatings for packaging. When blended with other substances, for instance chitosan and essential oils, they potentiate the packaging antimicrobial and photoprotection properties.^493^^,^^498^ Other marine products with applications in cosmetics packaging include minerals and salts (from mollusks), oils, muscle proteins, and pigments.^499^^,^^500^

## Regulatory framework

Researchers and industries developing new cosmetic products must comply with responsible research and innovation basis, the Nagoya Protocol and other marine biotechnology regulations. Collection of marine organisms is restricted by national and international treaties, such as the United Nations Convention on the Law of the Sea,^501^ the EU biodiversity Strategy 2030,^502^ the Nature Restoration Law,^503^ and the Marine Strategy Framework directive.^504^ In addition, efforts to patent genetic materials and compounds often face legal gray areas or unclear regulations.^505^^,^^506^^,^^507^ Moreover, licensing and patenting steps may significantly delay the time to market due to their lengthy processing time and the time and territorial coverage limitations.^508^ The full realization of bioactive marine natural products business potential in pharmaceutical applications is impaired by several obstacles. These include very rigorous clinical trials, extremely demanding certification, high purity, absolute structure characterization of the bioactive ingredient and providing a large scale raw material supply.^509^ Nevertheless, the development and innovation of other biotechnological applications called “low hanging fruits”, such as nutraceuticals/cosmeceuticals, which do not require such restrictive certification, purification and production conditions (where, e.g., crude extracts are commonly used), and that have a raw material supply with viable return, has attracted increasing attention due to their beneficial effects on skin care. These products are not regulated by drug regulatory agencies, such as the US Food and Drug Administration, and hence are not considered a separate category of cosmetics, forcing the consumer to rely on the self-regulatory policies of this industry, while still undergoing safety tests.^510^

Before entering the European market, cosmetic products must meet the requirements of the Regulation (EC) No 1223/2009 of the European Parliament and of the Council of 30 November 2009 on cosmetic products^511^ and its amendments. This is the main regulatory framework that ensures consumer safety. Cosmetic Product Safety Reports constitute a crucial component of the Product Information File, complementing the Description of manufacture method complying with GMP, as well as the Proof of claims and effects, among other elements. In parallel, stability and efficacy testing are a vital part of cosmetic product evaluation, alongside comprehensive toxicological assessments. More on the European legal requirements is already discussed within the sections “stability testing”, “safety”, and “efficacy and tolerability”, as well as synthesized in Document S1.

A specialized database with information on cosmetic substances and ingredients, called the Cosmetic ingredient database (CosIng),^273^ enables easy access to data on these substances, including legal requirements and restrictions. The International Nomenclature of Cosmetic Ingredients contains internationally recognized systematic names to identify cosmetic ingredients. These are developed by the International Nomenclature Committee (INC) and published in the International Cosmetic Ingredient Dictionary and Handbook.

## Limitations and bottlenecks

**Figure 11:**
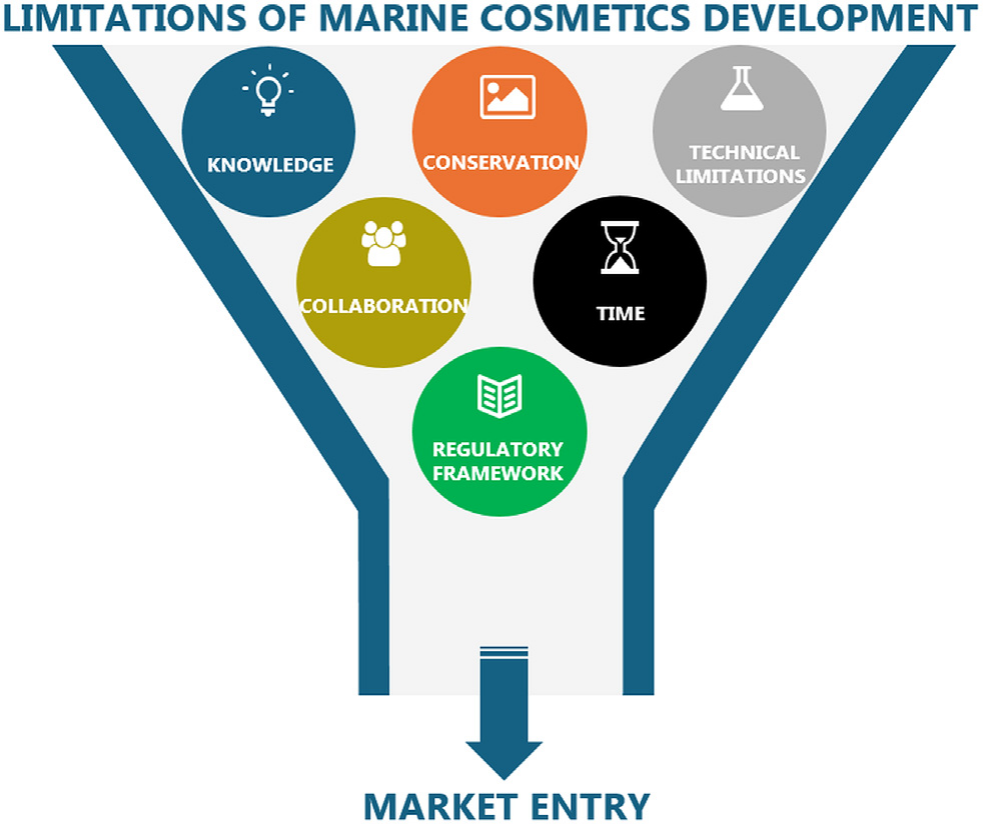
Six categories of bottlenecks before the market entry of new cosmetics products from marine environment

Despite the large potential of marine products for cosmetic applications, they also face numerous challenges. They can be grouped into six categories: state of knowledge, conservation, technical limitations, collaboration, time and regulatory framework (Figure 11).

First, marine organisms are still largely underexplored and undervalorized, especially in the extreme environments that require specific equipment, skills, and resources for long cruises, which include systematic mapping, especially in the deep ocean and seabed.^121^ This also includes the compounds synthesized by marine organisms, therefore the knowledge on metabolites, their bioactivity and metabolic pathways will remain an area of active research in future. Nevertheless, it is an area worth exploring as the likelihood of discovering previously undescribed species with commercial value is estimated to be 500 times higher in comparison with terrestrial counterparts.^512^ Second, harvesting aquatic organisms requires extreme care. The process may physically disturb other species or the trophic networks in which they participate. Responsible harvesting must also be taken into account, as excessive extraction can quickly deplete natural stocks (e.g., fish, sponges, sea cucumber, etc.). This has to take into account the effect of climate change which is changing the availability and distribution of marine organisms. For this reason, cultivation, culturing, systems and synthetic biology are preferably used instead of harvesting. The third challenge corresponds to technical limitations, which were addressed in the “Production” chapter above. The fourth limitation considers that from marine bioprospecting to placement of new products on market, a series of dynamic collaborations needs to be implemented. Indeed, science and innovation can only be conducted in groups, built upon collaborations, extending from basic research to applications and involving scientific, marketing, legal and industrial partnerships.^121^^,^^513^ This is not an easy process, and especially, in this context, the fifth challenge corresponds to the time and financial resources that need to be invested, which slow down the entire value chain development process of new cosmetics formulations Finally, the regulatory framework when developing new cosmetics from marine sources was already introduced in the previous chapters (“Legislation”, “Stability testing”, “Safety”, “Efficacy and tolerability” and Document S1).

## Sustainable bioeconomy

The concept of bioeconomy has been receiving increased governance attention in recent years. However, the current bioeconomy definitions address only its resource base, i.e., the renewable bio-based resources.^514^ But when addressing the opportunities for the cosmetics sector offered by marine organisms and their biomass, the environmental sustainability needs to be addressed as well. Indeed, harvesting resources might lead to overexploitation and ecological imbalances.^515^ The Icelandic example of a national strategy for sustainable harvesting is provided in Document S2.

Hence, to fully embrace the concept of sustainable bioeconomy in marine-based cosmetics, it should include sustainable sourcing, production and consumption, as well as circularity of processes.^514^^,^^516^ Indeed, circular economy is receiving increasing attention globally and is intended to integrate economic activity and environmental wellbeing.^517^ Microalgae, for example, can be cultivated in waste substrates, where they convert the residual nutrients into valuable substances. When the necessary health and safety concerns are addressed, extracts from such biomass can be used for cosmetics.^518^ An approach worth considering is the biorefinery concept. It can be described as the cascading integration of several biomass conversion processes to produce value-added products for the same or different sectors that are economically feasible.^519^ In a sustainable biorefinery approach, the process produces minimal waste and decreases the pressure on the ecosystem.^520^ This concept can be directly applied to cosmetics produced from marine biomass as the related products decrease the pressure on ecosystems with decreasing biological leftovers.

### The potential of algal biorefineries

The use of microalgae for cosmeceutical purposes is an attractive field that aims to meet the demand for natural ingredients in healthcare and cosmetic products. The increased interest in microalgae is associated with their rich content of high-value biological compounds such as peptides, carotenoids, lipids, carbohydrates, and MAAs. These components have been identified as useful additives for cosmetic products because of their strong bioactive properties.^521^ Due to their multiple potential benefits for various industries, the cosmetic industry is not the only sector that exploits microalgae to develop functional components. Indeed, microalgae have also received much attention in the pharmaceutical, biofuel, aquaculture and food industries. Given the diversity of possible application areas of microalgae, the development of an integrative and multifunctional process through the biorefinery approach can provide an opportunity to produce several biological products that serve different industries.^522^^,^^523^

The biorefinery approach is a promising route toward sustainability and economic viability, which aims at the conversion of available biomass into a variety of marketable value-added products. It is an industrial strategy incorporating different systems, procedures and engineering technologies by considering all steps of the process.^524^ As already mentioned, microalgae can be used to produce valuable compounds during the biorefinery process, depending on the selected strain, biochemical compositions, and target sectors. The main goals of a microalgae-based biorefinery are to optimize the utilization of algal biomass into multiproducts, maximize process yield, minimize waste generation, and promote the economics of microalgal biotechnology.^522^ In this context, recent studies have proposed different biorefinery strategies involving a large number of upstream and downstream steps. Upstream processing, which is mainly related to the cultivation stage of microalgae, involves several essential factors such as the type of strain, nutrient source, illumination, and CO_2_ supply.^525^ One of the interesting biorefinery approaches for upstream processing is based on the circularity concept, where cultivation of microalgae is done through an integrated system using wastewater, which contains different types of pollutants and nutrients (nitrogen, phosphorus, copper, etc.). The use of wastewater generated from various industries including dairy, agriculture, aquaculture, food or textile offers several advantages, such as (i) providing treatment of wastewater, (ii) reducing costs of microalgae cultivation, and (iii) contributing to zero waste concept.^526^^,^^527^ However, combining wastewater treatment and microalgae growth processes may become challenging when the obtained microalgal products are planned for use in the cosmetic industry. This is because the produced biomass and/or bioactive compounds may contain trace amounts of toxic chemicals found in wastewater. To eliminate the possible harmful effects, food or domestic wastewaters can be more appropriate nutrient sources for microalgae cultivation due to their non-toxic characteristics.^528^

Another critical stage is downstream processing which includes harvesting, cell disruption, extraction, and purification of target compounds from microalgal biomass. This step contributes to approximately 50% of the total production cost of microalgae-based products, and thus most of the biorefinery efforts have been focused on the improvement and integration of downstream processes.^529^ Recently, special attention has been paid to the co-production of high-value (e.g., cosmetics) and low-value (e.g., biofuels) products through the integration of downstream processes.^522^ Microalgal biomass, which has high lipid and carbohydrate content, can be converted into different types of biofuels, such as biodiesel, biomethane, biohydrogen and bioethanol, through different technologies. The advantages of using microalgae are high area productivity, they do not compete with food feedstocks, their positive effects on the environment, and simple operation conditions make them an ideal source to meet future global energy demands.^530^^,^^531^ However, biofuels obtained from microalgae are not economically competitive because of the high energy consumption of processes and low marketing value of the end-products. To overcome this challenge, a cascade utilization process has been proposed to comprehensively convert the whole biomass into a variety of commercial products.^532^ From the point of view of the cosmeceutical industry, microalgae-based polysaccharides, carotenoids, vitamins and MAAs are some of the favorable compounds that could be extracted from raw biomass. It is noteworthy to mention that these metabolites, together with proteins and phenolics, also find therapeutic applications in the pharmaceutical industry due to their strong bioactive properties e.g., antioxidant, anti-inflammatory, anti-carcinogenic. In addition, carotenoids, proteins, and fatty acids have received considerable attention in food and feed applications as food additives and natural colorants owing to their nutritional values and rich pigment content.^527^ Given the variety of high-value compounds and their potential application areas, stepwise extraction and fractioning processes can contribute to microalgal biorefinery through the complete valorization of biomass or residual by-products. After the extraction of valuable compounds, the microalgae residue still contains high amounts of lipids or carbohydrates, which can be converted into different types of biofuels. Conversely, an opposite scenario is also applicable for biorefinery processes in which lipid is firstly extracted from raw biomass and then it is converted into several high-value products.^523^^,^^529^ In addition to high-value applications and biofuels, final residual biomass can be used as fertilizer in agricultural activities to improve the electrical conductivity and pH of soil, promote the growth of crops, inhibit the growth of fungi and plant pathogenic bacteria.^533^

Numerous studies have investigated the development of high-yield and energy-efficient biorefinery processes for microalgal products. For example, Djamai et al.^534^ developed a membrane-assisted process to fractionate all compounds from a mixture of phytoplankton with a sustainable biorefinery strategy. This system provided the extraction of triacylglycerols and pigments through solid-liquid-liquid extraction method, and then fractionated the proteins and carbohydrates by using a membrane filtration process. These compounds have the potential to be applied in cosmetic and pharmaceutical industries. In another study, *Nannochloropsis* sp. was used as a biomass feedstock to produce fatty acids, biohydrogen, and added-value compounds by performing a biorefinery strategy. In this context, supercritical CO_2_ extraction method was first applied to recover lipids and carotenoids. After that, the remaining biomass was used as a substrate in a dark fermentation process to produce hydrogen. Within the scope of this work, a promising strategy was presented for the utilization of residual biomass after extraction of high-value compounds, which can be used as cosmeceuticals.^535^ Apart from experimental studies, the concept of biorefinery has also been explored at the theoretical level through models and simulations. In the work of García Prieto et al.,^536^ a mixed integer nonlinear programming model was proposed for the production of biodiesel, astaxanthin, and polyhydroxybutyrate (PHB) through an integrated biorefinery concept. They concluded that the production of astaxanthin and PHB was a promising approach to make biodiesel production cost-effective. Considering the diversity of microalgae-based products and the complexity of downstream processings, a cascade utilization process presents several bottlenecks that are mainly related to separating the different fractions and minimizing the loss of product yield at each stage. Also, this process may not be cost-effective, since many microalgal compounds have not yet been explored. To overcome these drawbacks, a fully integrated biorefinery process should be performed by taking into account the physicochemical properties of fractionated compounds, extraction methods, energy consumption of processes, environmental impact, and economical sustainability of the developed concept.^522^^,^^537^

**Figure 12:**
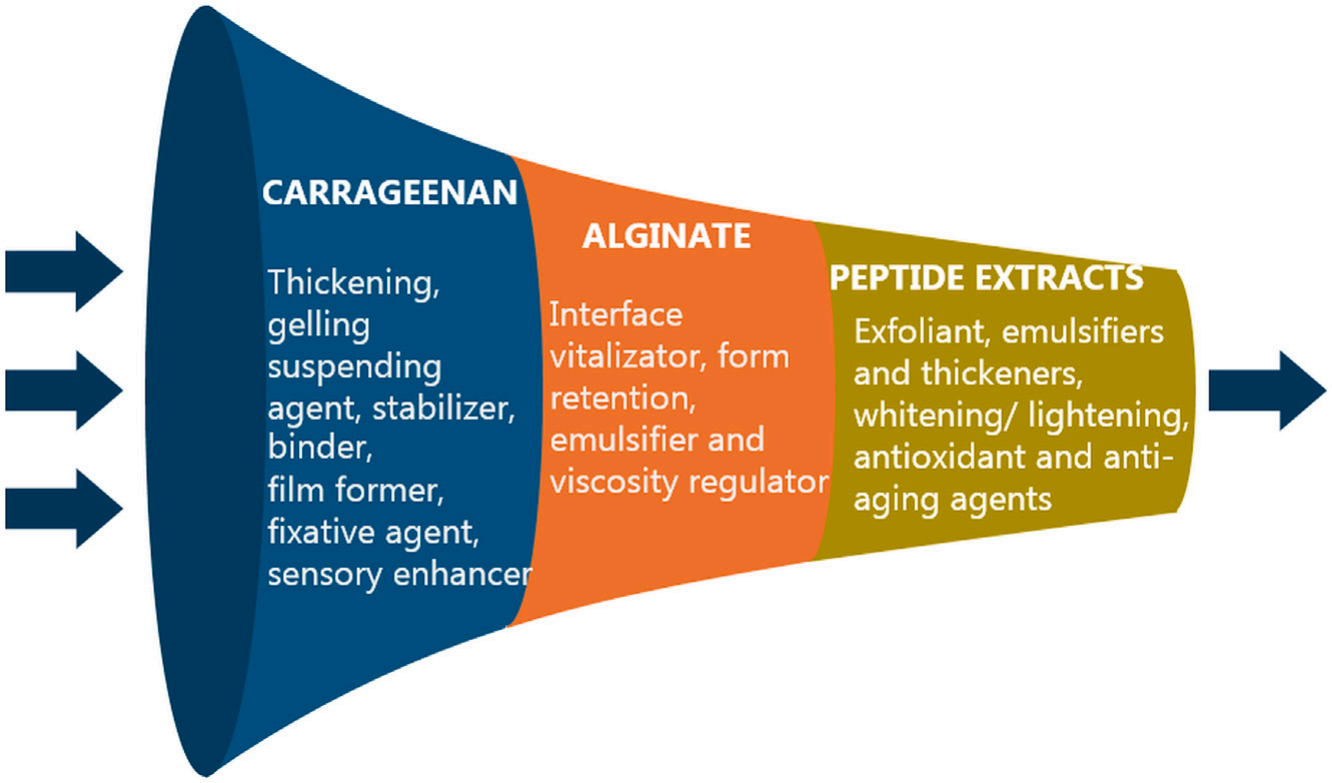
The biorefinery concept example for the use of seaweed in cosmetics Each extraction step (carrageenan, alginate, peptide extracts) is presented in a different color and the main uses for each step are listed. Find in text the presentation of cosmetic products for such a biorefinery approach.

An additional example for possible applications in cosmetics can be offered by the seaweed biorefinery concept, which has been getting increased attention. Figure 12 summarizes the biorefinery concept of macroalgae and applications.^519^^,^^520^^,^^538^ Carrageenan can be used in skin care and lotions, hair care products, eye make-up, toothpaste, shaving foams and stick applications.^539^ Alginate is suitable for face (anti-acne agent) and body care, hair care and body cleansing, color cosmetics and sun care.^539^ Peptide extracts can be used in face creams, body lotions, shampoos, hair sprays, sunscreens, and bath products.^540^

## Case studies

### Macroalgal bioactive ingredients and skincare product design in Iceland

Seaweeds are rich in bioactive compounds that can provide many different applications in the food, pharmaceutical, and cosmetic industries. Phlorotannins, the major polyphenolic compounds in brown algae, have high *in vitro* antioxidant activity.^541^^,^^542^ To assess this, ten species of seaweeds commonly found along the costal line of Iceland were screened using three *in vitro* antioxidant activity assays.^541^ Furthermore, the correlation between total polyphenol content (TPC) and antioxidant activities was investigated to characterize the antioxidant properties. The results indicated that among those investigated, the brown seaweed *Fucus vesiculosus* (Linnaeus) had the highest TPC, strongest scavenging activity against DPPH and peroxyl radicals, and a moderate ferrous ion-chelating ability.

**Figure 13:**
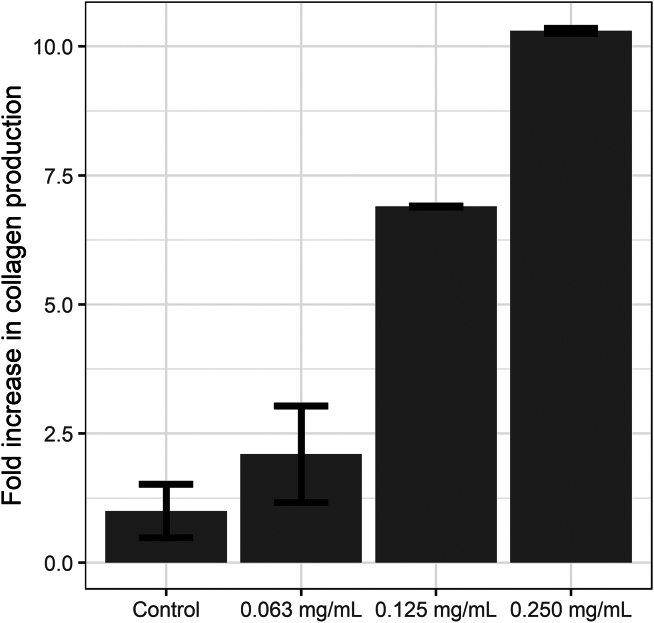
Cellular collagen production in D551 skin cell lines exposed to varying amounts of *F. vesiculosus* extract (0.063 mg/mL – 0.250 mg/mL) compared to a blank (control). The error bars represent the measurement +/- standard deviation

Based on previous results,^541^ further studies were carried out on *F. vesiculosus* aqueous extract as a potential cosmetic ingredient. These suggested that phlorotannins were the active components in *F. vesiculosus* extract.^543^^,^^544^ In addition, they highlighted the ability of *F. vesiculosus* extract to stimulate collagen production (Figure 13), which has significant potential for cosmetics industry. The *F. vesiculosus* extract was also able to effectively reduce the activity of MMPs, thereby having a positive effect on skin health.^545^

#### Stability tests of Fucus vesiculosus extract

**Figure 14:**
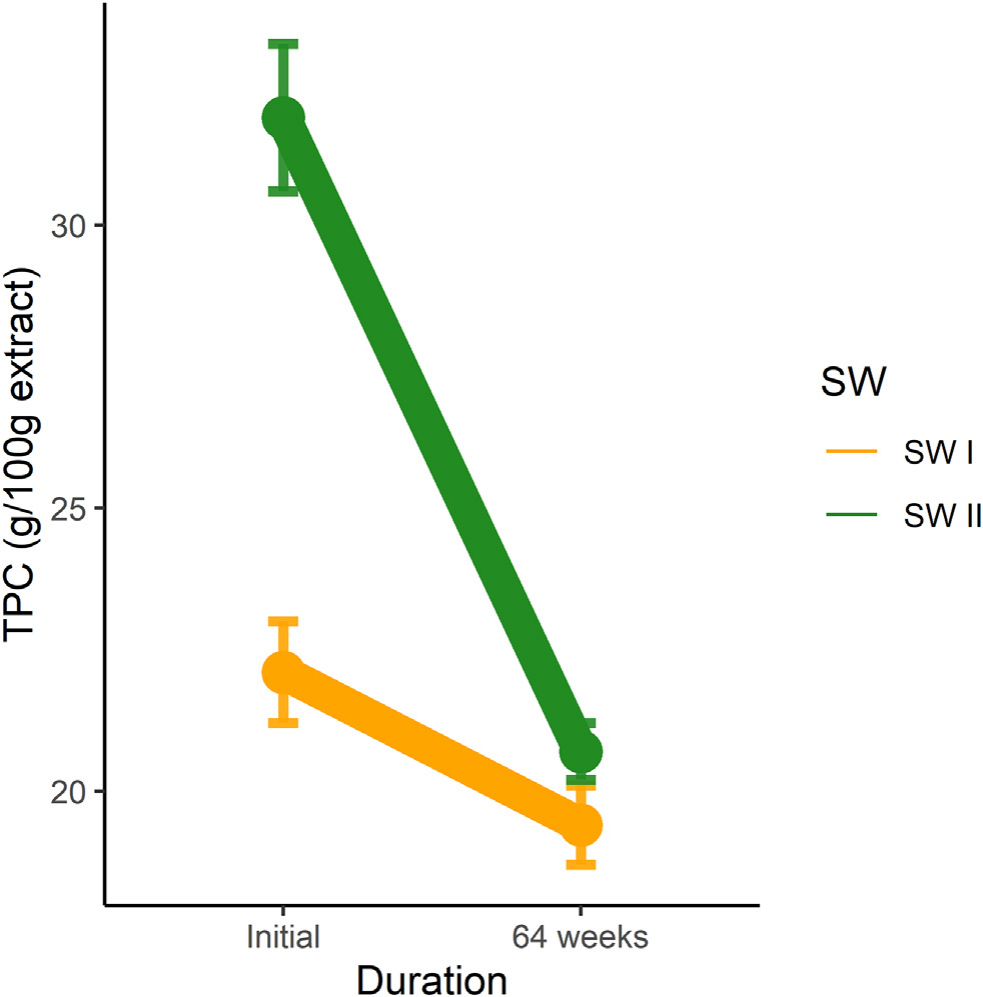
Total polyphenol content (TPC, g/100g extract) of seaweed extracts (SW I and SW II) from two different seasons, June (SW I) and September (SW II) stored at room temperature for 64 weeks. The error bars represent the measurement +/- standard deviation.

To ensure that seaweed extracts meet the necessary quality standards for their use as cosmetic ingredients, stability tests were performed. These had two approaches: (i) storage for a longer period or (ii) storage under high temperature conditions, e.g., at 40°C for three months. The stability of *F. vesiculosus* aqueous extract was studied to test the storage stability of water extracts.^546^ The extracts were kept at two different storage temperatures (4°C and −18°C) for 64 weeks, with and without the addition of ascorbic acid. The brown algae *Fucus vesiculosus* (Linnaeus) was collected in Reykjanes peninsula, in two different seasons, i.e., June 2011 (SW I) and September 2011 (SW II). The TPC of SW II kept at room temperature was significantly higher compared to SW I, or 32.7% of TPC versus 21.9% TPC, respectively (Figure 14), indicating seasonal variations. No significant changes were seen in the TPC of SW I during storage time. However, the TPC significantly decreased in SW II after 64 weeks of storage. A similar trend was seen in oxygen radical absorbance capacity (ORAC) values and reducing power, but no significant changes were observed in DPPH radical scavenging properties (results not shown). Neither temperature nor storage time affected the antioxidant activity, except for SW II when kept at room temperature, which had lower ORAC and reducing power after 64 weeks compared to samples kept at 4°C or −18°C. The antioxidant power was similar at the beginning of the study and after 64 weeks. Adding ascorbic acid to samples kept at −18°C did not result in a significant improvement of storage stability.

#### Designing skincare products

**Figure 15:**
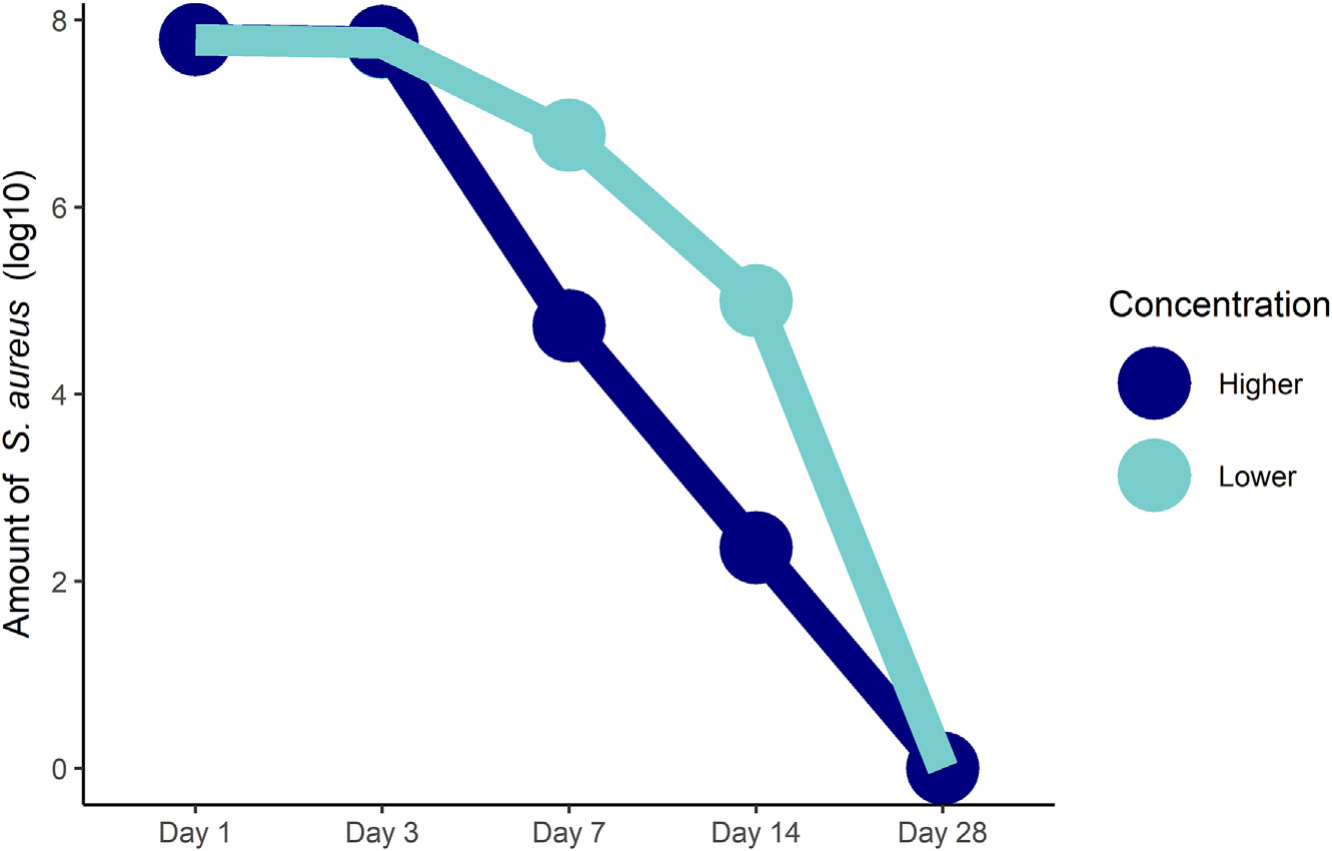
Dose-dependent antimicrobial effect of seaweed extract (with their higher or lower concentration in the final formulation) on the survival on *S. aureus*

Commercial cosmetic products were developed using *F. vesiculosus* extracts as a bioactive ingredient, resulting in the launching of the Icelandic company UNA skincare in 2012, branding a facial day cream and eye cream. The product development included a market study, focus groups, cosmetic product development as well as cosmetic product design or formulation. During the product development process, different types of bases were tested as well as different concentrations of seaweed extracts in addition to other ingredients. Furthermore, several stability tests, assessing both functional and sensory properties, testing of the efficacy of antimicrobial preservation (Figure 15), and consumer tests were carried out. These studies showed that both water and acetone *F. vesiculosus* extracts (at 2 mg/g of emulsion) have a protective effect against thermooxidation skin care emulsions, but only the water extract displayed antioxidant activity against photooxidation.^547^

#### Human intervention study

**Figure 16:**
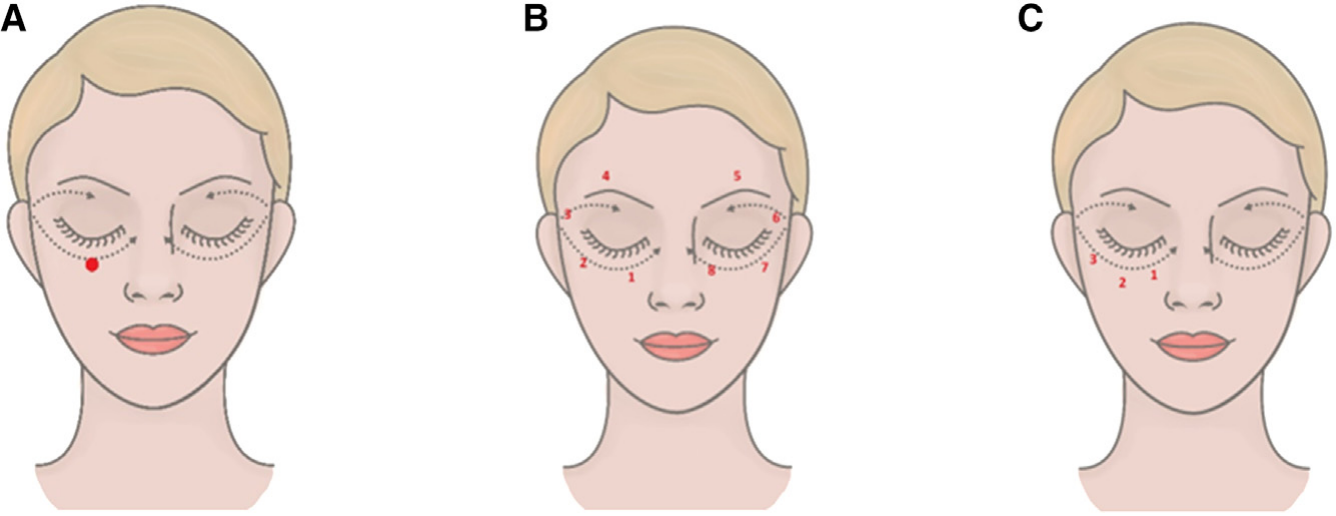
Human intervention study measurements Measurement points for (A) elasticity; (B) hydration; (C) collagen.

The effects of the skin cream containing bioactive *F. vesiculosus* extract were studied using a double-blind human intervention study.^548^ This was done by involving two comparable groups of people to test either a skin cream containing a bioactive seaweed extract, or a placebo cream containing all the same ingredients aside from the extract. The impact of the creams was measured three times over a period of twelve weeks. The skin of the participants was measured with a Dermalab Series Clinique Combo from Cortex technology, which gathered data about skin elasticity, collagen intensity, skin thickness and hydration (Figure 16). For elasticity, two parameters were measured: E (Young’s modulus) and RT (retraction time). The first measurement was performed at the beginning of the study period, before the participants were given their skin creams, the second after six weeks, and the third after twelve weeks which was the endpoint of the intervention period. During these twelve weeks, participants were asked to use the provided skin cream daily, in the morning and before bedtime. The inclusion criteria for participation in the skin cream intervention study were the age between 40 and 60 years and having generally healthy skin. Exclusion criteria were underlying skin disease(s) and history of using products from UNA skincare. Seventy-three (73) participants were recruited. Four did not continue after the first skin measurement, and in the end, sixty-six (66) participants completed the study, thirty-three (33) in each group. The average age was 48 years and 47 years in the groups receiving extract cream and placebo, respectively. When the study was finished, the participants were asked to answer a short online survey, using the program SurveyMonkey, where they were asked to give their opinion of the cream, e.g., its odor and texture.

Elasticity generally decreases with age^549^ and E has been found to increase linearly with age. A difference was seen for E in the first measurement (baseline) where participants using *F. vesiculosus* extract cream had a slightly higher average value than participants using the placebo cream (Table 3). Changes over time were detected in the mean values for both parameters E and RT. E decreased with time and was lower in the third measurement than in the first (*p* = 0.003). Elasticity increased over time for both groups of participants. The increase was more noticeable in the group using the cream with the bioactive extract compared to the control cream (*p* = 0.014) in the third measurement where a significant difference in E was seen between the two groups. Retraction time also decreased substantially with each measurement (p ≪ 0.0001) but no difference was seen between groups for this parameter.

**Table 3:** Average values of Young’s modulus (E) and retraction time (RT), for the baseline (first measurement) and differences in measurement 2 and 3 compared to baseline (Δ1 and Δ2 respectively)

		Extract cream	Placebo cream	*p*-value
E (MPa)	baseline	6.0 ± 2.88	5.2 ± 2.67	0.002
	Δ1	−0.5 ± 1.80	−0.3 ± 1.54	0.322
	Δ2	−0.8 ± 1.96	−0.4 ± 1.34	0.014
RT (ms)	baseline	764 ± 292.3	720 ± 362.8	0.177
	Δ1	−230 ± 311.5	−188 ± 286.2	0.154
	Δ2	−267 ± 287.7	−227 ± 398.7	0.262

MPa – megapascal, ms – milliseconds. p-values were calculated to compare the two groups of participants with the two-sample t-test.

Ultrasonic skin imaging was used to measure collagen intensity and skin thickness. The intensity correlates with the density of collagen in the skin and the thickness of the skin generally decreases with age.^549^ During the study, intensity did not increase in the skin of the participants. However, an increase in skin thickness was observed when using the cream with bioactive extract (*p* = 0.006, Table 4). Another interesting parameter is skin hydration, which increased with the regular use of creams. At the end of the intervention study, a trend for higher hydration (*p* = 0.071, Table 5) was observed in the group using the cream with the bioactive extract compared to the placebo group.

**Table 4:** Average values of intensity and skin thickness for the baseline (first measurement) and differences in measurement 2 and 3 compared to baseline (Δ1 and Δ2 respectively)

		Extract cream	Placebo cream	*p*-value
intensity	baseline	26.7 ± 8.22	27.7 ± 9.56	0.100
	Δ1	1.9 ± 8.40	0.5 ± 9.69	0.032
	Δ2	−1.7 ± 7.73	−1.8 ± 8.69	0.856
thickness (μm)	baseline	1275.7 ± 306.4	1304.6 ± 288.3	0.163
	Δ1	−14.5 ± 312.4	−7.6 ± 295.2	0.744
	Δ2	34.9 ± 301.7	−26.37 ± 325.0	0.006

Intensity – intensity of the acoustic response from the skin, μm – micrometers. p-value: comparison between the two groups of participants with two-sample t-test.

**Table 5:** Average values of hydration, for the baseline (first measurement) and differences in measurement 2 and 3 compared to baseline (Δ1 and Δ2 respectively)

	extract cream	placebo cream	*p*-value
baseline (μS)	234 ± 39.9	235 ± 42.3	0.907
Δ1	31 ± 33.7	41 ± 44.3	0.277
Δ2	29 ± 38.0	11 ± 42.2	0.071

μS – microSiemens. p-values: comparison between the two groups of participants with two-sample t-test.

In conclusion, the skin cream containing the bioactive seaweed extract had a positive impact on the skin of the participants. However, the group using the placebo cream also experienced positive results and often the differences between the two groups were not significant. Other external factors may have an impact on the skin, such as the hydration level in the atmosphere which increased during this trial period, starting in wintertime and finishing in spring.

The results of the questionnaire indicate that both creams were generally well-liked by participants. The majority detected a positive change in the skin after using them regularly, mostly connected to better hydration of the skin. The creams were rather greasy, which was well accepted by some participants. The odor was most often described as weak or neutral with no perceived difference between the two cream types. As a result, the UNA skincare products with *F. vesiculosus* extract ingredient were successfully launched.

### Microalgal cosmetics and spin-off creation in Italy

Natural extracts from microalgae have properties useful for cosmetic applications, among which antioxidant and anti-inflammatory activities are among the most salient. Preparations showing these effects can prevent the oxidative stress induced by pollutants, smoke, UV radiation and unhealthy eating habits, thus reducing the risk of cellular damage and chronic inflammation, eventually resulting in an antiaging effect. *Tetraselmis suecica*, a marine green microalga belonging to the class Chlorophyceae, is extensively used in aquaculture as a feed for mollusks and crustacean larvae,^550^^,^^551^ and as a probiotic in fish aquaculture.^552^ *T. suecica* is rich in valuable compounds, such as vitamin E, carotenoids, chlorophyll, and tocopherol. Hence, it has been suggested as a food supplement in human and animal diets.^553^ Other possible applications of *Tetraselmis* sp. include the use of total pigment extract to enhance dermal pigmentation, reduce psoriasis lesions and increase hair growth.^554^

**Figure 17:**
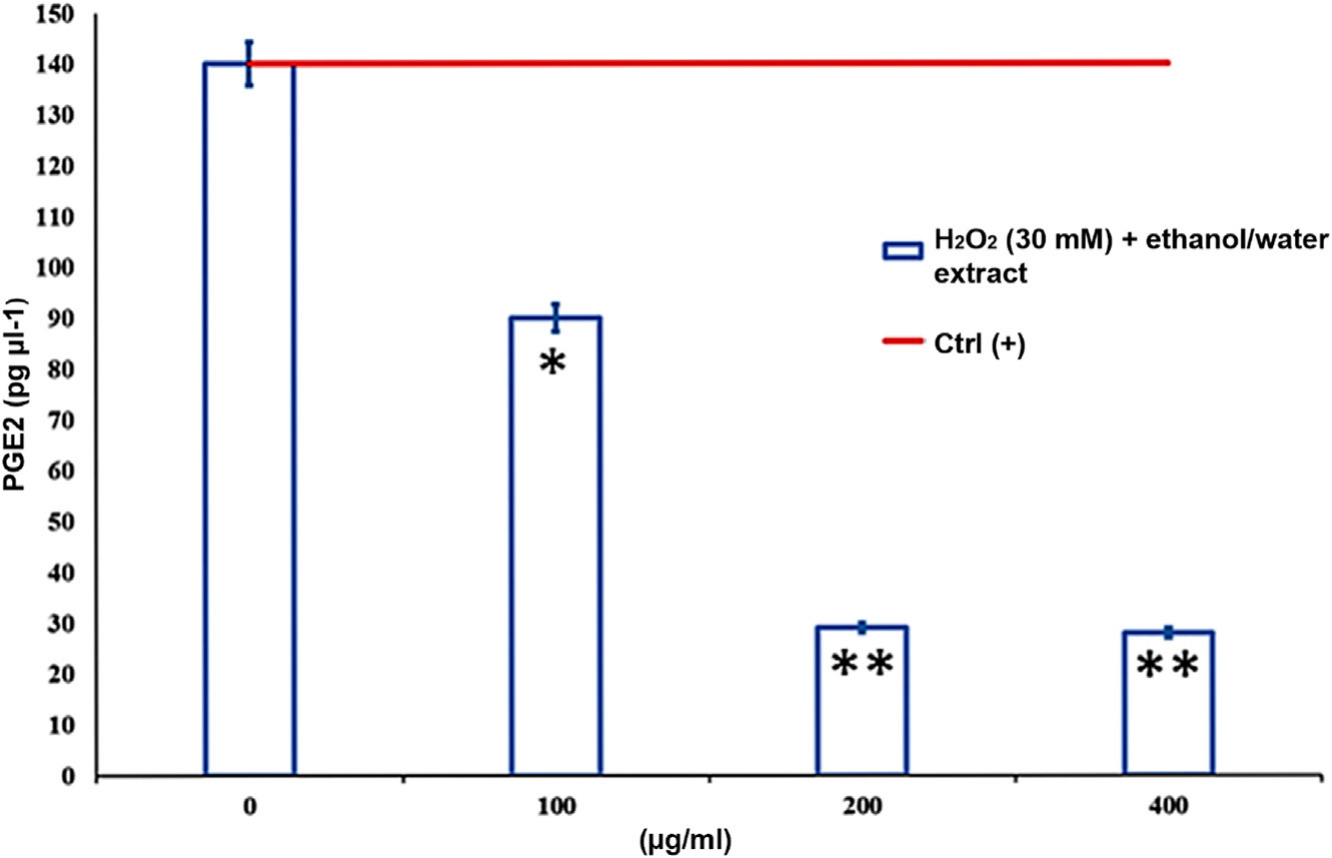
Effect of *Tetraselmis suecica* ethanol/water extract on prostaglandin PGE2 release induced by H_2_O_2_ treatment in human lung adenocarcinoma cells (A549). The error bars represent the measurement +/- standard deviation. The average PGE2 concentration (pg μL−1) was determined by ELISA in culture media of cells treated with 100, 200 and 400 μm g mL^−1^ of extract for 24 h after pretreatment with 30 mM (=12 μm g mL^−1^) of H_2_O_2_ for 1 h (modified from^555^).

It was shown that the ethanolic extract of *T. suecica* has a strong antioxidant and cell repairing activity in the A549 human lung cancer cell line.^555^ The extract also displayed the repairing properties when applied to epidermal cells and to reconstructed human epidermal tissue cells (EpiDerm), preventing damage induced by H_2_O_2_. The effect of the extract at the molecular level was assessed by the evaluation of expression patterns of genes, involved in the response to oxidative stress, comparing cells treated only with 30 mM H_2_O_2_ with cells recovered with 100, 200 and 400 μg mL^−1^ extract. The genes ATOX1, CCL5 (RANTES), DHCR24, FOXM1, GPX1, GPX4, PDLIM1, PRDX5, SIRT2, SOD2, involved in antioxidative, regulatory, anti-inflammatory processes, were all significantly upregulated in a dose-dependent manner (Document S3), which indicates the activation of a recovery process. This extract was demonstrated to target the expression of dehydrocholesterol reductase-24 (DHCR24) and prostaglandin reductase 2 (PTGR2), and to reduce the levels of prostaglandin E_2_ (PGE_2_) released after treatment with H_2_O_2_ (Figure 17).

Based on these pieces of evidence, the bioactive extract from *T. suecica* was developed as a cosmetic formulation by the Italian start-up company Biosearch s.r.l.,^556^ born as a spin-off of the Italian National Research Council (CNR) and the Stazione Zoologica Anton Dohrn (SZN) for the development of new drugs and cosmetics from marine biological sources. The company’s activity focuses on technological innovation aimed at the discovery of new bioactive compounds, to improve production processes and optimize product formulations.

Along the process, the major challenge was the identification of a company that could take care of safety and efficacy tests, and possibly entrusting the same enterprise for the commercialization of the final product(s). Indeed, as already mentioned, before extracts or compounds can be ready for the market, several steps are required. First, it is necessary to prepare a formulation that can preserve the bioactivity and favor the beneficial action of the active ingredients that should be present at concentrations that have shown significant activities in *in vitro* tests. The formulation should be stable and should include only few ingredients to satisfy the concept of essentiality. Finally, it must be tested for safety, evaluating skin/ocular irritancy potential *in vitro* and skin compatibility under dermatological control using a patch test. Microbiological tests are also crucial and consisted in the evaluation of the antimicrobial protection of the formulation (ISO 11930:2012), sterility test (ISO 11737-1:2006), microbiological assays employing *Escherichia coli* (ISO 21150:2016), *Listeria monocytogenes* and *Listeria* spp. (ISO 11290-2:2017), and counting and serotyping of *Salmonella* spp. (ISO 6579-1:2017). These tests can be quite expensive; thus, another challenge for a start-up is the availability of sufficient funds to afford these expenses.

For the development of new cosmetic products based on *T. suecica* extract, Biosearch established a collaboration with PriGen, a cosmetic company based in Milan, Italy^557^ to identify the most effective formulation without side effects. PriGen is the owner of a patented new glycerosomial technology for the controlled release of active ingredients in the cosmetic and biomedical field. This technology was used to create a line of skincare products named Gen-Hyal that included three new products using the *T. suecica* extract. These target different needs for cosmetic applications: a daily face serum with detoxifying and soothing active ingredients, presenting regenerative action and an anti-pollution effect; an antiaging UV shield, a cream with SPF 30+ UVB/UVA sunscreen and HA; and a Bioactive LipCare capable of preventing chapping and redness, while ensuring effective antiaging and antioxidant action.

### Marine proteins for cosmeceuticals

Marine fish and jellyfish have been identified as a rich pool of bioactive proteins and peptides, having antioxidant, antibacterial, antiaging and tissue regeneration activities. So far, marine fish/jellyfish-derived proteins have been proven to be biocompatible and effective in cosmeceutical industries. Herein we present selected successful case studies where marine-derived proteins were isolated and used in skin care products.^558^^,^^559^

Among the many studied marine-derived proteins, collagen is the most widely commercialized protein, owing to its multiple bioactive features. Those most valuable to cosmeceutical products are its high absorption capacity, low odor, biocompatibility, and strong structure. In addition, due to religious and health-related considerations pertaining to mammalian-derived collagen, the one sourced from marine organisms has been identified as more appropriate for use. The highest amounts of collagen have been found in fish and jellyfish. Fish bones and skin, which are rich in collagen, representing up to 75% of their mass and fish processing industries usually consider it as a by-product, making them a valuable source for collagen extraction and further application.^559^ Jellyfish have undergone a major temporal and spatial increase in the last few decades in some ecosystems and are present in nearly all marine ecosystems worldwide, where they are often considered a nuisance. When jellyfish blooms occur, they can negatively impact fisheries by clogging fishing nets.^560^ These blooms can provide a potential source of jellyfish for collagen extraction.

There are multiple studies on collagen extraction from different marine species, where various extraction methods have been used. For cosmetic applications they mostly involve acid- and pepsin-assisted collagen extraction methods, mainly from skin and bone. In jellyfish, all parts of the animal have been used for collagen extraction, while the bells being the most predominant source.^561^ Marine-derived collagen is primarily type I fibrillar collagen, which is also the most abundant type in the human body. Jellyfish-derived collagen is rather referred as type 0 due to its similarity with several collagen types (I, II and V).^140^

Jellyfish-derived collagen is successfully used by two companies, OceanBASIS and Jellagen (Table 6).

(1) OceanBASIS GmbH is a company based in Kiel (Germany) with a cosmetic product line named Oceanwell.^562^ They are a group of marine biologists, engineers and skin experts dedicated to exploring bioactive marine compounds. For their cosmetic line they use the kelp *Saccharina latissima* from the Baltic and North Sea, and jellyfish-derived collagen from “root mouth jellyfish” from Asia. They have been able to obtain one liter of collagen from an individual jellyfish, which is enough for manufacturing 400 bottles of 30 mL each. In their ProAge Line they offer face cream, protective serum, cell boost elixir as well as clean and care cream, all based on bioactive collagen. Marine collagen has optimal features regarding skin-hydrating and skin-firming effects; while the serum showed better moisturizing effects in a short time, the face cream had a similar effect after a longer application period.
(2) Jellagen^563^ is a biotechnology company based in Cardiff (United Kingdom) founded in 2015 and is mainly focused on tissue regeneration applications with jellyfish-derived collagen. They are pioneers in demonstrating the unique advantages of collagen type 0 over mammalian-derived collagens. They manufacture medical grade collagen type 0 from *Rhizostoma pulmo* collected in the UK and the EU. They offer various formulations (liquid, hydrogel, scaffolds, dressings and flowable matrix) for multiple applications (orthopedics, cardiovascular, bone, wounds, cell and gene theraphy, and cell culture).^564^ Their studies showed high collagen biocompatibility, anti-inflammatory effects and tissue repairing properties (bone growth and wound healing).

**Table 6:** List of cosmetic companies with marine-derived proteins with information on species, site of collection and application

Company	Protein	Fish/jellyfish species	Site of collection	Application
OceanBASIS^565^	Collagen	“root mouth jellyfish”	Asia	Skin care
Jellagen^563^	Collagen	*Rhizostoma pulmo*	UK and EU	Tissue regeneration
Aqua Bio Technology ASA^566^	Salmon roe enzyme	Salmon	a	Skin care

a No data.

The other protein that is used commercially in skin care industries is a salmon roe (eggs or sperm) enzyme isolated from hatching water, previously regarded of as waste. Around the year 2000 it was observed that hatchery workers in Norway had very smooth skin on their hands, which appeared to be younger than their true age. Scientists from Aqua Bio Technology ASA^567^ recognized this high potential and later patented salmon roe enzyme as Aquabeautine XL. This enzyme, which beneficially exfoliates the skin, showcases its potential for correcting uneven tone, reducing lines, enhancing elasticity, without causing harm even after prolonged use. The enzyme is used as an ingredient in skin care lines in Aqua Bio Technology ASA brand Seidr^568^ and Restorsea^569^ (based in the USA) that are tailored for sensitive, dry, and problematic skin types. Aquabeautine XL is exclusively targeting dead cells, making it milder compared to retinols and glycolic acid. Its larger molecules also ensure gentler penetration. This approach avoids dermal damage, redness, peeling, and sensitivity often linked to traditional exfoliants, which often require sun protection warnings in labels.^567^^,^^570^

The use of jellyfish/fish biomass for extraction of bioactive compounds has its advantages and disadvantages. The successful examples are presented above, but there are many obstacles and challenges that can hamper the commercialization of marine-derived bioactive compounds for cosmetics use, such as: non consistent supply of biomass, lack of investments for infrastructure and advanced technology for biomass processing, strict regulations and cost effectiveness. Jellyfish blooms are considered as nuisance but still their occurrence is seasonal and not regular. Moreover, their body mass consists mainly of water and salt (approximately 95%), which makes it more difficult to process the whole biomass. 10 years ago, a start-up Cine’al^571^ was launched, which aimed to produce environmentally friendly diapers and other absorbent products made from jellyfish. They created a biopolymer “hydromash” that was synthesized by adding nanoparticles to jellyfish biomass. Unfortunately, currently there are no available data on the success of the start-up. Overcoming multiple challenges involves indeed advancements in science and technology, as well as strategic planning to ensure sustainability, supply and market acceptance.

## Conclusion

As the global population is seeking a better quality and longer life, the significance of health and wellbeing emerges as a paramount societal concern. In response, the cosmetic industry is shifting toward nature to source ingredients that offer enhanced functionality and contribute to environmental sustainability. This transition toward greener and more eco-conscious formulations is evident in the cosmetic and cosmeceutical sectors that are now turning their attention to the riches of the ocean for innovative ingredients, offering significant opportunities for innovation.

This review outlines a comprehensive framework of activities and essential considerations for the development of new value chains in marine cosmetics. Unlike previous approaches that often focused solely on organisms or specific activities, our perspective offers a holistic view of the process. This manuscript can thus serve as a valuable knowledge resource for researchers, industrial producers and policy makers, offering a singular guide to understand the complexities in the production of ‘blue cosmetics’. It delineates the organisms and compounds worth targeting and elucidates the multifaceted approach required to navigate this field effectively. It is important to highlight that despite its great potential, the development of marine cosmetics demands several *trans*-disciplinary and *trans*-sectorial collaborations, rounds of financing, management of potential intellectual property rights, addressing sustainability, safety and other regulatory bottlenecks. Therefore, well-planned strategic and technical processes are needed, along with time, resources and personnel commitment before starting the launching of new marine cosmetics on market. When all of the demands will be addressed, this field will progress at the pace that fits the increasing demands, offering consumers effective, safe, tested, environmentally sustainable and affordable products.

## Acknowledgments

This publication is based upon work from COST Action CA18238 (Ocean4Biotech), supported by 10.13039/501100000921COST (European Cooperation in Science and Technology) program. This publication is based upon work from Ocean4Biotech, the professional association joining marine biotechnologists. A.R., E.G.B., D.B., L.Z., K.K., A.Z.P., M.G.M., Š.B., and A.C.R.: This publication was produced with financial assistance of the Interreg MED Programme, co-financed by the 10.13039/501100008530European Regional Development Fund (Project No. 8MED20_4.1_SP_001, internal ref. 8MED20_4.1_SP_001)—B-Blue project. This publication has been produced with financial assistance from the Interreg Euro-MED Programme, co-funded by the 10.13039/501100000780European Union (Project No. Euro-MED 0200514) – 2B-BLUE project. The authors acknowledge the financial support of the 10.13039/501100004329Slovenian Research Agency (research core funding No. P1-0189, P4-0432, P4-0165, and project L4-4564). A.R., E.G.B., D.B., L.L.B.: acknowledge the financial assistance of the Interreg Euro-MED Programme, co-financed by the European Union (Project no. Euro-MED0300730—C4Nature)—Community4Nature. D.V.-M. and M.M.: This work was implemented in the framework of the research project SPINAQUA (Grant No. 239) funded by the 10.13039/501100013209Hellenic Foundation for Research and Innovation and the 10.13039/501100003448General Secretariat for Research and Technology under the “1st call for H.F.R.I. Research Projects for the support of Post-doctoral Researchers”. S.P.G.: would like to thank national funds from 10.13039/501100001871Fundação para a Ciência e a Tecnologia, IP, in the scope of the project UIDP/04378/2020 of the Research Unit on Applied Molecular Biosciences–UCIBIO and the project LA/P/0140/2020 of the Associate Laboratory Institute for Health and Bioeconomy–i4HB. B.A.G.: This article was supported by the 10.13039/501100004410Scientific and Technological Research Council of Turkey with the project number of 121R100. L.L.B: is supported with funds from the Ministry of Education, Science and Youth of Sarajevo Canton, grant ref. 27-02-35-33087-6/24. M.C. is supported with funds from the 10.13039/501100004837Ministerio de Ciencia e Innovación (grant PID2020-115979RR-C32). A.R.D.-M. and M.C. thank the 10.13039/100019007Government of the Canary Islands -– PROMOTUR Turismo de Canarias (Actuaciones de Cohesión de Destino - Canarias Ecoínsulas II), and specifically the project "Islas Canarias, naturaleza marina singular: Salud y bienestar". I.D. This article was supported by the Scientific and Technological Research Council of Turkey (TUBİTAK) with the project number of 124M105.

The authors would like to thank Dr. Hordur G. Kristinsson, former Director of Research & Innovation at Matís and one of the founders of UNA skincare in Iceland, for all his valuable work and scientific contribution to the seaweed research and application development. We would also like to thank Aðalheiður Ólafsdóttir and Halla Halldórsdóttir at Matís for carrying out the human intervention study.

## Author contributions

Conceptualization, A.R.; Visualization, A.R., M.C., and A.R.D.-M.; Writing, all.

## Declaration of interests

The authors declare no competing interests.
