# Supplementary material for: Marine cosmetics and the blue bioeconomy: From sourcing to success stories
Source: iScience. 2024 Nov 6;27(12):111339. doi: 10.1016/j.isci.2024.111339 (PMC11625311; doi:10.1016/j.isci.2024.111339)
Supplement: Document S1. Document S1–S3 and Tables S1 [file mmc1.pdf]

**iScience, Volume *27***

## Supplemental information

# Marine cosmetics and the blue bioeconomy: From sourcing to success stories

**Ana Rotter, Despoina Varamogianni-Mamatsi, Alenka Zvonar Pobirk, Mirjam Gosenca Matjaž, Mercedes Cueto, Ana R. Díaz-Marrero, Rósa Jónsdóttir, Kolbrún Sveinsdóttir, Teresa S. Catalá, Giovanna Romano, Bahar Aslanbay Guler, Eylem Atak, Maja Berden Zrimec, Daniel Bosch, Irem Deniz, Susana P. Gaudêncio, Ernesta Grigalionyte-Bembič, Katja Klun, Luen Zidar, Anna Coll Rius, Špela Baebler, Lada Lukić Bilela, Baruch Rinkevich, and Manolis Mandalakis**

# Document S1: Additional regulatory framework on cosmetics in the European Union

The Cosmetics Regulation (EC) No. 1223/2009 clearly defines which substances may be used in cosmetic products without restriction, which are prohibited, and which may be used under special conditions. The annexes of the Cosmetics Regulation (EC) No. 1223/2009 list[1]:

- 1328 prohibited substances (Annex II).
- 256 substances that may not be contained in cosmetic products unless they are subject to restrictions (Annex III)
- 153 colorants that may be used in cosmetic products (Annex IV)
- 57 Preservatives that may be used in cosmetic products (Annex V)
- 28 UV filters that may be used in cosmetic products (Annex VI).

If the substances are not included in the approved list, the ingredient must undergo a safety and toxicological assessment by the Scientific Committee on Consumer Safety (SCCS)[2]. A safety dossier must therefore be submitted for authorization as a cosmetic substance[3]. In addition, the law stipulates that a specific safety assessment must be carried out for cosmetic products intended for children under the age of three[2,4–6].

The safety assessment of cosmetic products I shall be carried out by a person holding a diploma or other evidence of formal qualifications awarded on completion of a university course of theoretical and practical study in pharmacy, toxicology, medicine or a similar discipline, or a course recognised as equivalent by a Member State. The safety assessment may be carried out internally if the appropriate personnel are available. If this is not the case, the safety assessment must be carried out by a suitably qualified third party[5].

The Cosmetic product safety report is composed of 2 parts:

| **Part A** | **Part B** |
|---|---|
| 1. Quantitative and qualitative composition of the cosmetics product | 1. Assessment conclusions |
| 2. Physical/chemical characteristics and stability of the cosmetic product | 2. Labelling warnings and instruction of use |
| 3. Microbiological quality | 3. Reasoning |
| 4. Impurities, traces, information about packaging material | 4. Assessor`s credentials and approval of part B |
| 5. Normal and reasonably foreseeable use. | |
| 6. Exposure to the cosmetic product | |
| 7. Exposure to the substances | |
| 8. Toxicological profile of the substances | |
| 9. Undesirable and resinous undesirable effects | |
| 10. Information on the cosmetic product | |

If a cosmetic product is placed on the market, the responsible person shall maintain a product information file for this product. The product information file shall be kept for a period of ten years from the date on which the last batch of the cosmetic product was placed on the market[3].

The Cosmetics Regulation (EC) No. 1223/2009 also lays down labelling requirements for cosmetic products[1]. In addition to basic information such as the manufacturer's details and expiry date, information on the safety of the cosmetic product must also be included, e.g., particular precautions to be observed in use and a list of ingredients. The labelling of cosmetic products is mandatory in the "ingredients" section of the packaging. The first instruction to adopt a sensible consumer attitude is thus to avoid cosmetics for which no information on the ingredients present is given under their INCI (International Nomenclature Cosmetic Ingredient) name, classified in descending order of weight[7].

Another important aspect in connection with the labelling of cosmetic products are the claims. Claims in the form of texts, names, trademarks, images and figurative or other signs shall not be used to imply that these products have characteristics or functions that they do not have[1,8,9]. For example, the phrases "does not contain" with regard to a particular class of ingredient, or "0%", which were purely marketing ploys that contributed to the spread of misconceptions, have been banned since 1 July 2019[7].

Particular attention should be paid to the terms "natural" and "organic". The Cosmetics Regulation (EC) No. 1223/2009 does not define the terms "natural" and "organic" in relation to cosmetics[1]. A single harmonized European label for natural and organic cosmetics seems essential. Some labels have been grouped together in a harmonized COSMOS – COSMetics Organic Standard – label that offers an international standard for natural and organic cosmetics, and harmonizes among other things the BDIH, Cosmébio, Ecocert, ICEA and Soil Association standards[7].

Regardless of the actual label, the common fundamental principles respect the following principles:

1. Authorized and listed ingredients and manufacturing processes,
2. Restricted use of synthetic ingredients,
3. Prohibited and listed ingredients and manufacturing processes. The main prohibitions are:
    - No testing on animals (as with all European cosmetics);
    - Must not contain synthetic perfume or dye;
    - Must contain absolutely no synthetic preservatives such as parabens or phenoxyethanol;
    - Must not contain any ingredients from petrochemical processing (paraffins, silicone, and PEG), genetically modified organisms (GMOs), substances treated with ionizing radiation or nanoparticles[7].

All cosmetic products available on the market should be monitored by the EU Member States. According to the Cosmetics Regulation (EC) No 1223/2009, Member States are obliged to regularly monitor cosmetic products, economic operators and Product Information Files as well as carry out physical cosmetic products and laboratory controls[1].

Any legal or natural person who ensures that any cosmetic product placed on the EU market complies with all relevant regulations (known as the Responsible Person) and any distributor must ensure that any serious undesirable effects caused by the cosmetic product are reported to the competent national authorities within 20 calendar days from the date on which an employee of the Responsible Person or distributor becomes aware of the serious undesirable effects, regardless of their role or function[9–11].

## Document S2: Sustainable harvesting of marine biomass: harvesting of macroalgae in Iceland

There are around 300 seaweed species around Iceland. However, only few of them have been harvested and utilized. Out of the estimated several millions of metric tons of seaweed biomass in Iceland, less than 20,000 tons of marine seaweeds are harvested, representing a minute fraction of what could be safely harvested and utilized. Since 1980, *Ascophyllum nodosum* has only been harvested in Breiðafjörður in a sustainable way amounting between 10,000 and 18,000 tons per year. With increased interest in utilizing seaweed in Iceland, especially *A. nodosum*, the Marine and Freshwater Research Institute (MFRI), at the request of the Ministry of Industries and Innovation, conducted a study on the distribution and quantity of this seaweed in Breiðafjörður. In line with the necessary precautionary principles, MFRI recommended that the total harvest of *Ascophyllum* in Breiðafjörður should not exceed 40,000 tons per year for the the period between 2018–2022.

https://www.hafogvatn.is/is/moya/news/radgjof-um-aflamark-a-klothangi

The Founding Regulation 1007/2024 on harvesting of seaweed for commercial purpose within Iceland's fishing territory prohibits the commercial harvesting of seaweed without the required permits from the Directorate of Fisheries. Permits can be issued under specific conditions, such as development of a utilization plan for the harvesting of seaweed, specification of equipment to be used for harvesting, the quantity of seaweed to be harvested, the locations of harvesting, and, where applicable, the identification of areas to promote regrowth. The plan must be based on an assessment of biomass and accessibility. The plan must be updated before harvesting begins each year.

https://island.is/reglugerdir/nr/1007-2024

Document S3: Gene expression for selected genes (ATOX1, CCL5 (RANTES), DHCR24, FOXM1, GPX1, GPX4, PDLIM1, PRDX5, SIRT2, SOD2), involved in antioxidative, regulatory, anti-inflammatory processes, showing the potential of *Tetraselmis suecica* in cosmetic formulations.

*Tetraselmis* alone

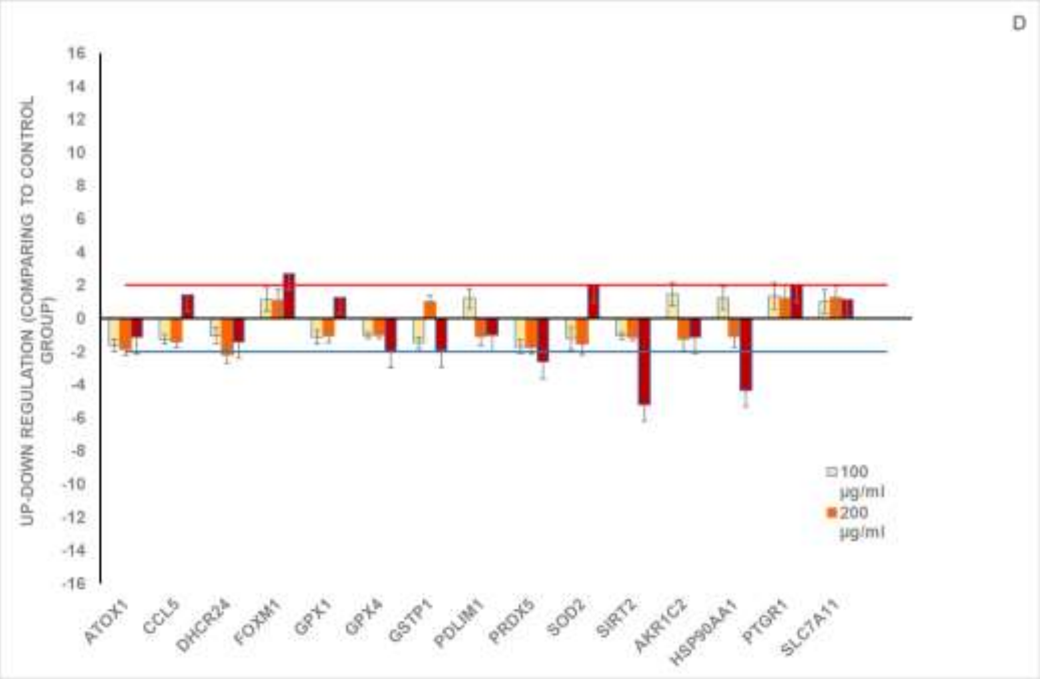


Graph gene expression 100 µg/mL

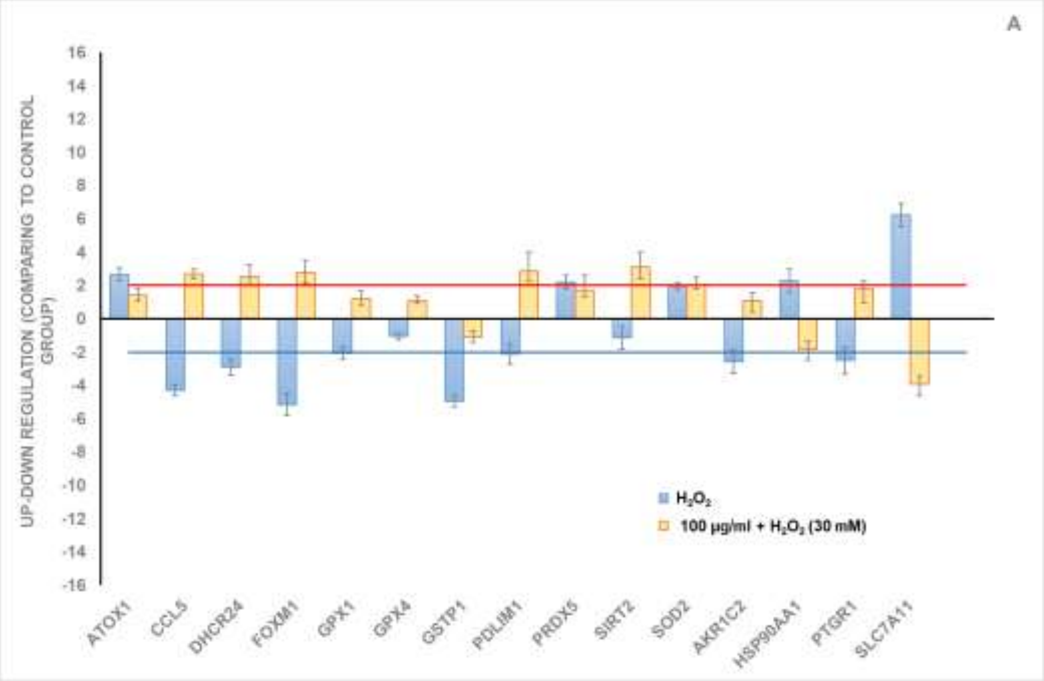

Graph gene expression 200 µg/mL

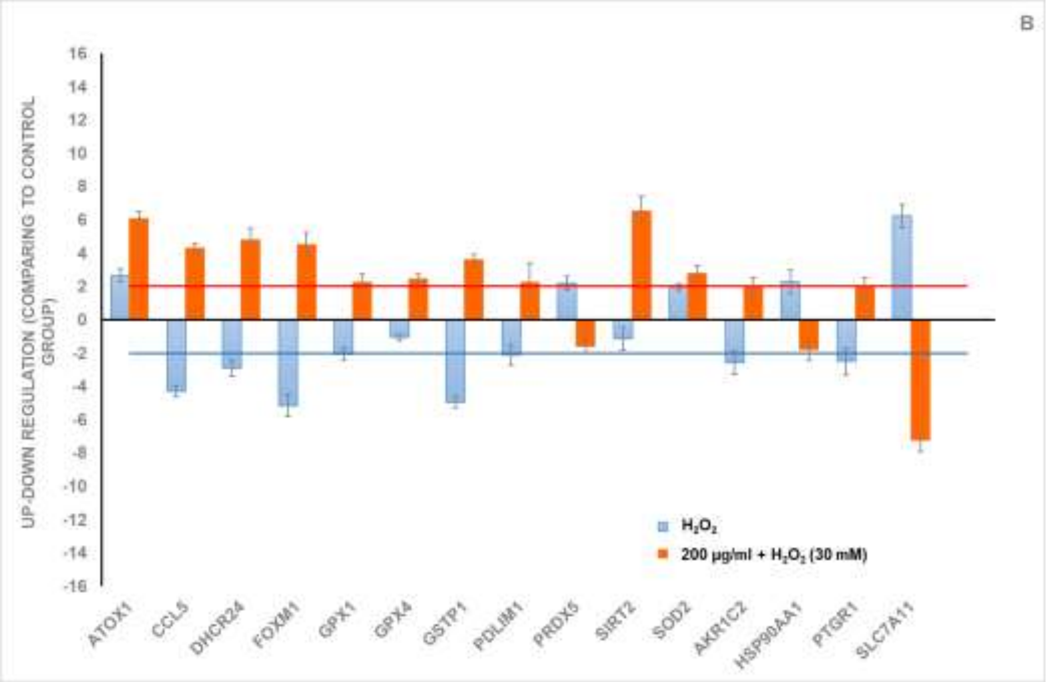


Graph gene expression 400 µg/mL

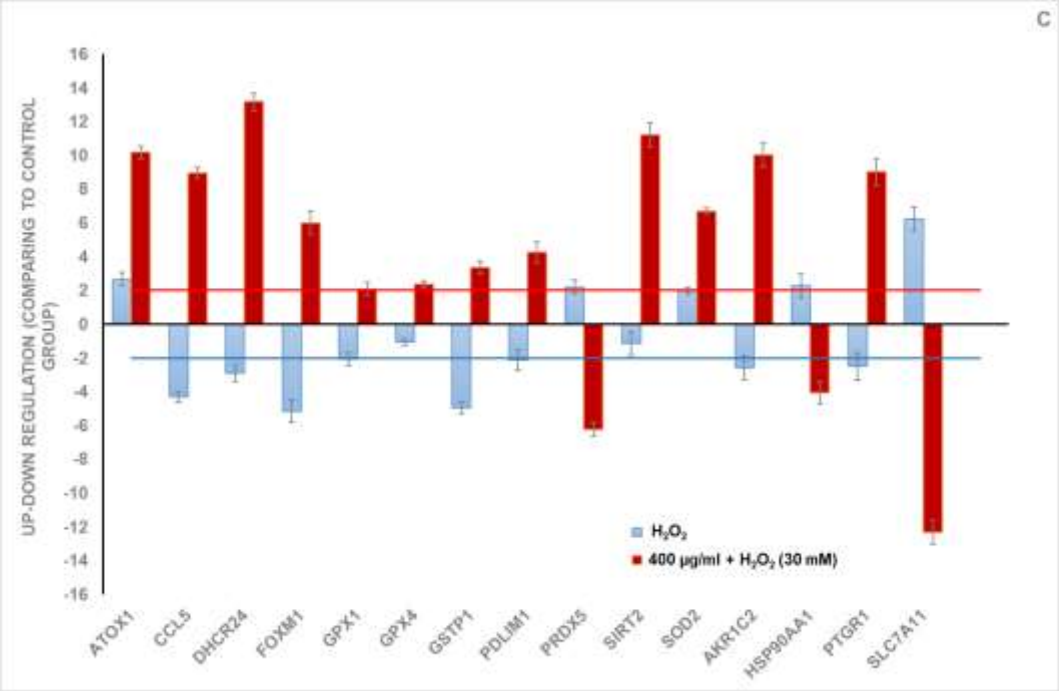

## Table S1: Some chemicals used in cosmetic industries along with their side effects[12,13]

| **Chemical** | **Effect of Chemical** | **Products** |
|---|---|---|
| 1,4-Dioxane | Carcinogen linked to organ toxicity | Shampoos<br>Liquid soaps<br>Bubble baths<br>Hair relaxers |
| Acrylates (ethyl acrylate, ethyl methacrylate, and methyl methacrylate) | Eye and respiratory tract irritants<br>Allergic contact dermatitis | Acrylic nails<br>Nail enhancing polishes |
| Benzophenone | Human carcinogen<br>Endocrine disruptor | Lip balm<br>Nail polish |
| Butylated hydroxy anisole (BHA) and butylated hydroxytoluene (BHT) | Endocrine disruptor | Lip and hair products<br>Sunscreen<br>Antiperspirants<br>Deodorants<br>Fragrances<br>Creams |
| Carbon black | Carcinogen,<br>Lung disease,<br>Hyperhomocysteinemia<br>Platelet hyperactivity, | Eyeliner<br>Mascara<br>Lipstick<br>Nail polish<br>Eye shadow<br>Brush-on-brow<br>Blushers<br>Rouge |
| Coal tar | Carcinogen<br>Skin cancer | Scalp treatments,<br>Soaps,<br>Hair dyes<br>Lotions |
| Ethanolamine compounds | Hepatocarcinogenic,<br>Organ toxicity | Soaps,<br>Shampoos,<br>Hair conditioners and dyes,<br>Lotions,<br>Shaving creams,<br>Paraffin and waxes,<br>Household cleaning products,<br>Pharmaceutical ointments,<br>Eyeliners,<br>Mascara,<br>Eye shadows,<br>Blush,<br>Make-up bases |
| Ethoxylated ingredients | Carcinogen,<br>Lymphoma and leukemia, stomach, and breast cancers | Shampoo,<br>Liquid soap,<br>Bubble bath<br>Hair relaxers |

| | | |
|---|---|---|
| Formaldehyde | Myeloid leukemia,<br>Cancers of the paranasal sinuses, nasal cavity, and nasopharynx | Nail polish<br>Nail glue<br>Eyelash glue<br>Hair gel<br>Hair-smoothing products<br>Baby shampoos<br>Body soaps<br>Body wash<br>Color cosmetics |
| Parabens | Estrogenic and genotoxic activity | Shampoos<br>Conditioners<br>Lotions<br>Facial<br>Shower cleansers<br>Scrubs |
| Octinoxate | Breast cancer | Hair color products<br>Shampoos<br>Sunscreen<br>Lipsticks<br>Nail polish<br>Skin creams |
| Phenoxyethanol | Life-threatening allergic reactions,<br>Eczema,<br>Nervous system function | Moisturizers<br>Eye shadows<br>Foundation<br>Sunscreens<br>Conditioners<br>Mascaras<br>Eyeliners<br>Shampoos<br>Lip gloss<br>Concealers<br>Body wash<br>Hand creams<br>Blush<br>Haircolors<br>Hair sprays<br>Lip balms<br>Lotions<br>Nailpolish<br>Baby wipes<br>Baby lotions<br>Shaving<br>Creams<br>Deodorants |

## References


1. European Commission (EC). Regulation (EC) No 1223/2009 Of The European Parliament And Of The Council on cosmetic products. (2009).

2. SCCS Notes of guidance for the testing of cosmetic ingredients and their safety evaluation - 12th revision (2024). European Commission - Public Health. https://health.ec.europa.eu/publications/sccs-notes-guidance-testing-cosmetic-ingredients-and-their-safety-evaluation-12th-revision_en.

3. Manful, M.E., Ahmed, L., and Barry-Ryan, C. (2024). Cosmetic Formulations from Natural Sources: Safety Considerations and Legislative Frameworks in the European Union. Cosmetics *11*, 72. https://doi.org/10.3390/cosmetics11030072.

4. European Directorate for the Quality of Medicines & HealthCare (EDQM), 2023. Safe cosmetics for young children. (2024). Council of Europe. https://freepub.edqm.eu/publications/AUTOPUB_20/detail.

5. Cosmetic Toiletry and Perfumery Association (CTPA 2021). Supplying Cosmetic Products on the EU and NI Markets? (2021).

6. Resolution CM/ResAP (2012) on safety criteria for cosmetic products intended for infants. (2024). https://search.coe.int/cm?i=09000016805cae1c.

7. Barbaud, A., and Lafforgue, C. (2021). Risks associated with cosmetic ingredients. Annales de Dermatologie et de Vénéréologie *148*, 77–93. https://doi.org/10.1016/j.annder.2020.04.027.

8. European Commission (EC). Commission Regulation (EU) No 655/2013 laying down common criteria for the justification of claims used in relation to cosmetic products. (2013).

9. European Commission (EC). Technical document on cosmetic claims. Technical document on cosmetic claims (version of 3 July 2017). (2017).

10. Renner, G., Audebert, F., Burfeindt, J., Calvet, B., Caratas-Perifan, M., Leal, M., Gorni, R., Long, A., Meredith, E., O'Sullivan, Ú., et al. (2017). Cosmetics Europe Guidelines on the Management of Undesirable Effects and Reporting of Serious Undesirable Effects from Cosmetics in the European Union. Cosmetics *4*, 1. https://doi.org/10.3390/cosmetics4010001.

11. European Commission (EC). Serious undesirable effects reporting guidelines (2013).

12. Petric, D. (2021). Review of Toxic Chemicals in Cosmetics. Preprint, https://doi.org/10.14293/S2199-1006.1.SOR-.PPKO7OD.v1 https://doi.org/10.14293/S2199-1006.1.SOR-.PPKO7OD.v1.

13. Stuart Creton, M.R.W., Ian C. Dewhurst, Lesley K. Earl, Sean C. Gehen, Robert L. Guest, Jon A. Hotchkiss, Ian Indans, and Billington, R. (2010). Acute toxicity testing of chemicals—Opportunities to avoid redundant testing and use alternative approaches. Critical Reviews in Toxicology *40*, 50–83. https://doi.org/10.3109/10408440903401511.
